# Transcriptomic profiling reveals SARS-CoV-2-infected humanized MHC mice recapitulate human post vaccination immune responses

**DOI:** 10.3389/fcimb.2025.1634577

**Published:** 2025-08-01

**Authors:** Siyue Li, Xuelian Han, Ruolan Hu, Keyu Sun, Min Li, Yuan Wang, Guangyu Zhao, Mengzhe Li, Huahao Fan, Qi Yin

**Affiliations:** ^1^ School of Life Sciences, Tianjin University, Tianjin, China; ^2^ College of Life Science and Technology, Beijing University of Chemical Technology, Beijing, China; ^3^ State Key Laboratory of Pathogen and Biosecurity, Academy of Military Medical Sciences, Beijing, China; ^4^ Public Health School, Mudanjiang Medical University, Mudanjiang, China; ^5^ State Key Laboratory of Synthetic Biology, Tianjin University, Tianjin, China; ^6^ Frontiers Science Center for Synthetic Biology (Ministry of Education), Tianjin University, Tianjin, China; ^7^ State Key Laboratory of Component-based Chinese Medicine, Tianjin University of Traditional Chinese Medicine, Tianjin, China

**Keywords:** hMHC mice, SARS-CoV-2, COVID-19, Humanization, Transcriptome

## Abstract

**Background:**

The ongoing COVID-19 pandemic caused by SARS-CoV-2 remains a critical global health priority, with persistent socioeconomic ramifications despite its reclassification from Public Health Emergency of International Concern (PHEIC) status. While humanized major histocompatibility complex (hMHC) murine models have been extensively utilized in oncological research, their application in virological studies-particularly for coronavirus pathogenesis-remains underexplored.

**Methods:**

This study systematically characterized immune responses in SARS-CoV-2-challenged hMHC mice lung tissues through comparative transcriptomic profiling, combined with functional enrichment and PPI network analyses.

**Results:**

Key findings demonstrate that hMHC mice exhibit enhanced immunological activation relative to wild-type controls, particularly in IFN-γ signaling pathways and neutrophil mobilization dynamics that closely parallel human post-vaccination responses. Comparative analysis with human whole blood RNA-seq datasets revealed that hMHC mice exhibit both high reproducibility in transcriptomic profiles and significant similarity to human immune responses across innate and adaptive immunity.

**Conclusions:**

These results confirm that the hMHC murine model can serve as an effective platform for vaccine research, providing a theoretical foundation for the application of humanized MHC mice and offering new insights into viral infection mechanisms and the development of novel vaccines.

## 1 Introduction

Since the discovery of Severe Acute Respiratory Syndrome Coronavirus 2 (SARS-CoV-2) in late 2019, there have been more than 770 million confirmed cases worldwide and 7.05 million deaths (https://www.who.int/), with profound impacts on the global economy and society. SARS-CoV-2 cellular entry is mediated by spike protein binding to angiotensin-converting enzyme 2 (ACE2) receptors (Jackson et al., 2022). The limited binding compatibility between murine ACE2 (mACE2) and SARS-CoV-2 spike proteins has necessitated widespread adoption of human ACE2 (hACE2) transgenic mouse models for investigating viral pathogenesis, therapeutic interventions, and vaccine development. However, the high susceptibility of hACE2 transgenic mice to SARS-CoV-2 frequently results in severe pathological manifestations post-infection, potentially obscuring subtle genetic-level modifications during disease progression.

The major histocompatibility complex (MHC), known as the human leukocyte antigen (HLA) system in humans, constitutes a genetically conserved cluster encoding histocompatibility antigens essential for adaptive immunity. Functioning not only as a pivotal regulator of immune homeostasis but also demonstrating pathological activation in autoimmune cascades, HLA orchestrates both physiological immune surveillance and detrimental autoimmune reactions across diverse disease contexts (Dendrou et al., 2018). Recent advancements in humanized MHC (hMHC) murine models have primarily facilitated investigations into autoimmune disorders, cancer immunotherapies, and drug immunogenicity, with predominant emphasis on HLA class II antigen systems for identifying immunodominant epitopes and deciphering HLA class II-restricted immune regulation (Reipert et al., 2009; Lee et al., 2019). Despite these developments, hMHC models remain underexploited in virological research, particularly for modeling human antiviral immune responses and accelerating vaccine discovery. This limitation stems from interspecies divergence in MHC-mediated antigen presentation – notably the differential peptide-binding repertoires between human HLA and animal MHC molecules – necessitating refined humanized models capable of authentically presenting human vaccine antigens. Such models are crucial for systematically evaluating vaccination strategies and precisely assessing cytotoxic T lymphocyte (CTL) epitope efficacy (Pajot et al., 2004; Sun et al., 2018).

Experimental evidence demonstrates that transgenic expression of human *HLA-A2* (MHC class I) significantly enhances antigen-specific responses of human CD8+ T cells in murine models to Epstein-Barr virus (EBV) (Strowig et al., 2009; Shultz et al., 2010) and dengue virus (DENV) (Jaiswal et al., 2009). Notably, *HLA-A2*-transgenic humanized mice infected with EBV generate antigen-specific T cells targeting viral antigens. These effector T cells dominate latent-phase antigen-specific T cell populations, exhibiting response characteristics remarkably analogous to those observed in T cells from human EBV carriers (Strowig et al., 2009), thereby confirming the capacity of hMHC mice to partially recapitulate post-viral infection immune responses in humans. Furthermore, *HLA-DR4* (MHC class II) transgenic mice exhibit enhanced human immune cell reconstitution accompanied by comprehensive improvements in immunological functionality, including effective immunoglobulin class-switching and significantly elevated human IgG antibody response levels (Covassin et al., 2011; Danner et al., 2011).

In this investigation, we employed a genetically engineered humanized MHC (hMHC) murine model developed through embryonic stem cell-targeted modification of endogenous β2-microglobulin (*β_2_m*) and *H2-Ab1* loci. Insertion of a neomycin phosphotransferase (Neo) selection marker into these genetic regions resulted in functional ablation of murine MHC class I and II antigen presentation systems. Subsequent transgenic introduction of human HLA-A*02:01 and *HLA-DR1* genes established HLA-restricted immunity, wherein all antigen-specific CD8+ cytotoxic T lymphocytes (CTLs) and CD4+ helper T cells-including those mediating virus-directed neutralizing antibody production-became strictly dependent on human HLA molecules rather than murine MHC complexes for activation and functionality (Lee et al., 2019). Notably, while murine angiotensin-converting enzyme 2 (mACE2) receptors in hMHC mice exhibit low SARS-CoV-2 binding affinity and cannot support viral replication (Sun et al., 2020; Le Chevalier et al., 2023), the transgenic HLA-A*02:01 effectively recognizes SARS-CoV-2 nucleocapsid proteins to initiate coordinated immune responses (Chatzileontiadou et al., 2021). Furthermore, spike protein exposure induces localized inflammatory cascades independent of human ACE2 (hACE2) receptor engagement in this model (Umar et al., 2022). These unique immunological features enable mechanistic dissection of immune perturbations that conventional hACE2-dependent models might obscure. By leveraging this system, we systematically profiled immune activation patterns in the absence of overt pathogenicity post-MHC humanization, thereby simulating authentic human post-vaccination immune states against COVID-19.

## 2 Results

### 2.1 Verification of successful construction of hMHC mouse model

**Figure 1:**
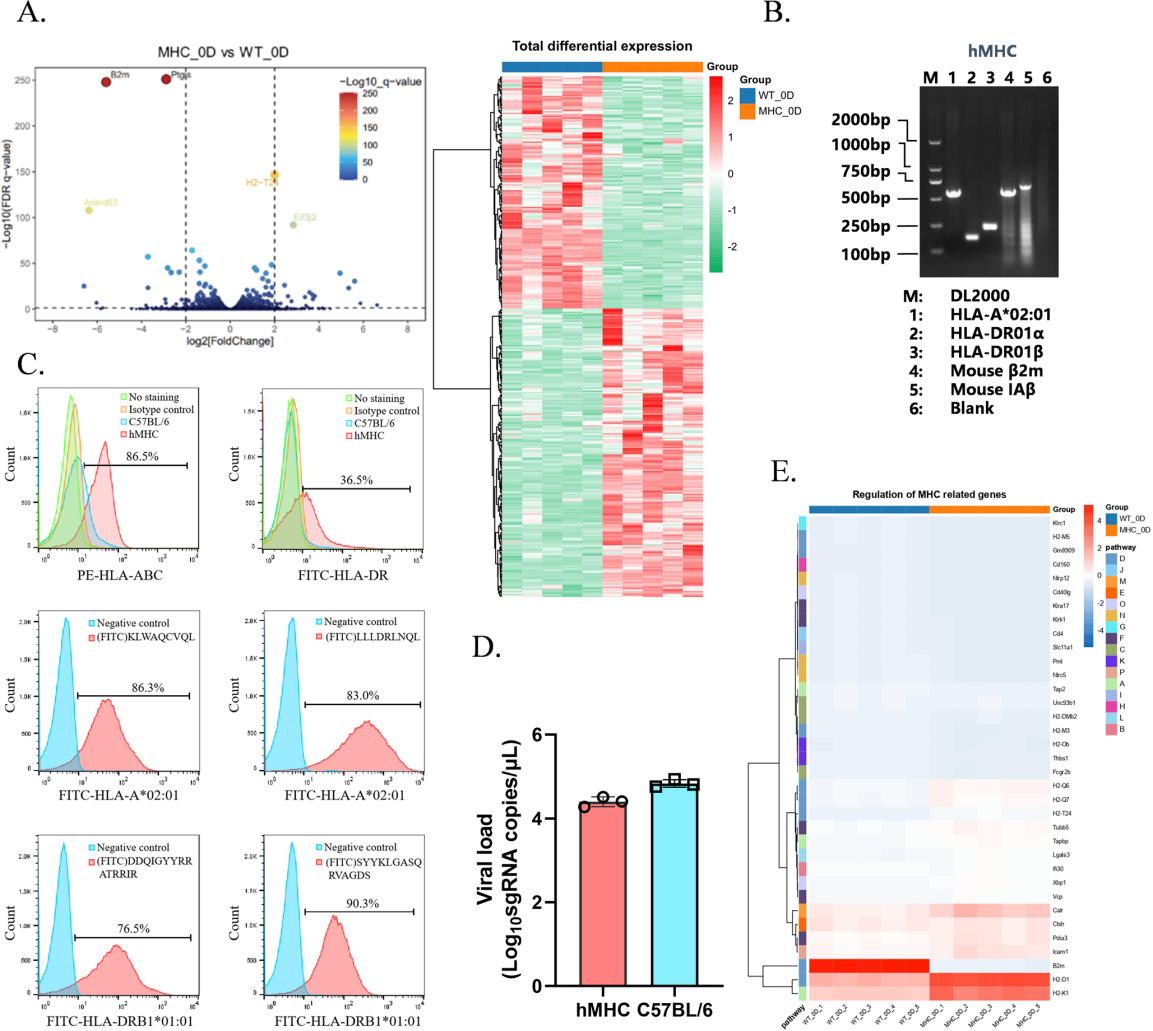
Verification of the successful construction of the hMHC murine model. **(A)** Volcano Plot and Heatmap Analysis at 0-day. We generated a volcano plot and a heatmap to compare the genomic profiles of hMHC mice and wild-type controls at 0-day. *β_2_m*, *Ankrd63*, and *Ptgis* exhibited down-regulation in the volcano plot. Among them, blue represents the hMHC mice and orange represents the wild-type controls; **(B)** DNA validation of the hMHC mouse genotype. DNA validation was performed to confirm the genotype of the hMHC mice. The marker is denoted as “M”, while lane 1, 2, 3, 4, and 5 represent the amplified fragments of HLA-A*02:01 (570bp), *HLA-DR01α* (153bp), *HLA-DR01β* (2224bp), mouse *β_2_m* (wild type 260bp, homozygous 600bp), and mouse *IAβ* (wild type 230bp, homozygous 730bp) respectively. Lane 6 is a negative control; **(C)** Expression of HLA-A*02:01 and HLA-DRB1*01:01 genes on the surface of splenic lymphocytes from hMHC mice. Blue represents negative cells, and red represents positive cells; **(D)** Vial load determination by RT-qPCR. Lysed lung tissues from mice were used for vial load determination (n=3). No significant difference in SARS-CoV-2 infection levels between the two groups; **(E)** Heatmap of MHC-related genes at 0-day. Few MHC-related genes showed significant up-regulation.

To simulate and study the human immune response in mice, we used transgenic technology to modify the *β_2_m* and *IAb* genes in mice, whereby the integration of a neo-selection marker induced insertional mutations. This approach enabled the selection of successfully recombined embryonic cells through resistance screening. The insertion of the neo-marker disrupted the full-length sequences of the two genes, blocking the expression of fully functional proteins while allowing gene replication. To validate the successful establishment of the murine model, we first confirmed whether the endogenous *β_2_m* in the hMHC mice had been edited. To this end, we isolated and collected lung tissue samples from 0-day mice (i.e., mice not infected with SARS-CoV-2) and analyzed them for differential gene expression. A total of 343 differentially expressed genes (P < 0.05 and |Log2 (fold change) | > 1) were identified. Genes such as *β_2_m*, *Ankrd63*, and *Ptgis* were down-regulated, while *H2-T24* and *Eif3j2* were up-regulated (**Figure 1A**). It confirmed the successful modification of *β_2_m* in hMHC murine model, shifting MHC restriction from murine to human origin. The down-regulation of *Ankrd63* and Ptgis might be associated with the editing of *β_2_m*, considered to be the background difference caused by the modification of endogenous MHC. Subsequently, we identified the expression of key transgenes (HLA-A*02:01 and *HLA-DR*) by Western blot (WB) and flow cytometry. WB results indicated that HLA-A*02:01 and *HLA-DR* were successfully expressed in the hMHC murine model (**Figure 1B**). By labeling specific peptides restricted by HLA-A*02:01 and HLA-DRB1*01:01 with FITC, we observed that the hMHC murine model could bind to these specific peptides and emit fluorescent signals, confirming that C57BL/6 mice could recognize and present human-specific peptides after humanization (**Figure 1C**). These findings corroborate the successful integration of human HLA-A*02:01 and *HLA-DR* into the mouse genome, validating the successful establishment of the hMHC murine model.

Therefore, we infected hMHC mice and C57BL/6 mice (wild-type) with SARS-CoV-2 (original strain) via Intranasal (IN) inoculation at a TCID50 of 3000. Six days post-infection, neither group exhibited deaths, and there was no significant difference in pathogenicity between the two groups. Notably, hMHC mice showed a slightly lower viral load than wild-type (**Figure 1D**). We speculate that the lack of the hACE2 receptor allows SARS-CoV-2 infection but not efficiently, resulting in limited phenotypic changes. Subsequently, we thoroughly analyzed the regulation of MHC-related pathway genes in two groups at 0 day. Specifically, we observed that most MHC-related pathway genes were unchanged, with only a few showing significant changes (**Figure 1E**). And **Figure 1E** revealed a significant down-regulation of *β_2_m*, which reaffirmed the successful genetic modification of *β_2_m* in the hMHC murine model. Additionally, MHC class I molecule-related genes (e.g., *H2-Q6*, *H2-Q7*, *H2-T24*, *H2-D1*, and *H2-K1*) exhibited an up-regulation trend. This upregulation indicates that upon the introduction of human HLA molecules, the hMHC mice exhibit enhanced activation of MHC class I protein-binding and peptide antigen-binding activities, enabling them to partially recapitulate the human immune response. Collectively, these findings demonstrate the maturity and reliability of the hMHC murine model as a robust research platform, positioning it as an ideal experimental tool for future studies.

### 2.2 Differential gene expression profiles between hMHC and wild-type C57BL/6 mice following infection

To further reveal the molecular characteristics of the immune response in hMHC mice, lung tissue samples were isolated and collected from mice on day 1, 3, and 6 after infection with SARS-CoV-2 for RNA sequencing (RNA-seq) and network analysis. Notably, marked discrepancies in gene expression levels were observable even before infection (0-day) (**Figure 1A**). To ensure the accuracy and reliability of the analysis, this study incorporated a background correction step to identify and quantify the genuine changes in gene expression post-infection. Standardized analyses were performed using 0-day samples as the baseline.

**Figure 2:**
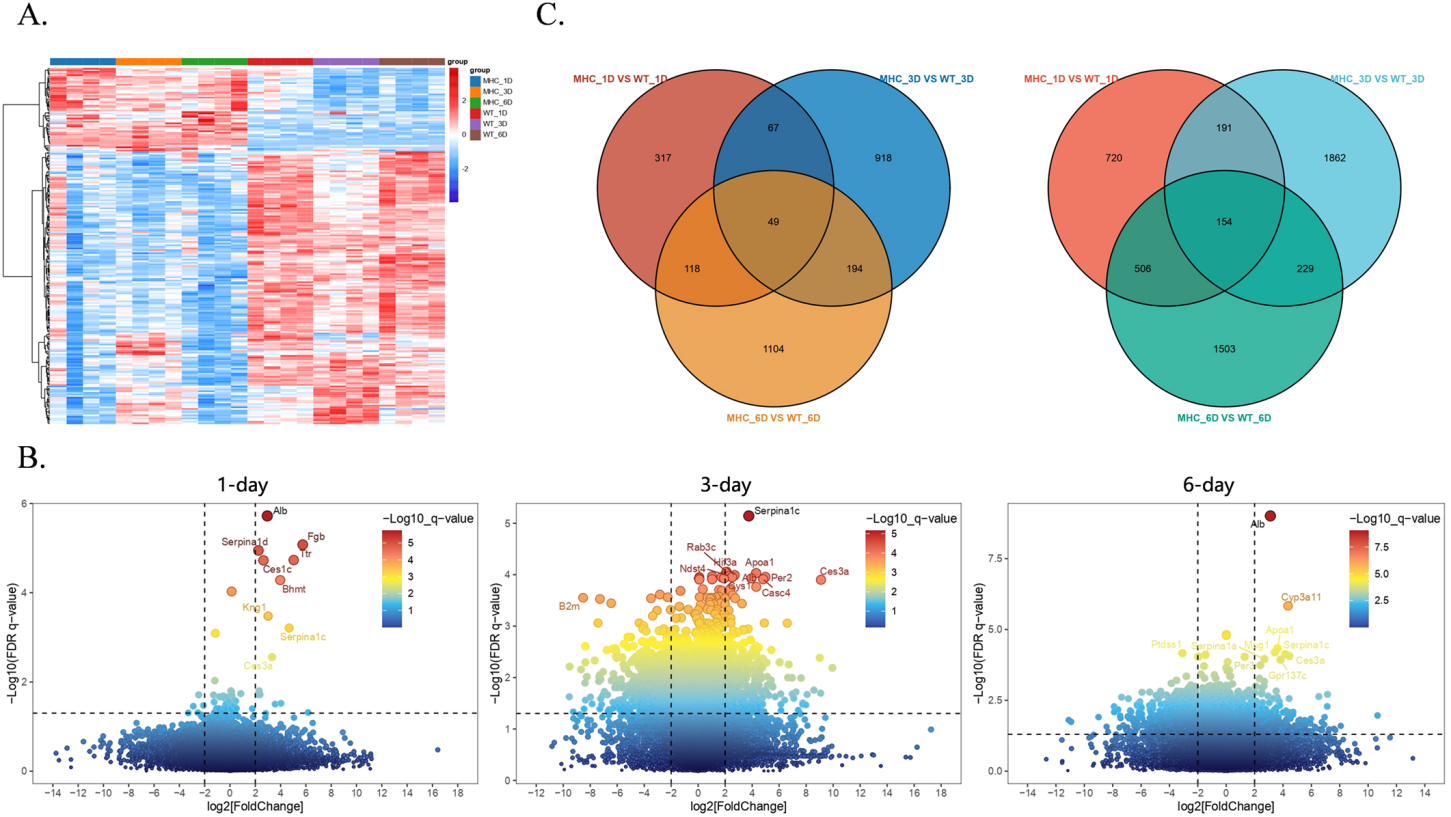
Comparison of transcriptomic differentially expressed genes (DEGs) between the hMHC mice and wild type mice with standardized analyses. **(A)** Heatmap of DEGs between two groups. Most genes exhibited an up-regulation trend, while a few genes were down-regulated. Among them, blue represents the hMHC mice on 1-day, orange represents the hMHC mice on 3 day, green represents the hMHC mice on 6 day, red represents the wild-type controls on 1 day, purple represents the wild-type controls on 3 day, and brown represents the wild-type controls on 6 day; **(B)** Volcano plots of DEGs on 1-day (left), 3-day(middle), and 6-day(right), highlight genes with significant up-regulation over time; **(C)** Venn diagram showed the overlapping gene counts of up-regulated (left) and down-regulated (right) genes between two groups.

First, the obtained transcriptome data were preprocessed and background-corrected. We constructed heatmaps and volcano plots (P < 0.05, |Log2 (fold change) | > 1) to assess the changes in gene expression after SARS-CoV-2 infection (**Figures 2A, B**). The heatmap illustrated that in the two groups of mice, the majority of differentially expressed genes exhibited a downward trend. Some genes were consistently up-regulated, while a negligible number showed a dynamic change of up-regulation and then down. This suggests that the hMHC mice can manage viral infection by mobilizing only a limited number of genes. In the volcano plots, we highlighted genes with sustained differential expression (**Figure 2B**). Gene perturbations were more significant in the early and middle phases of viral infection, with up-regulated genes being particularly prominent (the top ten differential genes were all up-regulated), suggesting that hMHC mice exhibited a stronger immune response against the virus.

The volcano plots showed that the differentially expressed genes at various stages of SARS-CoV-2 infection were mainly associated with lipid metabolism and coagulation pathways. Crucial differentially expressed genes, including *Serpina1*, *Apoa1*, *Alb*, *Kng1*, *Ttr*, *Fgb* and *Ces3a*, have the potential to serve as diagnostic markers. On 1-day, the *Serpina1* family (*Serpina1a*, *Serpina1c* and *Serpina1d*) was observed to be extensively up-regulated. *SERPINA1* belongs to the class of serine protease inhibitors in humans, with predominant activity in the lungs. Following vaccination with ChAdOx1-S, the expressions of *SERPINA3* (an acute-phase protein), complement component 9 and *APOC2* (a cofactor that activates lipoprotein lipase) were up-regulated (Ryan et al., 2023). Stimulation by IFN-α, IL-1, IL-6 and TNF-α triggers the *SERPINA1* gene to produce α-1 antitrypsin (Pintanel-Raymundo et al., 2024). As an acute-phase reactant, its rapid up-regulation during SARS-CoV-2 infection is consistent with the results presented in the volcano plots (**Figure 2B**), indicating that *Serpina1* family genes in hMHC mice exhibit similar trends to those in humans. Throughout the infection, there are other genes up-regulation, including: (a) Carboxylesterase 3A (*Ces3a*), which plays a role in exogenous responses and is associated with obesity and diabetes (Jones et al., 2013); (b) Apolipoprotein A-I (*Apoa1*), which interacts with the S2 domain of the SARS-CoV-2 spike protein (Burnap et al., 2023); (c) Kininogen 1 (*Kng1*), a coagulation regulator that acts upstream in inflammatory responses and also possesses certain anti-adhesion properties (Chavakis et al., 2002). Besides these significantly up-regulated genes, other genes associated with coagulation and various blood functions were also found to be up-regulated in the hMHC mice. Nina H. et al. found that some individuals developed venous thromboembolism and associated thrombocytopenia following administration of the ChAdOx1 nCoV-19 vaccine (Schultz et al., 2021), suggesting that thrombosis might occur in the hMHC mice while participating in inflammatory responses.

**Figure 3:**
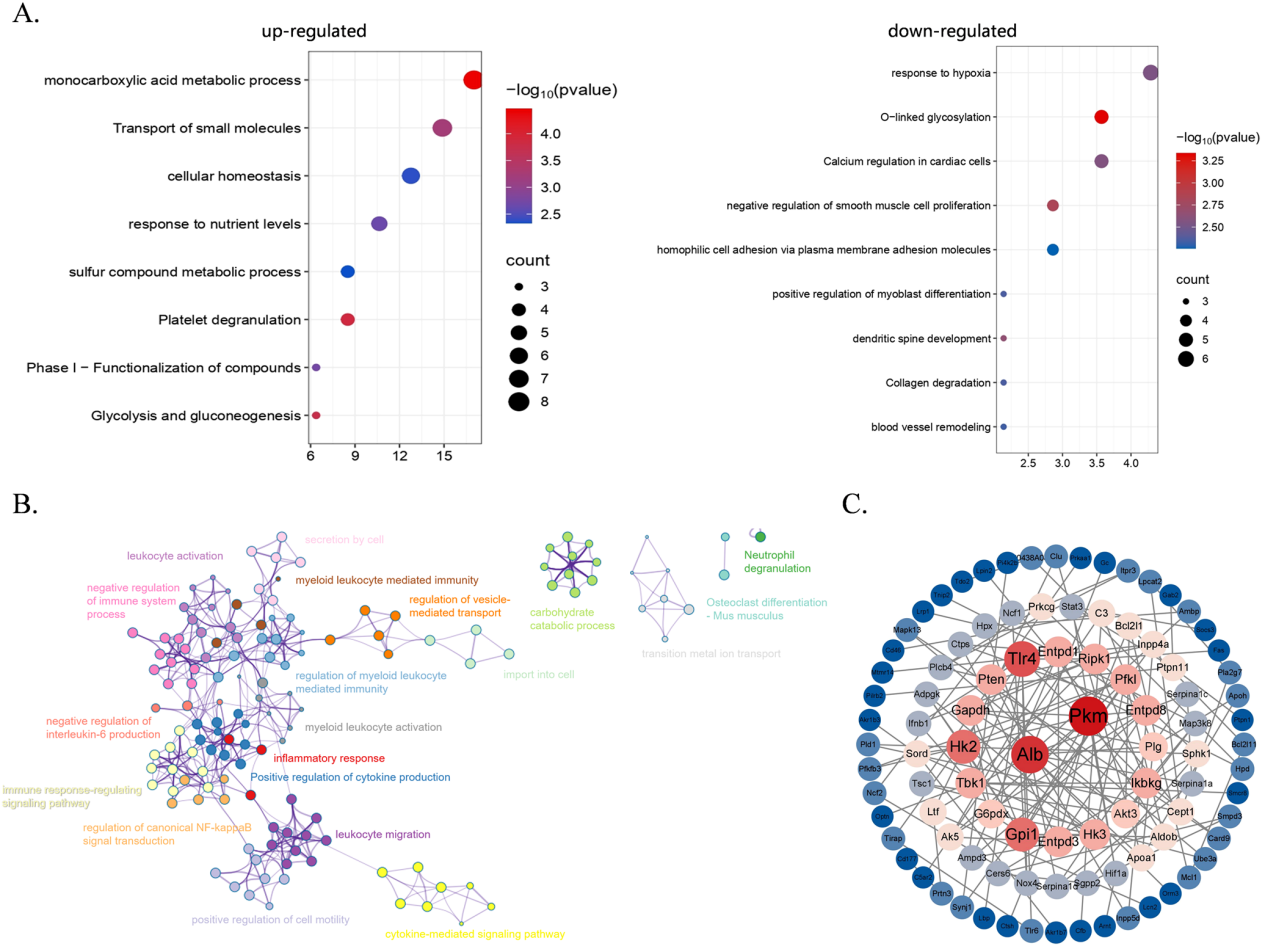
The functional enrichment of differentially expressed genes (DEGs) in the hMHC mice and wild type mice after background subtraction. **(A)** Pathway enrichment analysis of up-regulated (left) and down-regulated (right) differentially expressed genes. Red color means more significant enrichment. The size of the bubble scaled the count of the enriched genes; **(B, C)** Protein-protein interaction network **(B)** and identification of core associated genes **(C)** among the upregulated genes between two groups. In **(C)**, size represents the number of codes associated with genes.

The classification presented by the Venn diagram showed continuously up-regulated and down-regulated genes in two groups of mice on various days of infection (**Figure 2C**). The results indicate that there were more genes down-regulated than up-regulated. Specifically, a total of 49 genes were continuously up-regulated while 154 genes were continuously down-regulated. Then, we carried out Gene Ontology (GO) enrichment analysis on the genes that exhibited persistent changes from the Venn diagram (**Figure 3A**), to further evaluate changes in biological processes in the hMHC mice. GO Biological Processes showed the most significant enrichment. Up-regulated genes were mainly enriched in the monocarboxylic acid metabolic process, transport of small molecules and cell homeostasis. The down-regulated genes were predominantly enriched in the Calcium regulation of cardiomyocytes, negative regulation of smooth muscle cell proliferation, and homophile cell adhesion through plasma membrane adhesion molecules. Furthermore, among all the enriched pathways, the immune pathways interact with one another and have extensive cross-talk with other pathways, including the inflammatory response&、positive regulation of cytokine production&、regulation of canonical NF-κB signal transduction and negative regulation of IL-6 production (**Figure 3B**). In addition, by utilizing the MCODE algorithm for Protein-Protein Interaction (PPI) network analysis, we identified genes within densely connected network components. Our findings revealed that genes such as *Alb, Pkm*, *Tlr4*, *Hk2* and *Gpi1* are extensively interconnected with other genes, most of which are closely associated with inflammatory responses and glycolysis processes (**Figure 3C**). These results further suggest that the hMHC mice can exhibit a more robust immune response, particularly an innate immune response after SARS-CoV-2 infection.

In general, the differences between the hMHC mice and wild-type C57BL/6 mice, after standardized analyses using day 0 samples as the baseline, were mainly manifested in small molecule metabolism and inflammatory responses. These discoveries further underscore the critical importance of innate immune activation and metabolic regulation after infection.

### 2.3 The immune reaction in hMHC mice serves as a model for the human immune response post-vaccination

Due to the natural lack of hACE2 receptors in mice, SARS-CoV-2 failed to induce significant pathological phenotypes. Nevertheless, the murine immune system still responds to the virus. To better simulate the human immune responses after vaccination, we utilized the hMHC murine model rather than the traditional ACE2 transgenic murine model. Since the immune system of the hMHC murine model is partially humanized and showed an up-regulated immune response at 0-day (**Figure 1A**), we chose to directly compare the original transcriptomic data of the hMHC mice with that of the vaccinated mice/humans without subtracting background differences. This approach enabled us to evaluate the reproducibility of the data and explore their functional implications in the population. By correlating these signaling pathways with those associated with COVID-19 vaccines, we aim to reveal the similarities and differences between the immune responses of the hMHC mice and humans, thereby providing more in-depth understanding and valuable insights for vaccine research.

**Figure 4:**
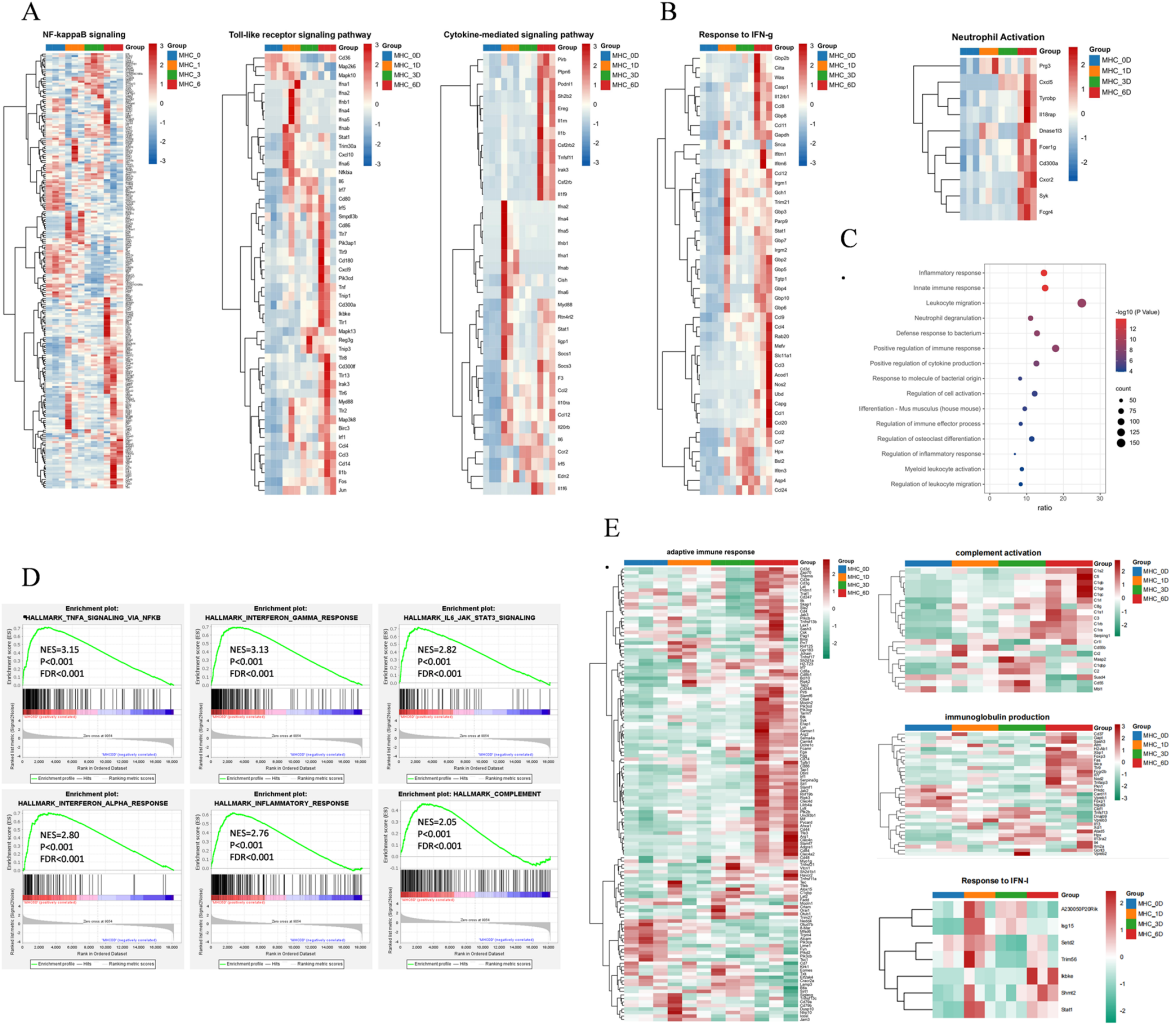
Longitudinal comparison trends in the hMHC mice. **(A)** Heatmaps of the selected signaling pathways in the hMHC mice on various days post-infection; **(B)** Heatmap of IFN-γ (right) and neutrophil activation signaling pathways (left) in the hMHC mice on various days post-infection; **(C)** GO pathway analysis of hMHC mice on day 6; **(D)** GSEA of the selected pathways in the hMHC mice on 6-day, with the database downloaded from the Molecular Signatures Database (MSigDB); **(E)** Heatmaps of the selected signaling pathways (based on reference) in the hMHC mice.

First, we compared the transcriptome data of hMHC mice longitudinally. Previous studies have shown that the SARS-CoV-2 N protein causes acute lung injury in mice by activating the NF-κB pathway (Xia et al., 2021). In the hMHC mice, although NF-κB activation was observed on 6-day, there was merely a threefold increase in the expression of *Nlpr3* inflammasomes. These findings suggested that it was insufficient to trigger excessive pulmonary inflammation (**Figure 4A**) with a limited ability to establish a productive viral infection in the hMHC mice. Simultaneously, we observed the rapid activation of Toll-like receptors and cytokine-mediated signaling pathways following pathogen invasion (**Figure 4A**; **Supplementary Figure S1A**). Specifically, on the 1-day post-infection, a significant up-regulated in the expression levels of inflammatory cytokines, indicating that the immune response mechanism in the mice was swiftly engaged. Such rapid immune activation is essential for defending against viral infections. It promptly mobilizes immune cells, stimulates the production of antiviral cytokines, and initiates other immune defense pathways to effectively control and clear the virus during the early stages of infection. Moreover, the signaling pathways associated with IFN-γ and neutrophil activation exhibited a sustained up-regulation trend over 6-days, similar to the findings observed in K18-hACE2 mice vaccinated with the MVA-S vaccine (Gómez-Carballa et al., 2023) (**Figure 4B**). GO analysis also revealed that up-regulated genes in the hMHC mice were enriched in functions related to the inflammatory response, innate immunity and neutrophil degranulation (**Figure 4C**). Gene Set Enrichment Analysis (GSEA) further confirmed that differentially expressed genes were highly enriched in multiple key signaling pathways, including IFN-γ, TNF-α/NF-κB signaling, IFN-α, inflammatory response, complement system and IL-6/JAK/STAT3 signaling pathways (**Figure 4D**). Furthermore, it affected the cell cycle-associated regulatory pathways with SARS-CoV-2 infected (**Supplementary Figure S1B**).

In a previous vaccine study, experimenters administered a single dose of the mRNA BNT162b2 vaccine to hamsters and treated them with different SARS-CoV-2 variants. Transcriptome data showed that differentially expressed genes involved in the regulation of T cells, memory T cells and natural killer cells, antigen presentation, B cell maturation and proliferation, macrophage activation, monocyte chemotaxis, as well as certain genes associated with DNA repair/replication, were up-regulated by vaccination rather than by infection. Notably, genes such as *Cyp26b1*, *Dbp*, *Kcng1*, *Cdca7l*, *Paxip1*, *Stxbp2* and *Gpi1* were also observed to be significantly up-regulated (p<0.05) in the hMHC mice on various days post-infection. GO analysis showed that these genes were mainly involved in the positive regulation of immune effector process (GO:0002699). In addition, a transcriptomic study of the SARS-CoV-2 BNT162b2 vaccine booster in humans suggests that differential splicing regulatory mechanisms (primarily involving HLA alleles) are crucial in vaccine immunogenicity (Santos-Rebouças et al., 2024).

In this study, we replicated the enrichment of the aforementioned genes from the hMHC murine model, focusing on several key pathways, including the IFN-γ signaling, immune system, innate immune response, neutrophil degranulation, interferon signaling, interferon α and β signaling and endoplasmic reticulum phagosome pathways. It was observed that the adaptive immune responses, complement activation and immunoglobulin production were significantly enriched after 6-day, aligning with the gene expression patterns observed approximately 6 days following the administration of the ChAdOx1-S vaccine (**Figure 4E**) (Ryan et al., 2023). Another feature of vaccine response is the activation of IFN pathways, including the IFN-I signaling pathway and response to IFN-γ. Wang X. et al. discovered that the expression changes of genes associated with these pathways are transient, typically subsiding by day 2 or day 7 (Wang et al., 2022). We enriched the two pathways mentioned above. We found that the majority of genes began to exhibit a down-regulated by 6-day (**Figures 4A, E**). Overall, the hMHC transgenic murine model is capable of precisely mirroring the human immune system’s response to SARS-CoV-2. Consequently, it offers a more accurate and reliable animal model for vaccine research, facilitating a deeper understanding of the immune mechanisms involved and potentially accelerating the development of effective vaccines.

## 3 Discussion

Infectious diseases remain a significant global public health threat. The emergence of SARS-CoV-2 has rapidly evolved into a worldwide health crisis, causing numerous infections and deaths. This pandemic has placed immense strain on healthcare systems and triggered profound social and economic upheaval. As one of the most effective public health interventions against SARS-CoV-2, vaccination has been pivotal in this challenge.

Humanized mice, generated through the engraftment of human tissues and/or expression of human genes in murine models, have become an indispensable tool in vaccine development. In contrast to conventional animal models, humanized murine models offer a more precise simulation of human immune responses, thereby providing invaluable insights into vaccine efficacy and immunogenicity (Han et al., 2024). Particularly when it comes to understanding the pathogenesis and immune processes of human viruses, immunodeficient murine models engrafted with components of the human immune system (HIS) provide unique opportunities (Douam and Ploss, 2018). Hematopoietic stem cell and peripheral blood mononuclear cell (PBMC) transplanted mice represent two of the most prevalent employed immune-humanized mouse systems (Kametani et al., 2019; Martinov et al., 2021; Chen et al., 2022; Kametani et al., 2024). These models are extensively applied in tumor immunotherapy, infectious diseases research and transplant immunology. Preclinical studies with humanized murine models, like NRG (*NOD-Rag2* ^–/–^ *γc* ^–/–^) and HIS mice (Cheng et al., 2018; Gutierrez-Barbosa et al., 2024; Rodríguez et al., 2025), have revealed great potential in infectious disease research. They can effectively induce virus-specific immune cells, reduce viral reservoir levels, and delay viral rebound. Moreover, these models are useful for evaluating viral infections and exploring human genetic and immune regulatory mechanisms, highlighting their versatility and value in translational research (Gómez-Carballa et al., 2023; Gutierrez-Barbosa et al., 2024; Rodríguez et al., 2025).

In 1993, the Orr laboratory developed the first transgenic *HLA-G* murine model to gain a deeper understanding of its regulatory and expression mechanisms (Ajith et al., 2020). In previous studies, hMHC II murine models were utilized to validate genetic polymorphisms identified in human association studies and to explore their functional implications (Romero-Castillo et al., 2024), all of which have laid the foundation for subsequent studies in hMHC murine models. In the present investigation, we employed a hMHC murine model to simulate human post-vaccination immune states against COVID-19. We developed a genetically engineered hMHC murine model by modifying the endogenous *β_2_m* and *H2-Ab1* gene loci in embryonic stem cells, ablating murine MHC systems and introducing human HLA genes to establish HLA-restricted immunity. In this model, mACE2 has low SARS-CoV-2 binding affinity, but HLA-A*02:01 can recognize viral nucleocapsid proteins to trigger immune responses, and spike protein exposure induces hACE2-independent inflammation. These features allow for the dissection of immune mechanisms missed by conventional models. We performed a horizontal comparison of genomic changes in mice infected with SARS-CoV-2 and discovered that the immune response in the hMHC mice was notably more robust than that in the wild-type. This indicates that human-derived MHC triggers a more intense and rapid immune response against the virus, and quickly activates multiple immune-related pathways to inhibit the virus and rapidly return to normal in the later stages of infection (**Figures 2A, B**). Additionally, our data show that the hMHC mice activate genes associated with lipid metabolism and coagulation pathways (**Figure 2B**), suggesting that SARS-CoV-2 infection in the hMHC mice may lead to thrombosis and thrombocytopenia, similar to what has been observed after ChAdOx1 nCoV-19 vaccination (Schultz et al., 2021).

A key observation is that the immune response of hMHC mice is similar to that of K18-hACE2 transgenic mice post-vaccination, predominantly characterized by IFN-γ production and neutrophil activation. Subsequent comparisons with human whole blood RNA-seq data revealed that the transcriptomic data from the hMHC mice showed high reproducibility and comparable performance in both innate and adaptive immune responses, indicating that the hMHC murine model is suitable as a vaccine model. Nevertheless, there are some limitations in our study. Immunodeficient mice with preserved innate immune cell activity might diminish the efficacy of human cell-based drugs. Despite being humanized, the molecular and cellular environments of these murine models differ from those in humans. The human immune system is inherently more intricate, encompassing regulatory T cells, regulatory B cells, and immunosuppressive myeloid cells. These components vary among patients, meaning that the models merely offer a foundation for biological insights and an initial understanding of disease mechanisms (Kametani et al., 2024; Kumagai et al., 2024; Rassomakhina et al., 2024). Moreover, mice can only detect short-term transcriptional changes and are unable to fully mimic the long-term immune changes post-vaccination in humans. Future studies should focus on monitoring the dynamic changes in immune cells and cytokines. In summary, our humanization strategy enables the hMHC murine model to partially simulate the human immune system’s responses to vaccination, providing a distinctive experimental platform for investigating the human immune response. This approach not only aids in revealing the function of immune system in viral infections but also offers a novel perspective for vaccine development and disease treatment research.

## 4 Materials and methods

### 4.1 Mice and ethical approval

Female C57BL/6 mice (6–8 weeks old) were procured from SPF Biotechnology Co., Ltd. (Beijing, China). Age-matched female hMHC mice, *HLA-A2/DR1* (*HLA-A2^+/+^/DR01^+/+^/H-2-β_2_m^-/-^/IAβ^-/-^*) transgenic mice (Pajot et al., 2004), were provided by the State Key Laboratory of Pathogen and Biosecurity. All mice were maintained in specific pathogen-free (SPF) barrier-controlled facilities with ad libitum access to food and water under standardized environmental conditions.

### 4.2 Transgenic detection

Mouse tails from hMHC mice were clipped and lysed in 500 μL tissue lysis solution at 55°C for 3 h. NaCl was added, followed by chilling at -20°C for 15 min. After centrifuging at 13,000 rpm for 20 min, the supernatant was mixed with ethanol, recentrifuged, and the DNA precipitate was dried and resuspended in nuclease-free water. This DNA was stored at 4°C and subjected to PCR amplification targeting HLA-A*02:01, *HLA-DR01α*, *HLA-DR01β*, mouse *β_2_m*, and mouse *IAβ*. The amplification products were used for agarose gel electrophoresis to detect the expression of transgenic sequences in the hMHC mice.

### 4.3 FACS analysis

Mice were euthanized, and their spleens were harvested. The spleens were homogenized using a 70 μm cell strainer, and the cell suspension was collected and transferred to a 15 mL centrifuge tube. The cells were centrifuged at 300 g for 5 min at 4°C. The supernatant was discarded, and the red blood cells were lysed using 3 mL of ACK lysing buffer (Thermo Fisher, A1049201). The lysis reaction was terminated by adding 3 mL of 1640 medium containing 2% FBS. The cells were again centrifuged at 300 g for 5 min at 4°C, and the supernatant was discarded. The cells were resuspended in 2 mL of 1640 medium containing 2% FBS. A total of 10^6^ cells were added to a flow cytometry tube, and the Fc receptors were blocked using a CD16/32 monoclonal antibody (Biolegend, 101302). After incubation, the mouse splenic cells were stained with PE-labeled *HLA-ABC* (Biolegend, 311406) and FITC-labeled *HLA-DR* (Biolegend, 307604). The cells were washed twice and resuspended in PBS. The expression of HLA-A*02:01 and HLA-DRB1*01:01 genes on the surface of mouse splenic lymphocytes was analyzed using a flow cytometer (Guava^®^ easyCyte HT).

### 4.4 Peptide binding assay

Splenic cells were collected from hMHC humanized mice and prepared into a single-cell suspension. The cells were washed with 2 mL of acidic buffer (0.131 M citric acid and 0.061 M sodium phosphate Na_2_HPO_4_, pH 3.3, filtered through 0.22 μm) for 1 min and then neutralized with 12 mL of 1640 medium containing 2% FBS. The cells were washed twice and seeded into a 48-well culture plate at a density of 1 × 10^6^ cells per well. Fluorescently labeled peptides were added to each well for co-incubation at 4°C for 24 h: (FITC)KLWAQCVQL (for HLA-A*02:01), (FITC)LLLDRLNQL (for HLA-A*02:01), (FITC)DDQIGYYRRATRRIR (for HLA-DRB1*01:01), and (FITC)SYYKLGASQRVAGDS (for HLA-DRB1*01:01). The cells were then transferred to flow cytometry tubes and washed twice at 4°C with centrifugation at 300 g for 5 min. Finally, the cells were resuspended in 200 μL of PBS containing 3% FBS and analyzed by flow cytometry to detect and quantify the expression of HLA molecules.

### 4.5 Mouse infection experiment

All mice for virus infection were intranasally inoculated with 1×10^4^ TCID_50_ of SARS-CoV-2 (wild-type strain) after intraperitoneal anesthetization with sodium pentobarbital. Three days post-infection, lung tissues were aseptically harvested from euthanized mice and immediately processed for total RNA extraction (Chomczynski and Sacchi, 1987) using TRIzol reagent (Thermo Fisher Scientific), followed by DNase I treatment to eliminate genomic DNA contamination. The purified RNA was quantified via NanoDrop spectrophotometry (OD260/280 > 1.8) and integrity was verified by agarose gel electrophoresis. Aliquots were stored at -80°C until subsequent analyses, including RT-qPCR-based viral load quantification and RNA sequencing. All infectious experiments were performed following the standard operating procedures of the approved biosafety level-3 facility and were approved by the Institutional Animal Care and Use Committee of the Academy of Military Medical Sciences (Approval ID: IACUC-IME-2021-017).

### 4.6 Determination of viral load by RT-qPCR

Mouse tissues were collected for virus load assessment using RT-qPCR. Briefly, total RNA from TRIzol-lysed tissues was extracted and reverse-transcribed to cDNA using a reverse transcription kit (TaKaRa, RR036A). Subsequently, viral copies targeting the E gene of SARS-CoV-2 were quantified by real-time PCR using the following probe and primers: E sgRNA-F (5′-CGATCTCTTGTAGATCTGTTCTC-3), E sgRNA-R (5′-ATATTGCAGCAGTACGCACACACA-3′), and E sgRNA-P3 (5′-ACACTAGCCATCCTTACTGCGCTTCG-3′).

### 4.7 RNA sequencing processing and analysis

They were divided into two experimental groups: (i) C57BL/6 mice infected with SARS-CoV-2; (ii) hMHC mice infected with SARS-CoV-2. Raw data (raw reads) were processed using Trimmomatic. The reads containing ploy-N and the low-quality reads were removed to obtain the clean reads. Then the clean reads were mapped to reference genome using hisat2. Fragments Per Kilobase of transcript per Million mapped reads (FPKM) value of each gene was calculated using cufflinks, and the read counts of each gene were obtained by htseq-count. Differentially expressed genes (DEGs) were identified using the DESeq(2012) R package functions estimateSizeFactors and nbinomTest. DEGs were defined as genes with a |Log2 (Fold change) | > 1 and an adjusted P value < 0.05.

Related dataset with SARS-CoV-2 vaccine, we acquired DEGs from GSE199750 datasets (https://www.ncbi.nlm.nih.gov/geo).

### 4.8 Venn diagram

The common gene identification among DEGs with different days of variation was generated using R software (v.4.2.2) package “VennDiagram” (v.1.7.3) (Chen and Boutros, 2011) through Hiplot Pro (https://hiplot.com.cn/), a comprehensive web service for biomedical data analysis and visualization.

### 4.9 Heat map and volcano plot

Heat maps and volcano plots were generated using R software (v.4.2.2) package “pheatmap” (v.1.0.12) (Kolde, 2019) through Hiplot Pro (https://hiplot.com.cn/), a comprehensive web service for biomedical data analysis and visualization.

### 4.10 GSEA analysis

Gene sets including (i) IFN-γ, TNF-α/NF-κB signaling, IFN-α, inflammatory response, complement system, and IL-6/JAK/STAT3 signaling pathways were downloaded from the MSigDB database (Liberzon et al., 2011; Liberzon et al., 2015) and (ii) gene sets of up/down-regulated genes of mice were the differentially expressed genes of 6-day hMHC mice versus 0-day hMHC mice described above with the threshold of FDR q value <0.05 and |Log2 (fold change) | > 1. The gene set enrichment P-value, normalized enrichment score (NES) and FDR values reported throughout were calculated with 18204 features (genes) with GSEA4.3.3 (https://www.gsea-msigdb.org/gsea/index.jsp) (Mootha et al., 2003; Subramanian et al., 2005), ran in Signal2Noise mode.

### 4.11 Gene ontology and network analysis

Gene ontology analysis was performed with a web-based tool of Metascape (https://metascape.org) with differentially expressed genes obtained as described above. A pathway with a P value <0.05 was considered a significantly enriched pathway. Top enriched pathways were shown in a bubble plot created by https://www.bioinformatics.com.cn. The interaction network for each significantly enriched pathway and the protein-protein interaction (PPI) network was drawn by Cytoscape, which is also wrapped in Metascape website. In detail, for each given gene list, PPI enrichment analysis has been carried out with the following databases: BioGrid and STRING (physical score > 0.132). To further capture the relationships between the terms, a subset of enriched terms has been selected and rendered as a network plot, where terms with a similarity >0.3 are connected by edges. We select the terms with the best P values from each of the 20 clusters, with the constraint that there are no more than 15 terms per cluster and no more than 250 terms in total. The network is visualized using Cytoscape, where each node represents an enriched term and is colored by its cluster ID. Nodes that share the same cluster ID are typically close to each other. The resultant network contains the subset of proteins that form physical interactions with at least one other member in the differentially expressed gene list. If the network contains between 3 and 500 proteins, the molecular complex detection (MCODE) algorithm has been applied to identify densely connected network components. Pathway and process enrichment analysis has been applied to each MCODE component independently, and the three best scoring terms by P value have been retained as the functional description of the corresponding components.

## Data Availability

All relevant data is contained within the article/or the **Supplementary Material**. The original contributions presented in the study are included in the **Supplementary File 1**, further inquiries can be directed to the corresponding author/s.

## References

[B1] Ajith A. Portik-Dobos V. Horuzsko D. D. Kapoor R. Mulloy L. L. Horuzsko A. (2020). HLA-G and humanized mouse models as a novel therapeutic approach in transplantation. Hum. Immunol. 81, 178–185. doi: 10.1016/j.humimm.2020.02.006, PMID: 32093884 PMC8672853

[B2] Burnap S. A. Ortega-Prieto A. M. Jimenez-Guardeño J. M. Ali H. Takov K. Fish M.. (2023). Cross-linking mass spectrometry uncovers interactions between high-density lipoproteins and the SARS-coV-2 spike glycoprotein. Mol. Cell Proteomics. 22, 100600. doi: 10.1016/j.mcpro.2023.100600, PMID: 37343697 PMC10279469

[B3] Chatzileontiadou D. Szeto C. Jayasinghe D. Gras S. (2021). Protein purification and crystallization of HLA-A∗02:01 in complex with SARS-CoV-2 peptides. STAR Protoc. 2, 100635. doi: 10.1016/j.xpro.2021.100635, PMID: 34124695 PMC8188458

[B4] Chavakis T. Kanse S. M. May A. E. Preissner K. T. (2002). Haemostatic factors occupy new territory: the role of the urokinase receptor system and kininogen in inflammation. Biochem. Soc. Trans. 30, 168–173. doi: 10.1042/bst0300168, PMID: 12023845

[B5] Chen H. Boutros P. C. (2011). VennDiagram: a package for the generation of highly-customizable Venn and Euler diagrams in R. BMC Bioinf. 12, 35. doi: 10.1186/1471-2105-12-35, PMID: 21269502 PMC3041657

[B6] Chen J. Liao S. Xiao Z. Pan Q. Wang X. Shen K.. (2022). The development and improvement of immunodeficient mice and humanized immune system mouse models. Front. Immunol. 13, 1007579. doi: 10.3389/fimmu.2022.1007579, PMID: 36341323 PMC9626807

[B7] Cheng L. Wang Q. Li G. Banga R. Ma J. Yu H.. (2018). TLR3 agonist and CD40-targeting vaccination induces immune responses and reduces HIV-1 reservoirs. J. Clin. Invest. 128, 4387–4396. doi: 10.1172/JCI99005, PMID: 30148455 PMC6159955

[B8] Chomczynski P. Sacchi N. (1987). Single-step method of RNA isolation by acid guanidinium thiocyanate-phenol-chloroform extraction. Anal. Biochem. 162, 156–159. doi: 10.1016/0003-2697(87)90021-2 2440339

[B9] Covassin L. Laning J. Abdi R. Langevin D. L. Phillips N. E. Shultz L. D.. (2011). Human peripheral blood CD4 T cell-engrafted non-obese diabetic-scid IL2rγ(null) H2-Ab1 (tm1Gru) Tg (human leucocyte antigen D-related 4) mice: a mouse model of human allogeneic graft-versus-host disease. Clin. Exp. Immunol. 166, 269–280. doi: 10.1111/j.1365-2249.2011.04462.x, PMID: 21985373 PMC3219902

[B10] Danner R. Chaudhari S. N. Rosenberger J. Surls J. Richie T. L. Brumeanu T. D.. (2011). Expression of HLA class II molecules in humanized NOD.Rag1KO.IL2RgcKO mice is critical for development and function of human T and B cells. PloS One 6, e19826. doi: 10.1371/journal.pone.0019826, PMID: 21611197 PMC3096643

[B11] Dendrou C. A. Petersen J. Rossjohn J. Fugger L. (2018). HLA variation and disease. Nat. Rev. Immunol. 18, 325–339. doi: 10.1038/nri.2017.143, PMID: 29292391

[B12] Douam F. Ploss A. (2018). The use of humanized mice for studies of viral pathogenesis and immunity. Curr. Opin. Virol. 29, 62–71. doi: 10.1016/j.coviro.2018.03.003, PMID: 29604551 PMC5940492

[B13] Gómez-Carballa A. Albericio G. Montoto-Louzao J. Pérez P. Astorgano D. Rivero-Calle I.. (2023). Lung transcriptomics of K18-hACE2 mice highlights mechanisms and genes involved in the MVA-S vaccine-mediated immune response and protection against SARS-CoV-2 infection. Antiviral Res. 220, 105760. doi: 10.1016/j.antiviral.2023.105760, PMID: 37992765

[B14] Gutierrez-Barbosa H. Medina-Moreno S. Perdomo-Celis F. Davis H. Chua J. V. Zapata J. C. (2024). Evaluation of four humanized NOD-derived mouse models for dengue virus-2 infection. Pathogens. 13, 639. doi: 10.3390/pathogens13080639, PMID: 39204240 PMC11357684

[B15] Han R. Su L. Cheng L. (2024). Advancing human vaccine development using humanized mouse models. Vaccines (Basel). 12, 1012. doi: 10.3390/vaccines12091012, PMID: 39340042 PMC11436046

[B16] Jackson C. B. Farzan M. Chen B. Choe H. (2022). Mechanisms of SARS-CoV-2 entry into cells. Nat. Rev. Mol. Cell Biol. 23, 3–20. doi: 10.1038/s41580-021-00418-x, PMID: 34611326 PMC8491763

[B17] Jaiswal S. Pearson T. Friberg H. Shultz L. D. Greiner D. L. Rothman A. L.. (2009). Dengue virus infection and virus-specific HLA-A2 restricted immune responses in humanized NOD-scid IL2rgammanull mice. PloS One 4, e7251. doi: 10.1371/journal.pone.0007251, PMID: 19802382 PMC2749937

[B18] Jones R. D. Taylor A. M. Tong E. Y. Repa J. J. (2013). Carboxylesterases are uniquely expressed among tissues and regulated by nuclear hormone receptors in the mouse. Drug Metab. Dispos. 41, 40–49. doi: 10.1124/dmd.112.048397, PMID: 23011759 PMC3533427

[B19] Kametani Y. Ito R. Manabe Y. Kulski J. K. Seki T. Ishimoto H.. (2024). PBMC-engrafted humanized mice models for evaluating immune-related and anticancer drug delivery systems. Front. Mol. Biosci. 11, 1447315. doi: 10.3389/fmolb.2024.1447315, PMID: 39228913 PMC11368775

[B20] Kametani Y. Ohno Y. Ohshima S. Tsuda B. Yasuda A. Seki T.. (2019). Humanized mice as an effective evaluation system for peptide vaccines and immune checkpoint inhibitors. Int. J. Mol. Sci. 20, 6337. doi: 10.3390/ijms20246337, PMID: 31888191 PMC6940818

[B21] Kolde R. (2019). pheatmap: pretty heatmaps.

[B22] Kumagai S. Itahashi K. Nishikawa H. (2024). Regulatory T cell-mediated immunosuppression orchestrated by cancer: towards an immuno-genomic paradigm for precision medicine. Nat. Rev. Clin. Oncol. 21, 337–353. doi: 10.1038/s41571-024-00870-6, PMID: 38424196

[B23] Le Chevalier F. Authié P. Chardenoux S. Bourgine M. Vesin B. Cussigh D.. (2023). Mice humanized for MHC and hACE2 with high permissiveness to SARS-CoV-2 omicron replication. Microbes Infect. 25, 105142. doi: 10.1016/j.micinf.2023.105142, PMID: 37080384 PMC10113602

[B24] Lee J. Y. Han A. R. Lee D. R. (2019). T lymphocyte development and activation in humanized mouse model. Dev. Reprod. 23, 79–92. doi: 10.12717/DR.2019.23.2.079, PMID: 31321348 PMC6635618

[B25] Liberzon A. Birger C. Thorvaldsdóttir H. Ghandi M. Mesirov J. P. Tamayo P.. (2015). The Molecular Signatures Database (MSigDB) hallmark gene set collection. Cell Syst. 1, 417–425. doi: 10.1016/j.cels.2015.12.004, PMID: 26771021 PMC4707969

[B26] Liberzon A. Subramanian A. Pinchback R. Thorvaldsdóttir H. Tamayo P. Mesirov J. P.. (2011). Molecular signatures database (MSigDB) 3.0. Bioinformatics 27, 1739–1740. doi: 10.1093/bioinformatics/btr260, PMID: 21546393 PMC3106198

[B27] Martinov T. McKenna K. M. Tan W. H. Collins E. J. Kehret A. R. Linton J. D.. (2021). Building the next generation of humanized hemato-lymphoid system mice. Front. Immunol. 12, 643852. doi: 10.3389/fimmu.2021.643852, PMID: 33692812 PMC7938325

[B28] Mootha V. K. Lindgren C. M. Eriksson K. F. Subramanian A. Sihag S. Lehar J.. (2003). PGC-1alpha-responsive genes involved in oxidative phosphorylation are coordinately downregulated in human diabetes. Nat. Genet. 34, 267–273. doi: 10.1038/ng1180, PMID: 12808457

[B29] Pajot A. Michel M. L. Fazilleau N. Pancré V. Auriault C. Ojcius D. M.. (2004). A mouse model of human adaptive immune functions: HLA-A2.1-/HLA-DR1-transgenic H-2 class I-/class II-knockout mice. Eur. J. Immunol. 34, 3060–3069. doi: 10.1002/eji.200425463, PMID: 15468058

[B30] Pintanel-Raymundo M. Menao-Guillén S. Perales-Afán J. J. García-Gutiérrez A. Moreno-Gázquez I. Julián-Ansón M.. (2024). Analysis of the expression of the Serpina1 gene in SARS-CoV-2 infection: study of a new biomarker. Rev. Clin. Esp (Barc). 224, 253–258. doi: 10.1016/j.rce.2024.03.002, PMID: 38608729

[B31] Rassomakhina N. V. Ryazanova A. Y. Likhov A. R. Bruskin S. A. Maloshenok L. G. Zherdeva V. V. (2024). Tumor organoids: the era of personalized medicine. Biochem. (Mosc). 89, S127–S147. doi: 10.1134/S0006297924140086, PMID: 38621748

[B32] Reipert B. M. Steinitz K. N. van Helden P. M. Unterthurner S. Schuster M. Ahmad R. U.. (2009). Opportunities and limitations of mouse models humanized for HLA class II antigens. J. Thromb. Haemost. 7 Suppl 1, 92–97. doi: 10.1111/j.1538-7836.2009.03403.x, PMID: 19630777

[B33] Rodríguez E. Muñoz-Fontela C. Escudero-Pérez B. (2025). Filovirus infection in humanized mouse models. Methods Mol. Biol. 2877, 213–226. doi: 10.1007/978-1-0716-4256-6_15, PMID: 39585624

[B34] Romero-Castillo L. Li T. Do N. N. Sareila O. Xu B. Hennings V.. (2024). Human MHC class II and invariant chain knock-in mice mimic rheumatoid arthritis with allele restriction in immune response and arthritis association. Adv. Sci. (Weinh). 11, e2401513. doi: 10.1002/advs.202401513, PMID: 38602454 PMC11187888

[B35] Ryan F. J. Norton T. S. McCafferty C. Blake S. J. Stevens N. E. James J.. (2023). A systems immunology study comparing innate and adaptive immune responses in adults to COVID-19 mRNA and adenovirus vectored vaccines. Cell Rep. Med. 4, 100971. doi: 10.1016/j.xcrm.2023.100971, PMID: 36871558 PMC9935276

[B36] Santos-Rebouças C. B. Ferreira C. Nogueira J. S. Brustolini O. J. de Almeida L. Gerber A. L.. (2024). Immune response stability to the SARS-CoV-2 mRNA vaccine booster is influenced by differential splicing of HLA genes. Sci. Rep. 14, 8982. doi: 10.1038/s41598-024-59259-1, PMID: 38637586 PMC11026523

[B37] Schultz N. H. Sørvoll I. H. Michelsen A. E. Munthe L. A. Lund-Johansen F. Ahlen M. T.. (2021). Thrombosis and Thrombocytopenia after ChAdOx1 nCoV-19 Vaccination. N Engl. J. Med. 384, 2124–2130. doi: 10.1056/NEJMoa2104882, PMID: 33835768 PMC8112568

[B38] Shultz L. D. Saito Y. Najima Y. Tanaka S. Ochi T. Tomizawa M.. (2010). Generation of functional human T-cell subsets with HLA-restricted immune responses in HLA class I expressing NOD/SCID/IL2r gamma(null) humanized mice. Proc. Natl. Acad. Sci. U S A. 107, 13022–13027. doi: 10.1073/pnas.1000475107, PMID: 20615947 PMC2919921

[B39] Strowig T. Gurer C. Ploss A. Liu Y. F. Arrey F. Sashihara J.. (2009). Priming of protective T cell responses against virus-induced tumors in mice with human immune system components. J. Exp. Med. 206, 1423–1434. doi: 10.1084/jem.20081720, PMID: 19487422 PMC2715061

[B40] Subramanian A. Tamayo P. Mootha V. K. Mukherjee S. Ebert B. L. Gillette M. A.. (2005). Gene set enrichment analysis: a knowledge-based approach for interpreting genome-wide expression profiles. Proc. Natl. Acad. Sci. U.S.A. 102, 15545–15550. doi: 10.1073/pnas.0506580102, PMID: 16199517 PMC1239896

[B41] Sun W. Shi J. Wu J. Zhang J. Chen H. Li Y.. (2018). A modified HLA-A*0201-restricted CTL epitope from human oncoprotein (hPEBP4) induces more efficient antitumor responses. Cell Mol. Immunol. 15, 768–781. doi: 10.1038/cmi.2017.155, PMID: 29375131 PMC6141579

[B42] Sun J. Zhuang Z. Zheng J. Li K. Wong R. L. Liu D.. (2020). Generation of a broadly useful model for COVID-19 pathogenesis, vaccination, and treatment. Cell. 182, 734–743.e5. doi: 10.1016/j.cell.2020.06.010, PMID: 32643603 PMC7284240

[B43] Umar S. Palasiewicz K. Meyer A. Kumar P. Prabhakar B. S. Volin M. V.. (2022). Inhibition of IRAK4 dysregulates SARS-CoV-2 spike protein-induced macrophage inflammatory and glycolytic reprogramming. Cell Mol. Life Sci. 79, 301. doi: 10.1007/s00018-022-04329-8, PMID: 35588018 PMC9118817

[B44] Wang X. Sanborn M. A. Dai Y. Rehman J. (2022). Temporal transcriptomic analysis using TrendCatcher identifies early and persistent neutrophil activation in severe COVID-19. JCI Insight 7, e157255. doi: 10.1172/jci.insight.157255, PMID: 35175937 PMC9057597

[B45] Xia J. Tang W. Wang J. Lai D. Xu Q. Huang R.. (2021). SARS-coV-2 N protein induces acute lung injury in mice via NF-ĸB activation. Front. Immunol. 12, 791753. doi: 10.3389/fimmu.2021.791753, PMID: 34950152 PMC8688532

