# Supplementary material for: Transcriptomic profiling reveals SARS-CoV-2-infected humanized MHC mice recapitulate human post vaccination immune responses
Source: Front Cell Infect Microbiol. 2025 Aug 1;15:1634577. doi: 10.3389/fcimb.2025.1634577 (PMC12353716; doi:10.3389/fcimb.2025.1634577)
Supplement: Supplementary file 1 [file Table1.docx]

Supplementary Material

# Supplementary Figures


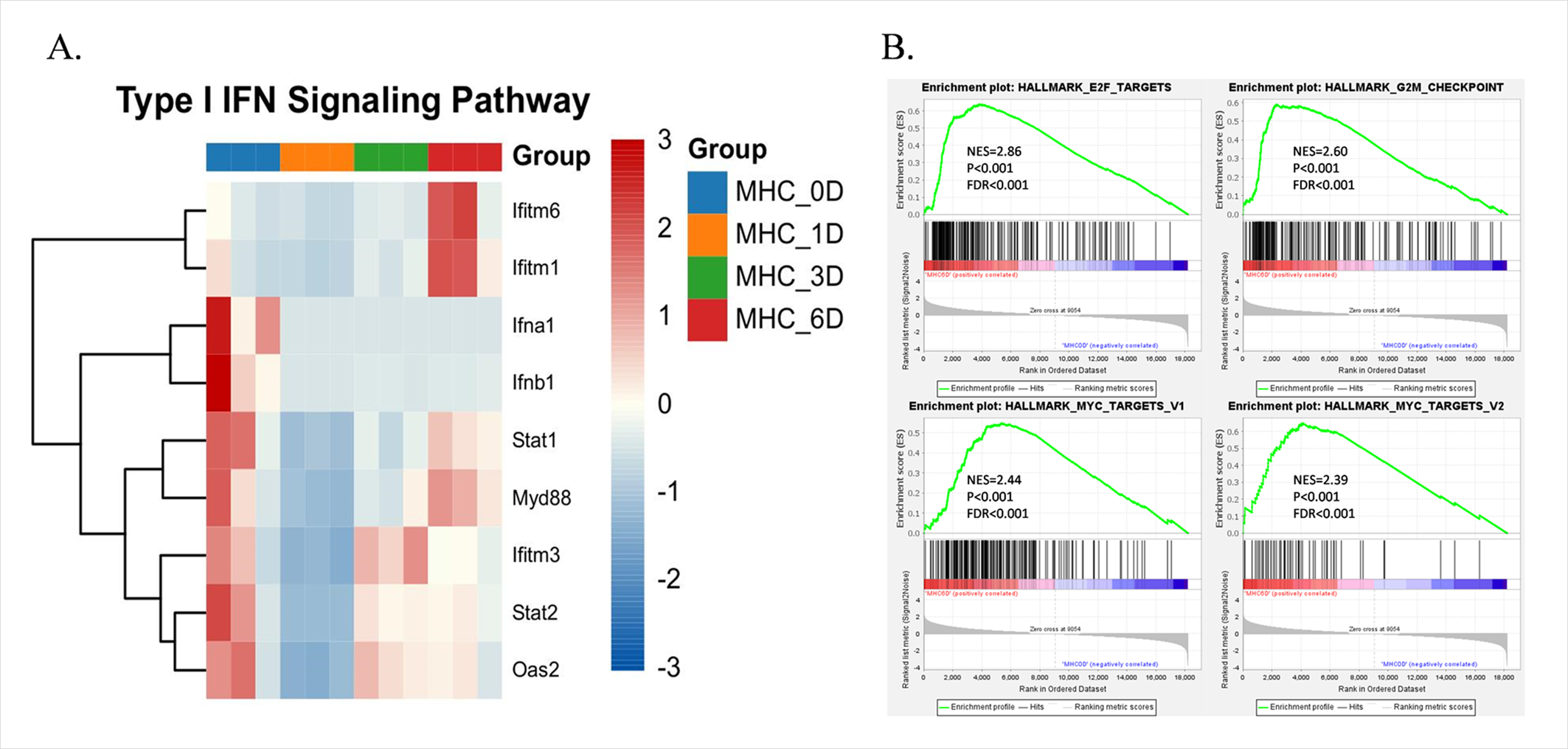


**Supplementary Figure 1.** **(A)** Heatmap of Type Ⅰ IFN signaling pathways in the hMHC mice on various days post-infection; **(B)** GSEA of highly enriched pathways in the hMHC mice on 6-day, with the database downloaded from MSigDB.
