# Supplementary figures and images for: Transcriptomic profiling reveals SARS-CoV-2-infected humanized MHC mice recapitulate human post vaccination immune responses
Source: Front Cell Infect Microbiol. 2025 Aug 1;15:1634577. doi: 10.3389/fcimb.2025.1634577 (PMC12353716; doi:10.3389/fcimb.2025.1634577)

# MHC\_0D-vs-WT\_0D:pValue<0.05&& |log2FC|>1

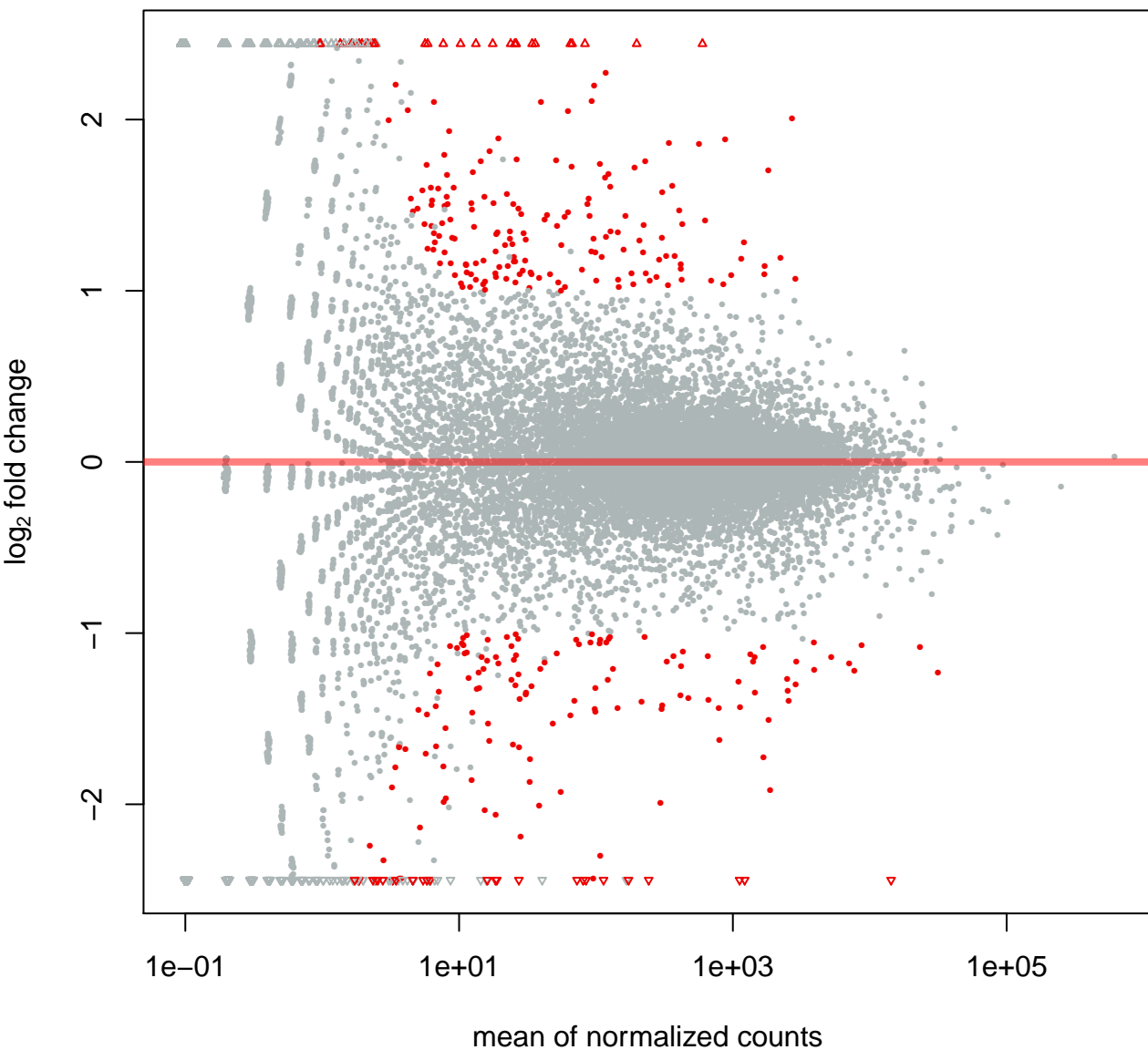

Supplement: Supplementary file 2 [file SupplementaryFile1.zip › Supplementary file 1/original RNAseq data/1.1.different_expressed_gene/MHC_0D-vs-WT_0D-MA-pval-0.05-FC-2.gene.pdf]

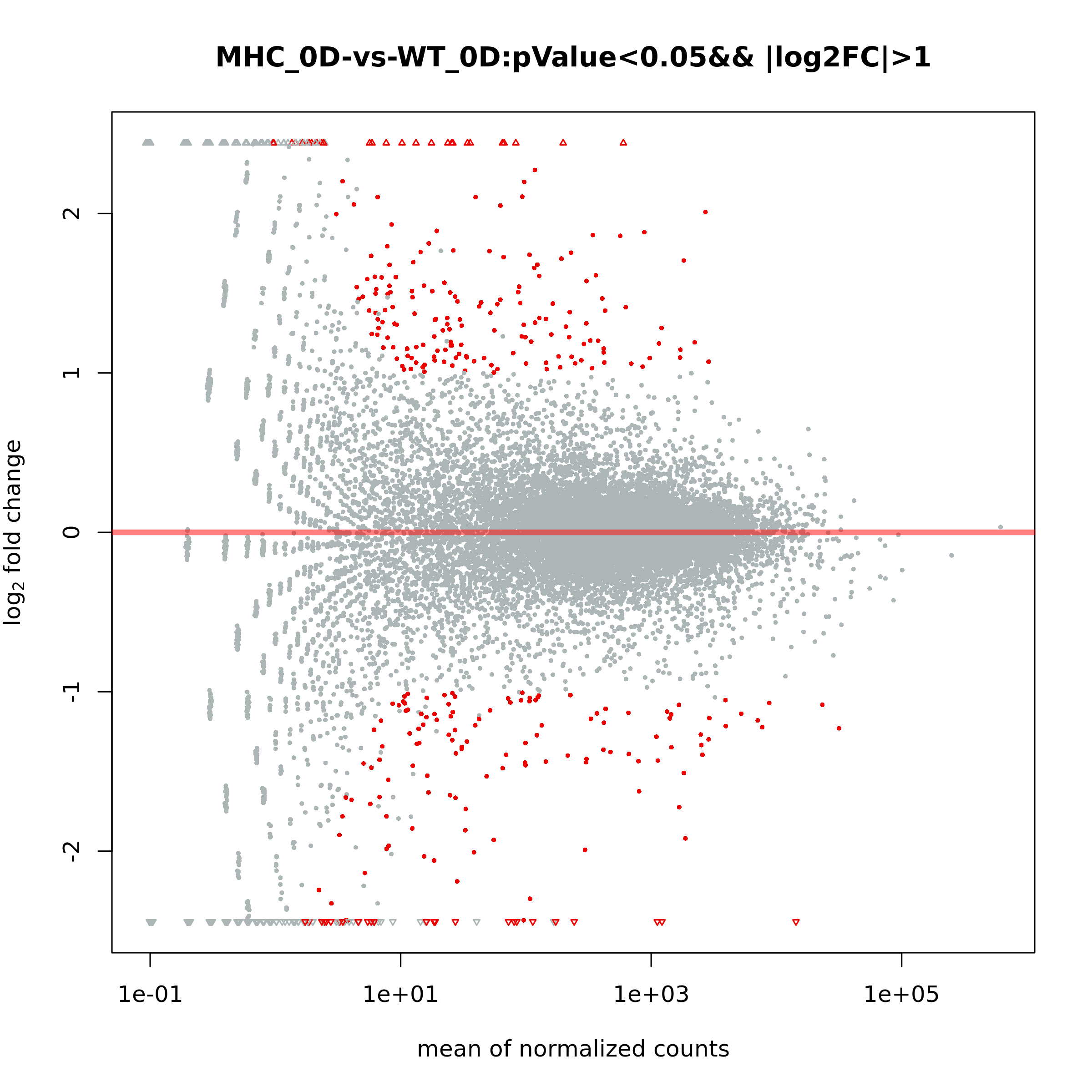

Supplement: Supplementary file 2 [file SupplementaryFile1.zip › Supplementary file 1/original RNAseq data/1.1.different_expressed_gene/MHC_0D-vs-WT_0D-MA-pval-0.05-FC-2.gene.png]

MHC\_0D-vs-WT\_0D:pValue<0.05&& |log2FC|>1

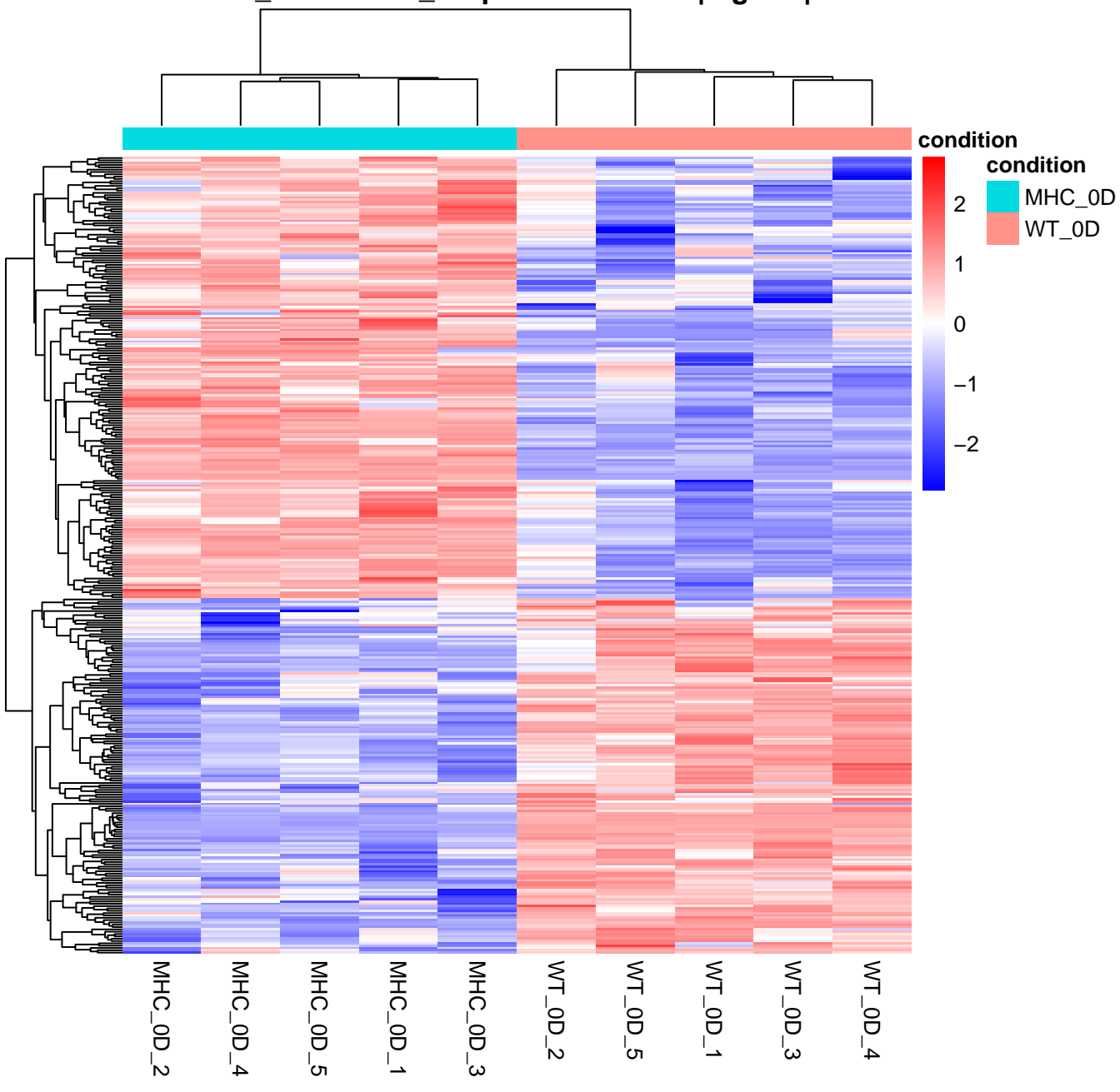

Supplement: Supplementary file 2 [file SupplementaryFile1.zip › Supplementary file 1/original RNAseq data/1.1.different_expressed_gene/MHC_0D-vs-WT_0D-heatmap-pval-0.05-FC-2.gene.pdf]

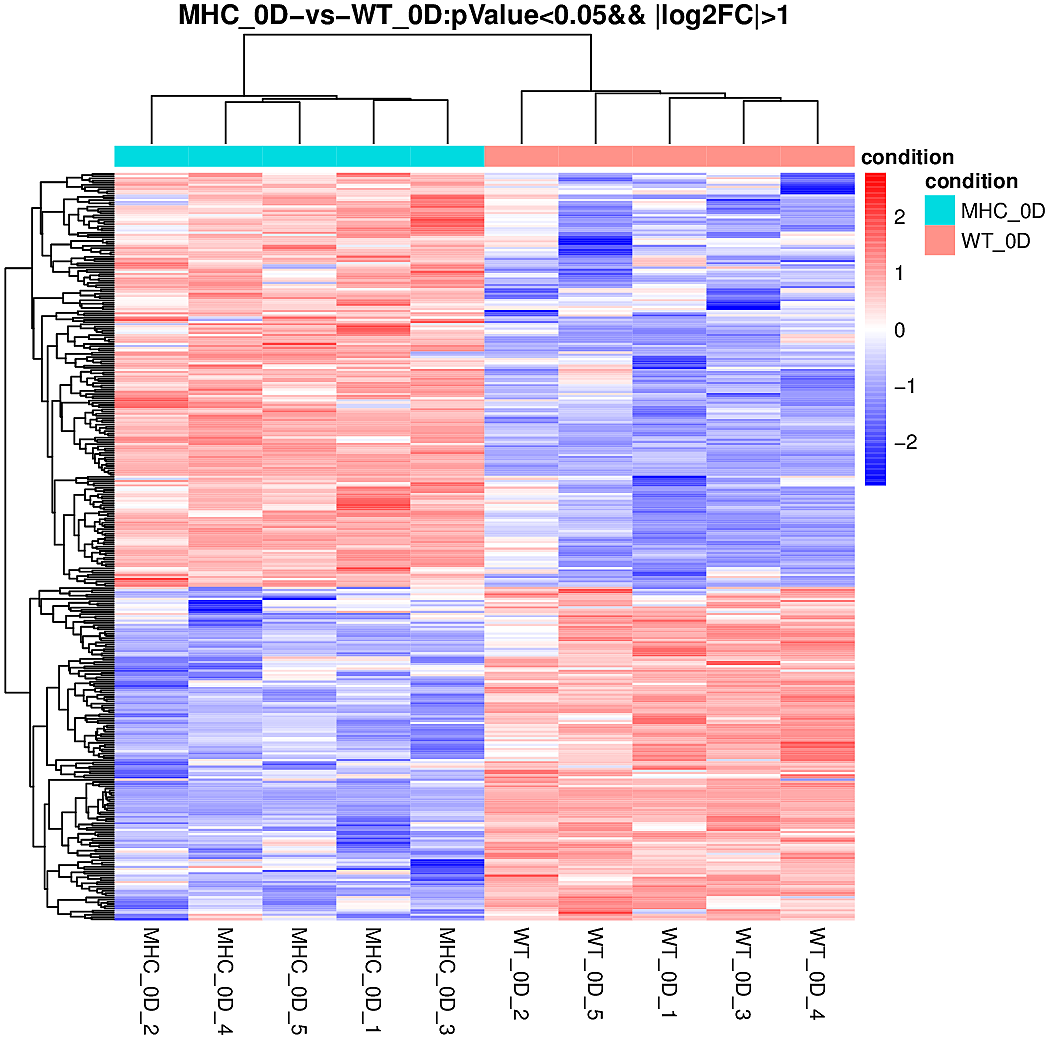

Supplement: Supplementary file 2 [file SupplementaryFile1.zip › Supplementary file 1/original RNAseq data/1.1.different_expressed_gene/MHC_0D-vs-WT_0D-heatmap-pval-0.05-FC-2.gene.png]

# MHC\_0D -vs- WT\_0D : pValue < 0.05 && |log2FC|> 1

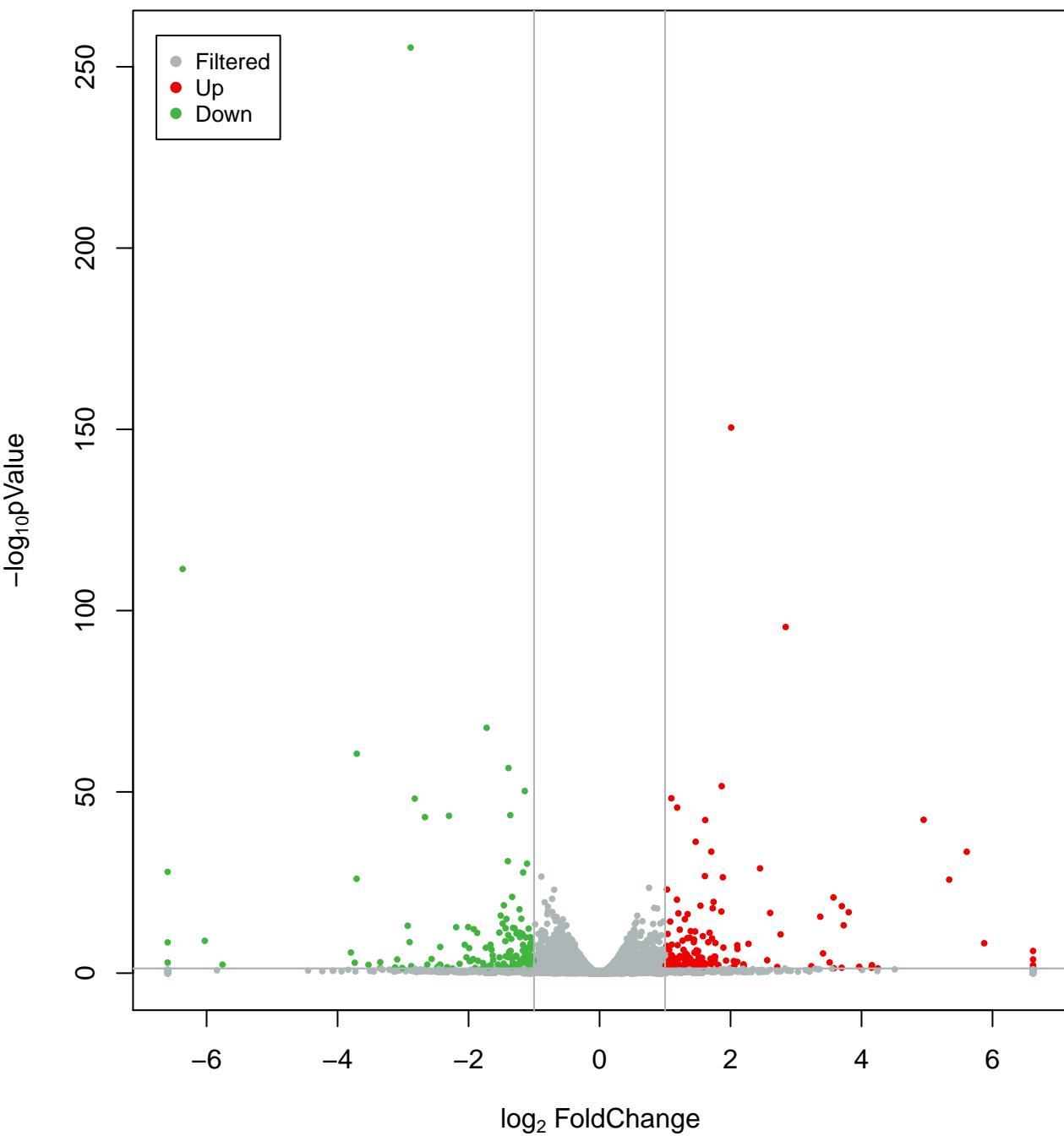

Supplement: Supplementary file 2 [file SupplementaryFile1.zip › Supplementary file 1/original RNAseq data/1.1.different_expressed_gene/MHC_0D-vs-WT_0D-volcano-pval-0.05-FC-2.gene.pdf]

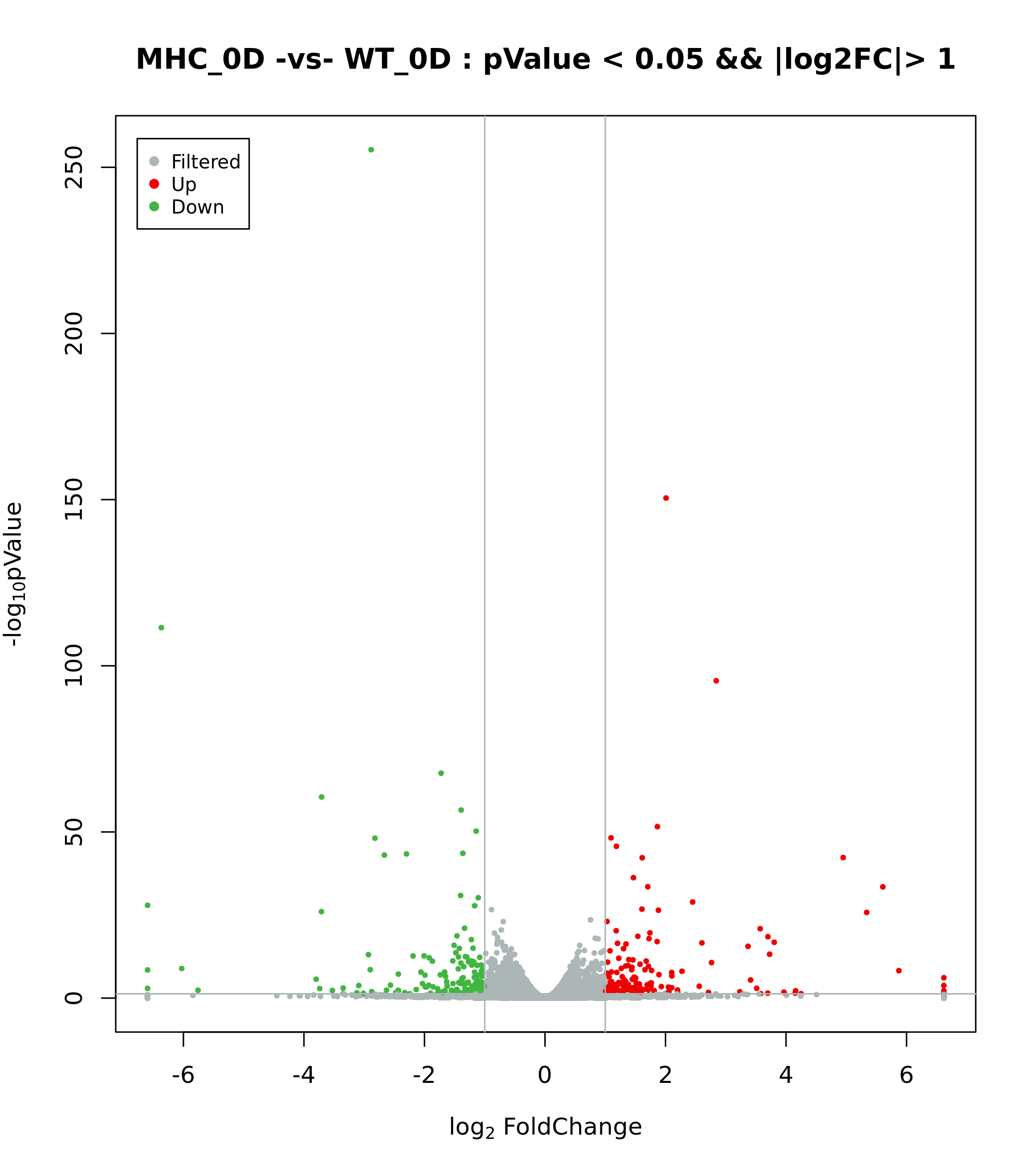

Supplement: Supplementary file 2 [file SupplementaryFile1.zip › Supplementary file 1/original RNAseq data/1.1.different_expressed_gene/MHC_0D-vs-WT_0D-volcano-pval-0.05-FC-2.gene.png]

# MHC\_1D-vs-MHC\_0D:pValue<0.05&& |log2FC|>1

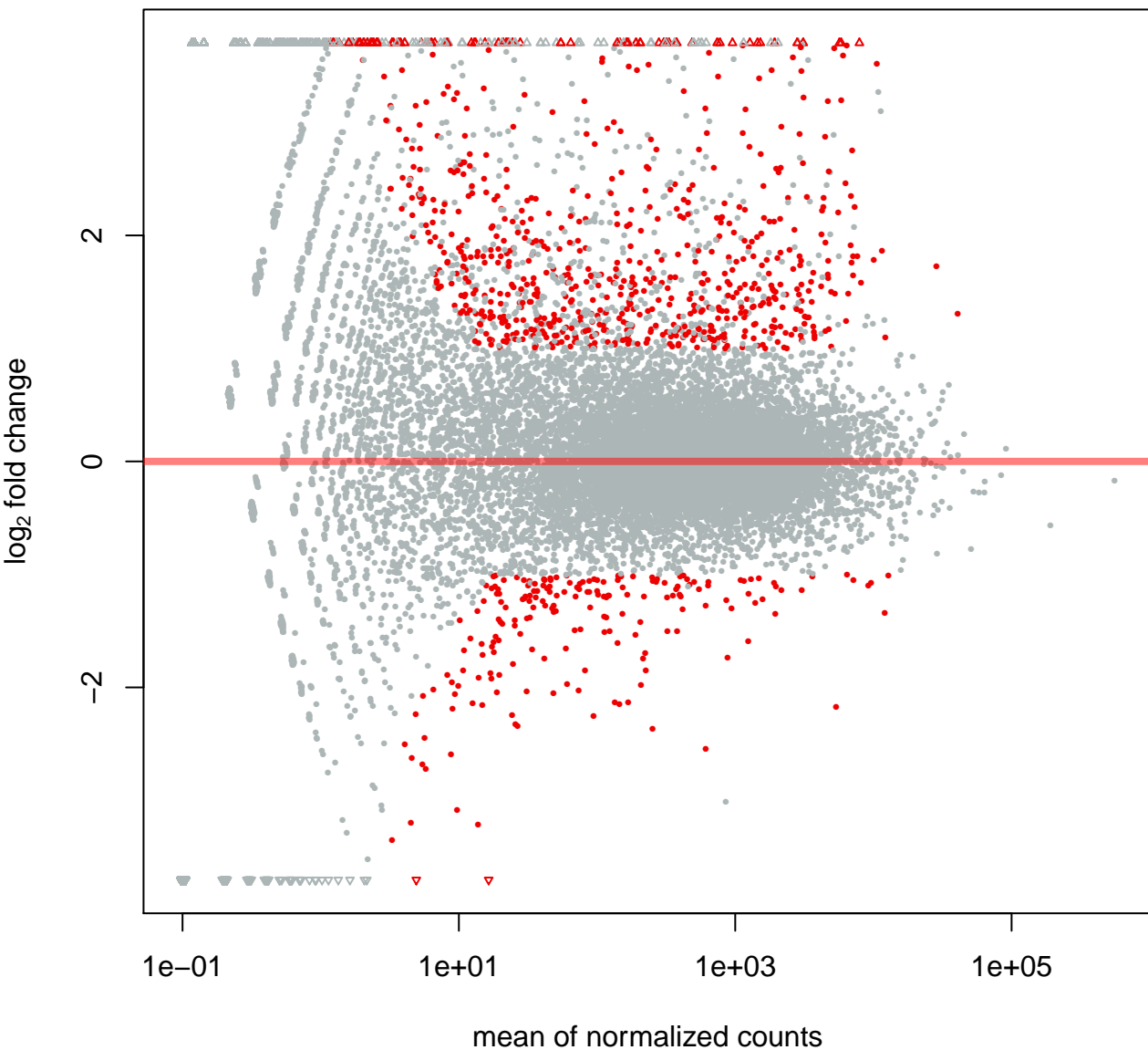

Supplement: Supplementary file 2 [file SupplementaryFile1.zip › Supplementary file 1/original RNAseq data/1.1.different_expressed_gene/MHC_1D-vs-MHC_0D-MA-pval-0.05-FC-2.gene.pdf]

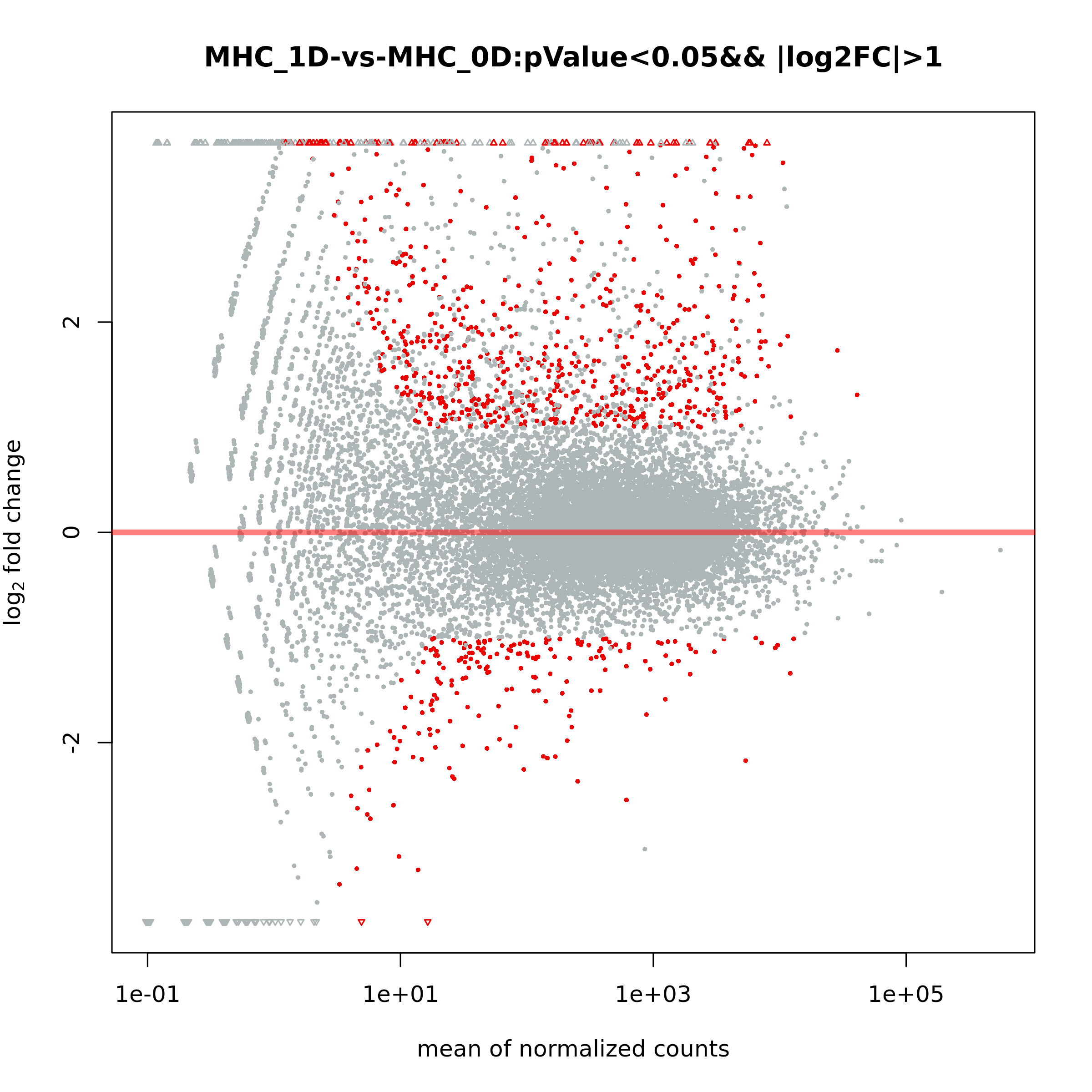

Supplement: Supplementary file 2 [file SupplementaryFile1.zip › Supplementary file 1/original RNAseq data/1.1.different_expressed_gene/MHC_1D-vs-MHC_0D-MA-pval-0.05-FC-2.gene.png]

MHC\_1D-vs-MHC\_0D:pValue<0.05&& |log2FC|>1

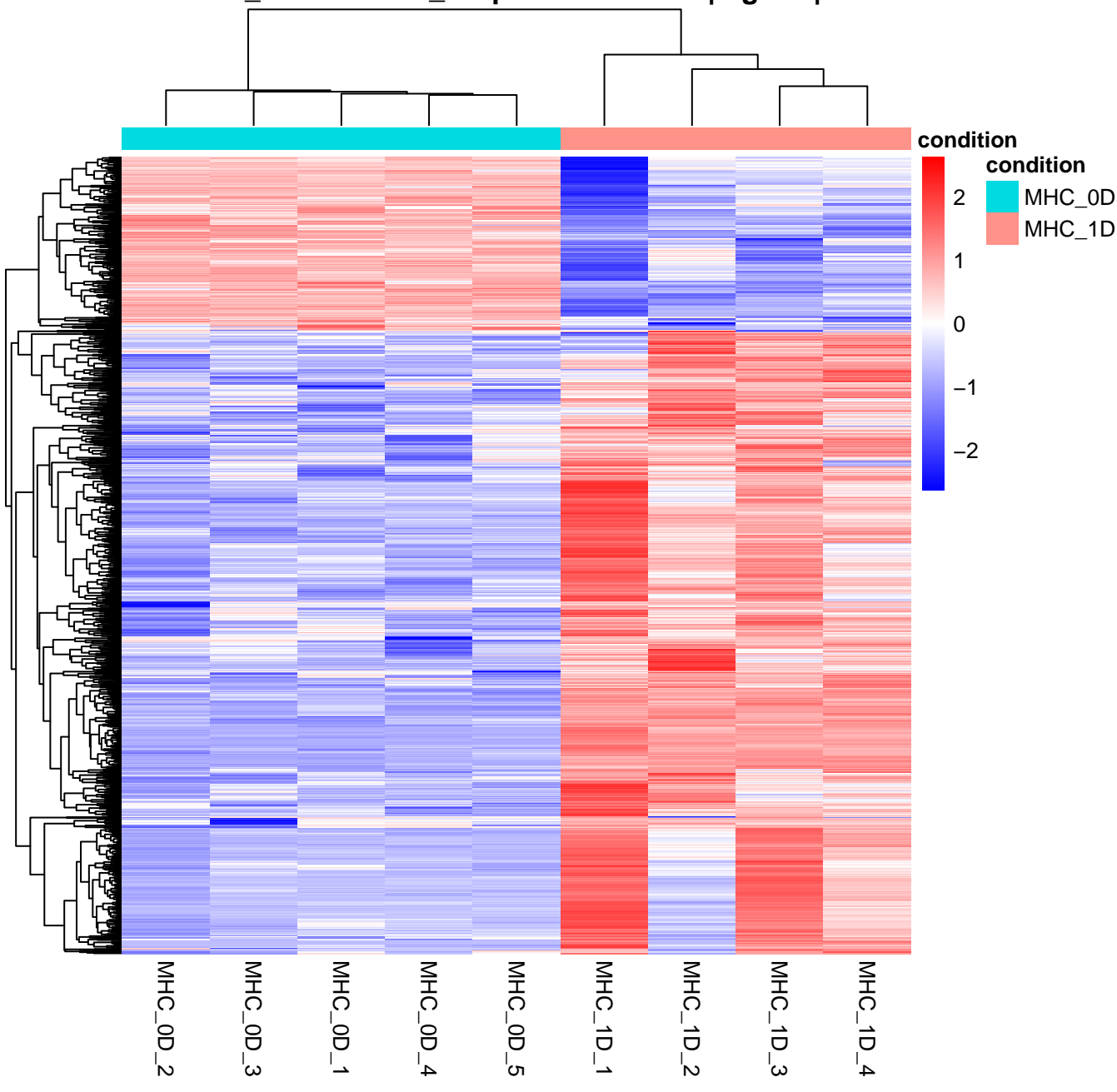

Supplement: Supplementary file 2 [file SupplementaryFile1.zip › Supplementary file 1/original RNAseq data/1.1.different_expressed_gene/MHC_1D-vs-MHC_0D-heatmap-pval-0.05-FC-2.gene.pdf]

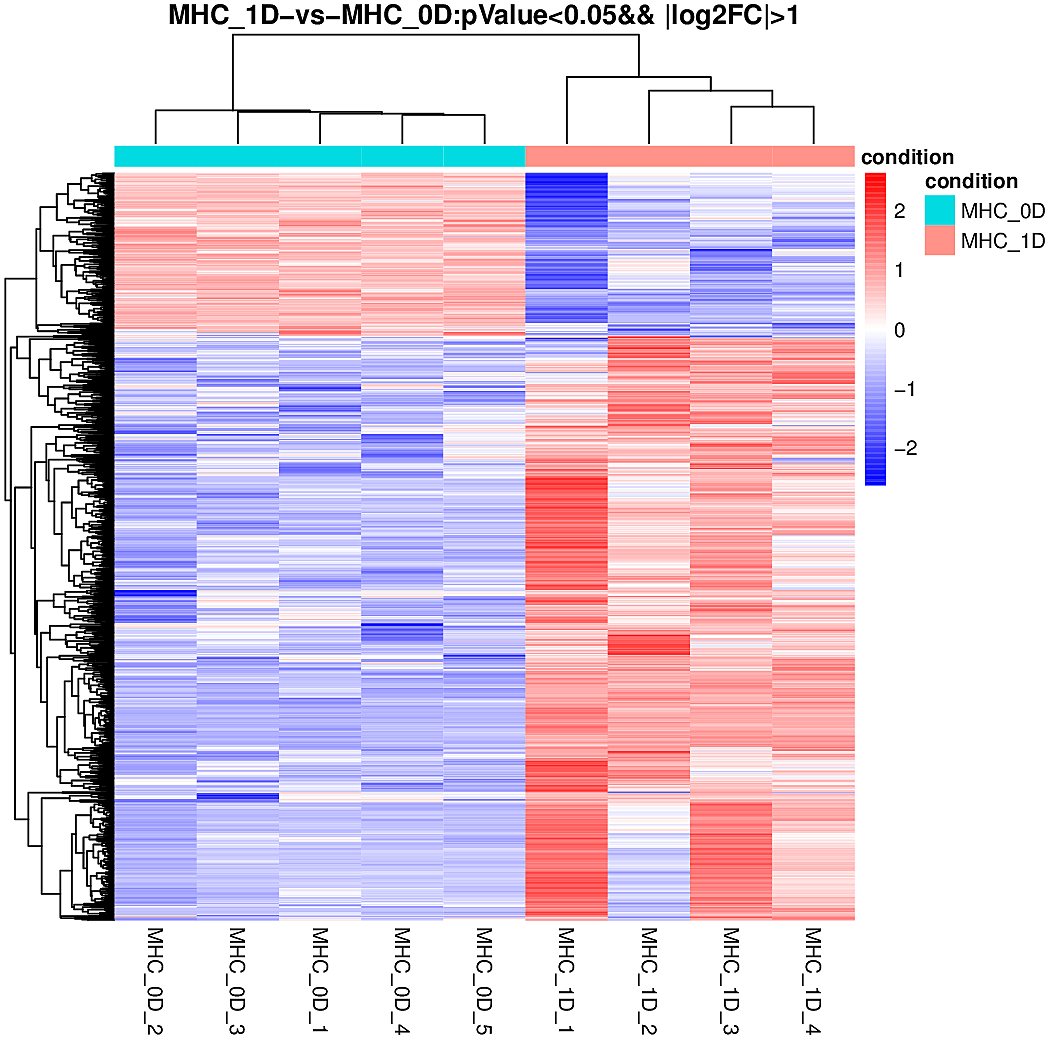

Supplement: Supplementary file 2 [file SupplementaryFile1.zip › Supplementary file 1/original RNAseq data/1.1.different_expressed_gene/MHC_1D-vs-MHC_0D-heatmap-pval-0.05-FC-2.gene.png]

# MHC\_1D -vs- MHC\_0D : pValue < 0.05 && |log2FC|> 1

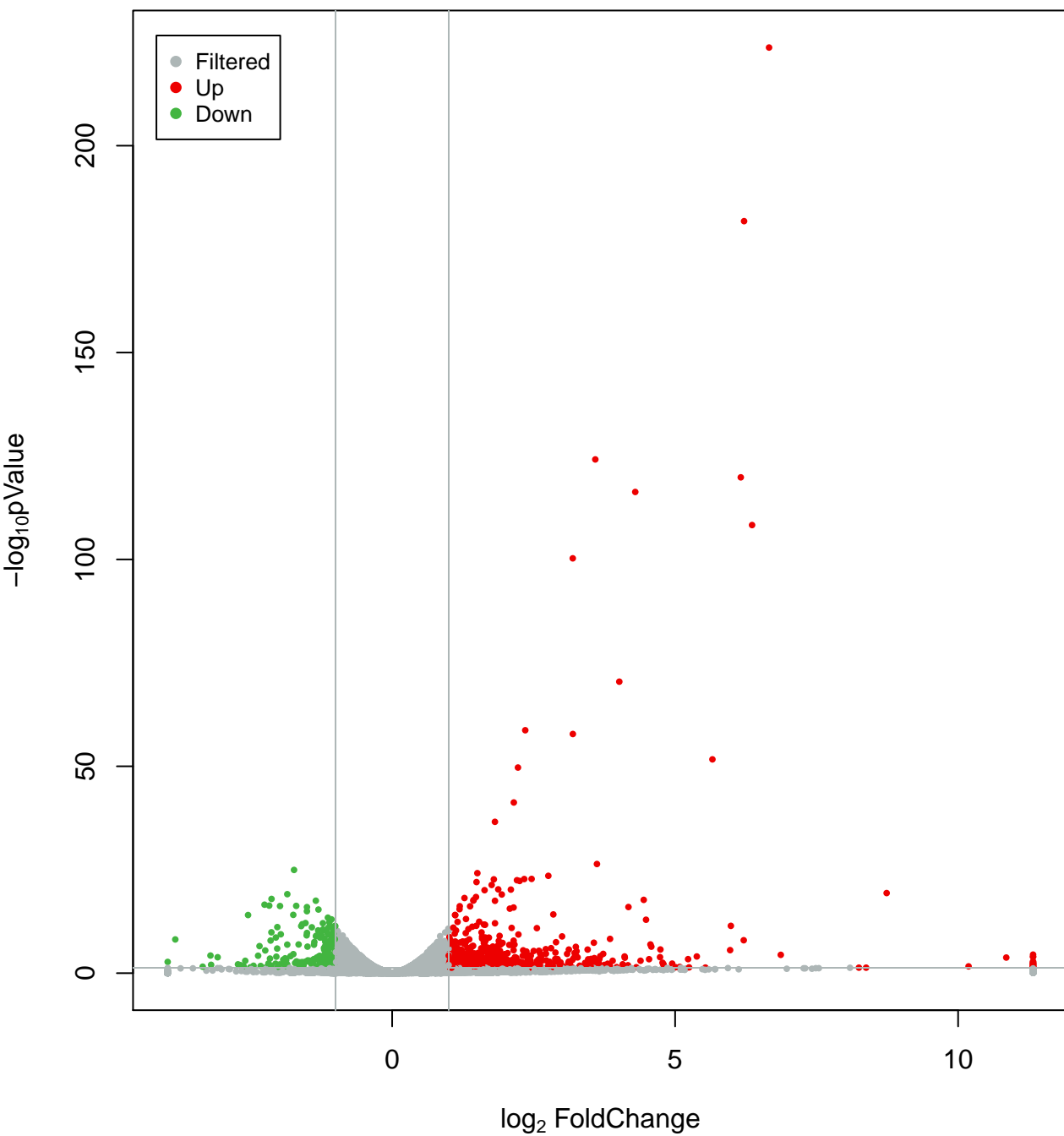

Supplement: Supplementary file 2 [file SupplementaryFile1.zip › Supplementary file 1/original RNAseq data/1.1.different_expressed_gene/MHC_1D-vs-MHC_0D-volcano-pval-0.05-FC-2.gene.pdf]

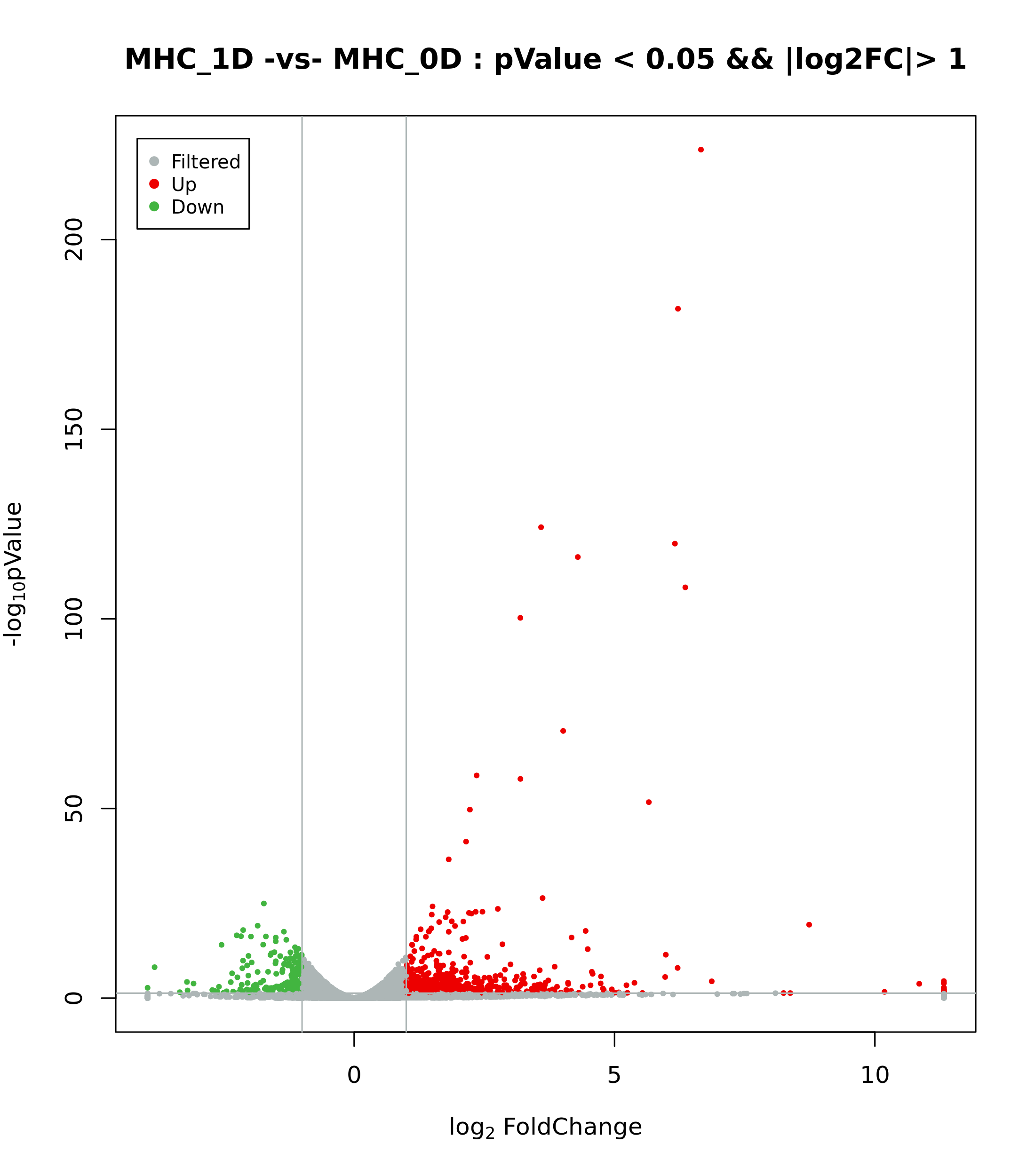

Supplement: Supplementary file 2 [file SupplementaryFile1.zip › Supplementary file 1/original RNAseq data/1.1.different_expressed_gene/MHC_1D-vs-MHC_0D-volcano-pval-0.05-FC-2.gene.png]

# MHC\_1D-vs-WT\_1D:pValue<0.05&& |log2FC|>1

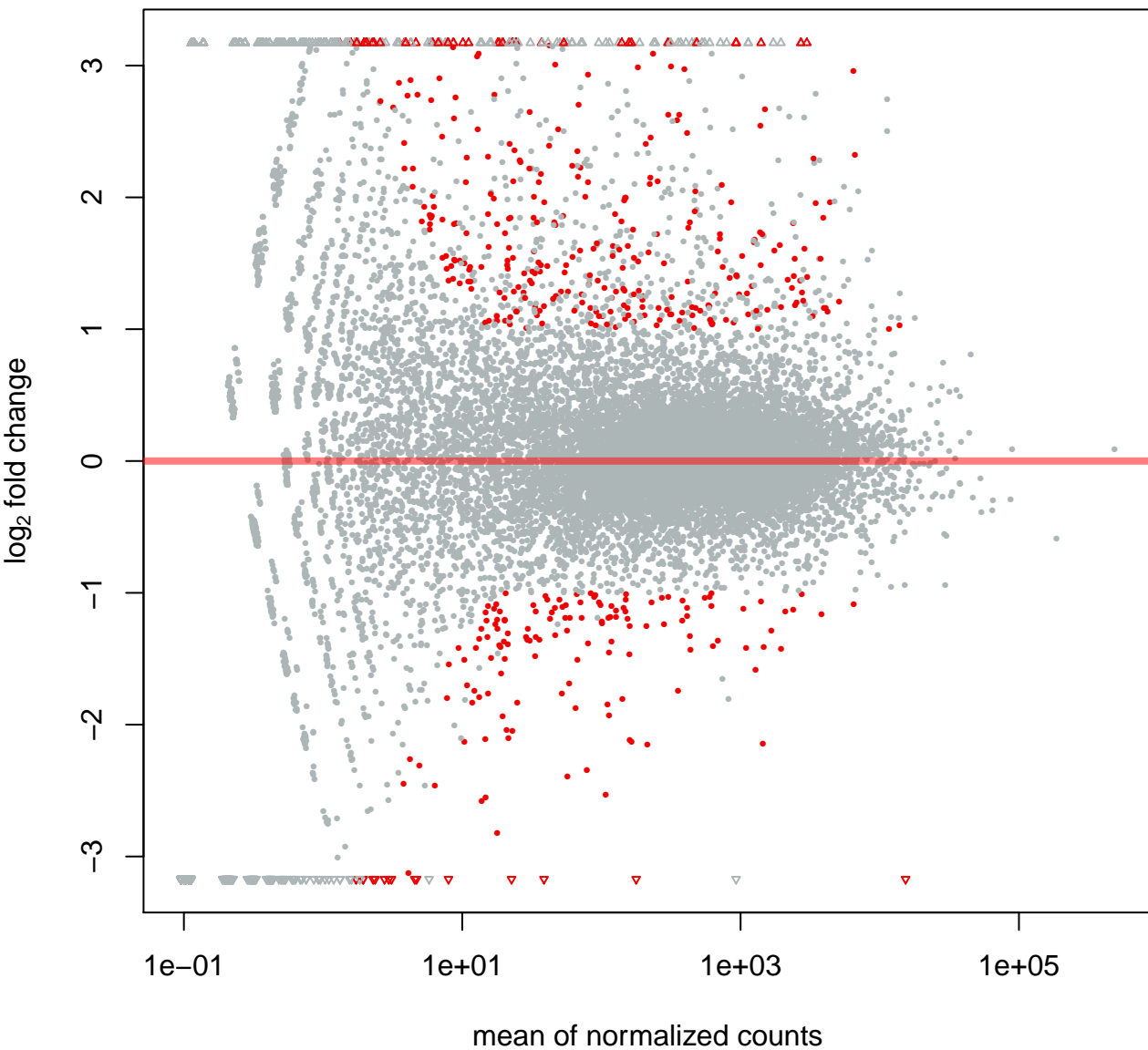

Supplement: Supplementary file 2 [file SupplementaryFile1.zip › Supplementary file 1/original RNAseq data/1.1.different_expressed_gene/MHC_1D-vs-WT_1D-MA-pval-0.05-FC-2.gene.pdf]

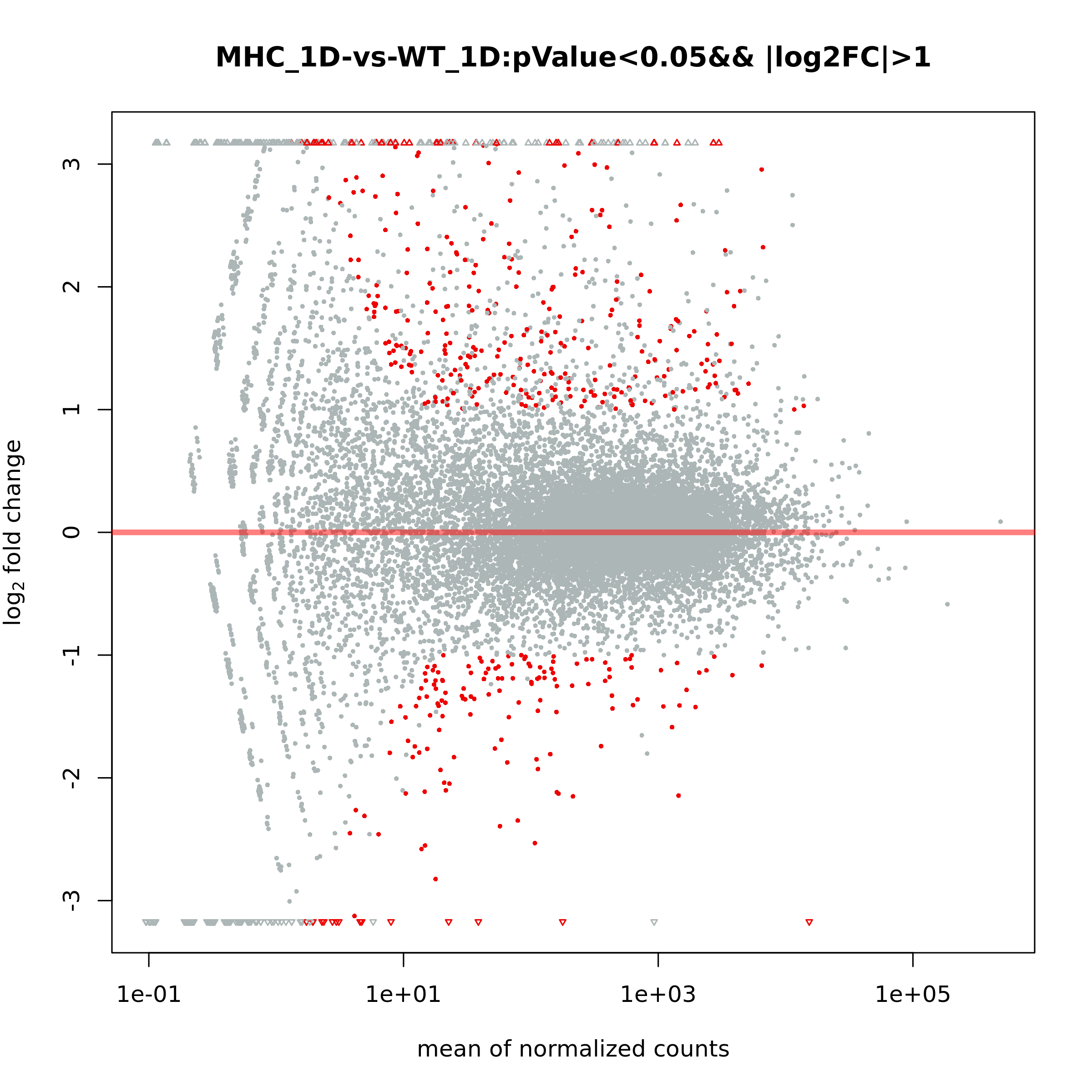

Supplement: Supplementary file 2 [file SupplementaryFile1.zip › Supplementary file 1/original RNAseq data/1.1.different_expressed_gene/MHC_1D-vs-WT_1D-MA-pval-0.05-FC-2.gene.png]

MHC\_1D-vs-WT\_1D:pValue<0.05&& |log2FC|>1

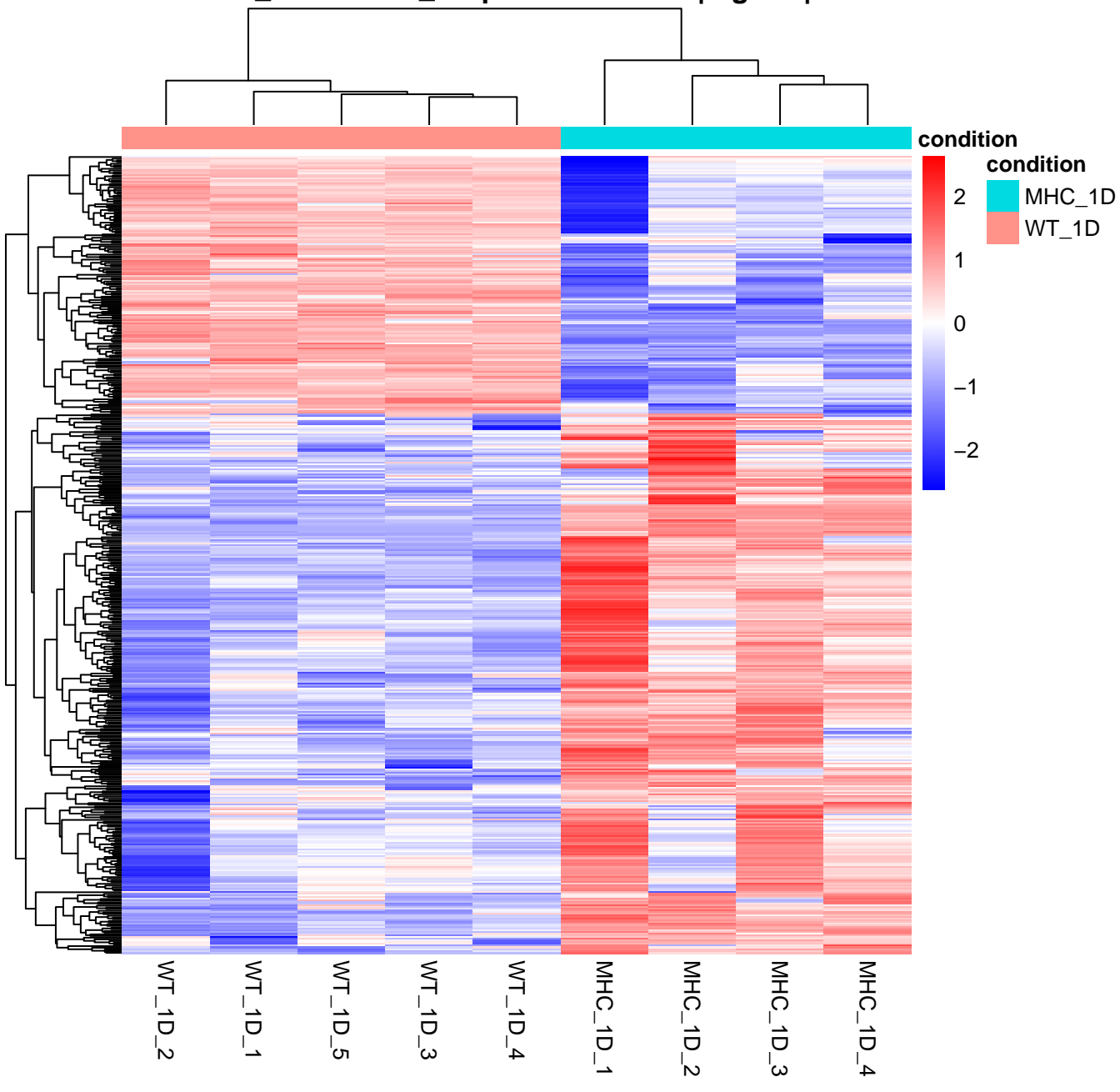

Supplement: Supplementary file 2 [file SupplementaryFile1.zip › Supplementary file 1/original RNAseq data/1.1.different_expressed_gene/MHC_1D-vs-WT_1D-heatmap-pval-0.05-FC-2.gene.pdf]

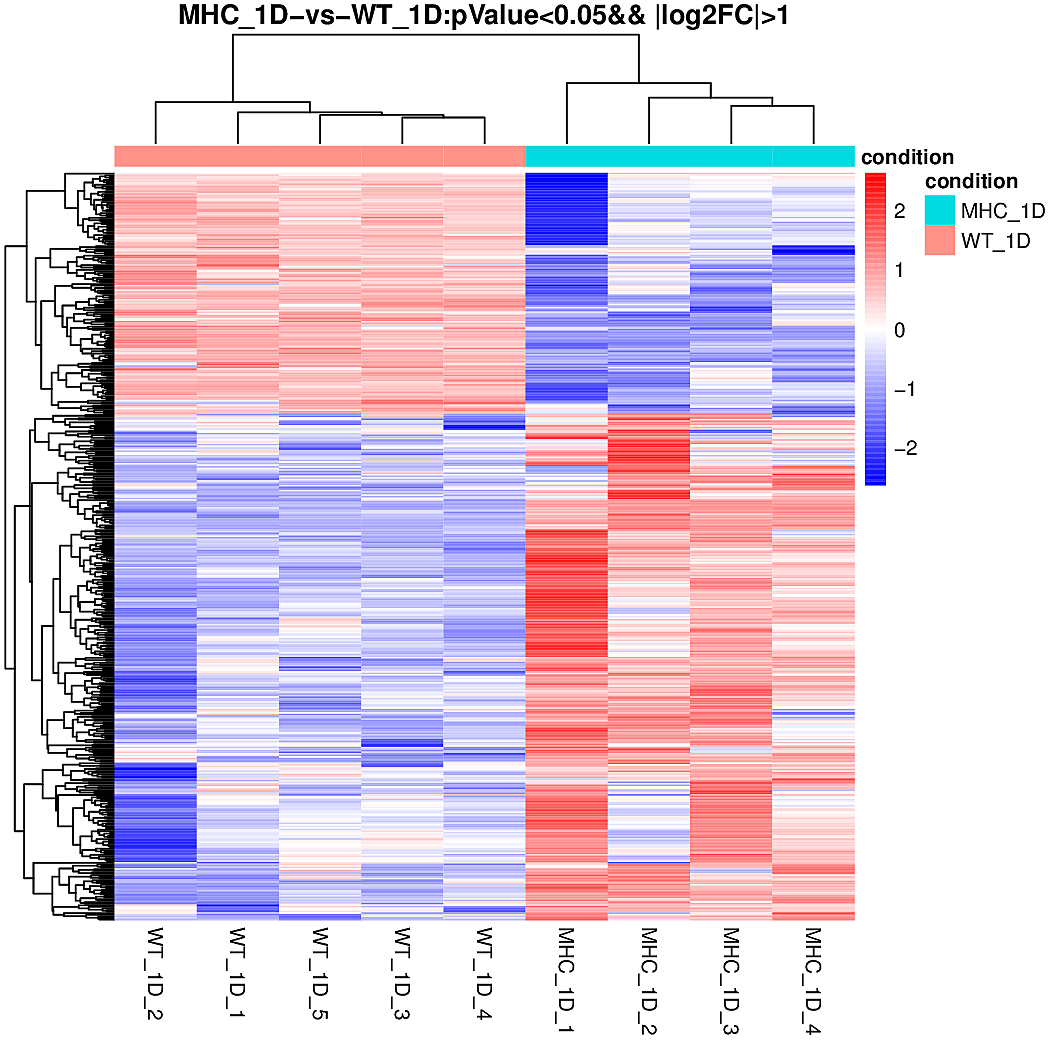

Supplement: Supplementary file 2 [file SupplementaryFile1.zip › Supplementary file 1/original RNAseq data/1.1.different_expressed_gene/MHC_1D-vs-WT_1D-heatmap-pval-0.05-FC-2.gene.png]

# MHC\_1D -vs- WT\_1D : pValue < 0.05 && |log2FC|> 1

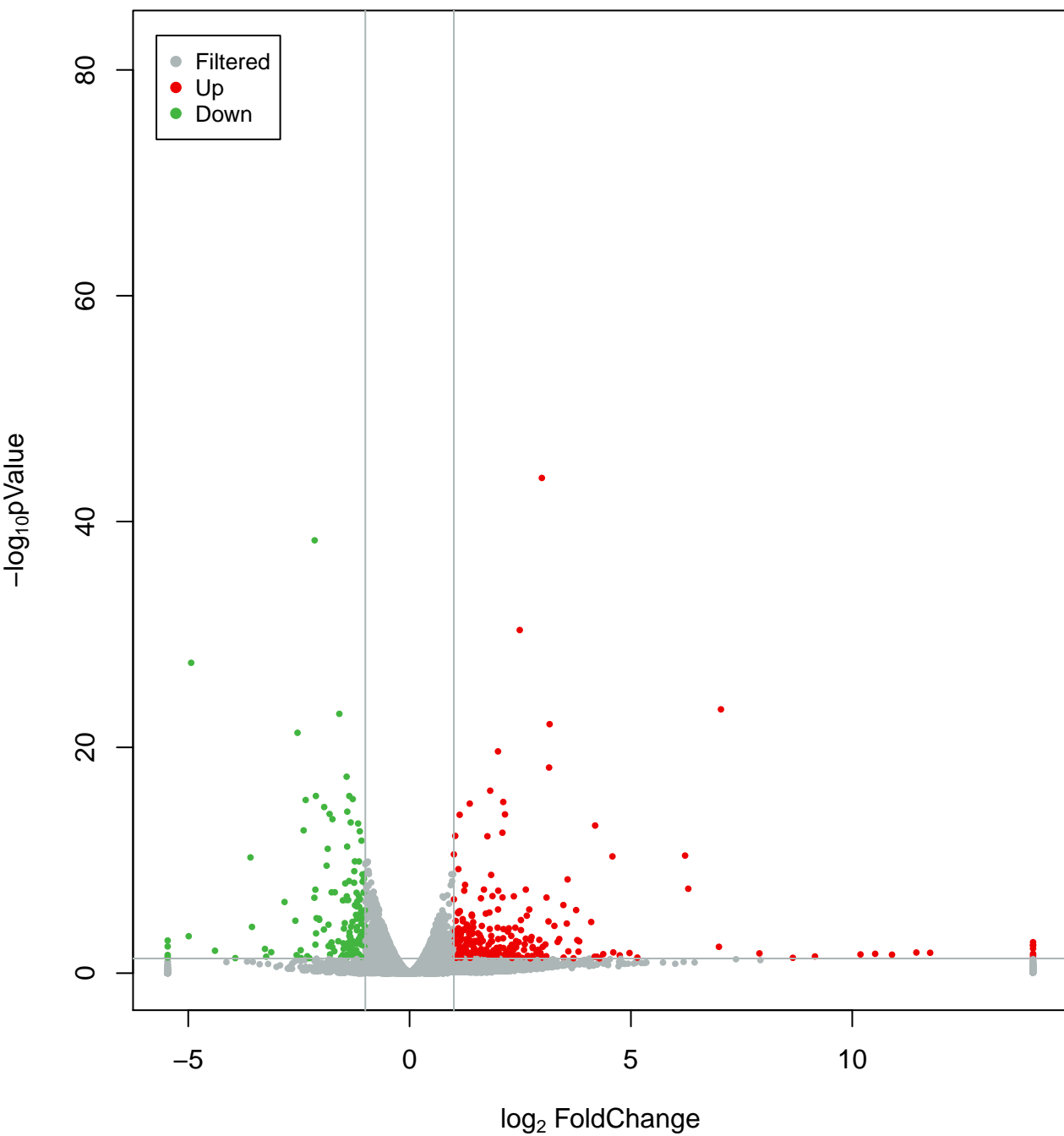

Supplement: Supplementary file 2 [file SupplementaryFile1.zip › Supplementary file 1/original RNAseq data/1.1.different_expressed_gene/MHC_1D-vs-WT_1D-volcano-pval-0.05-FC-2.gene.pdf]

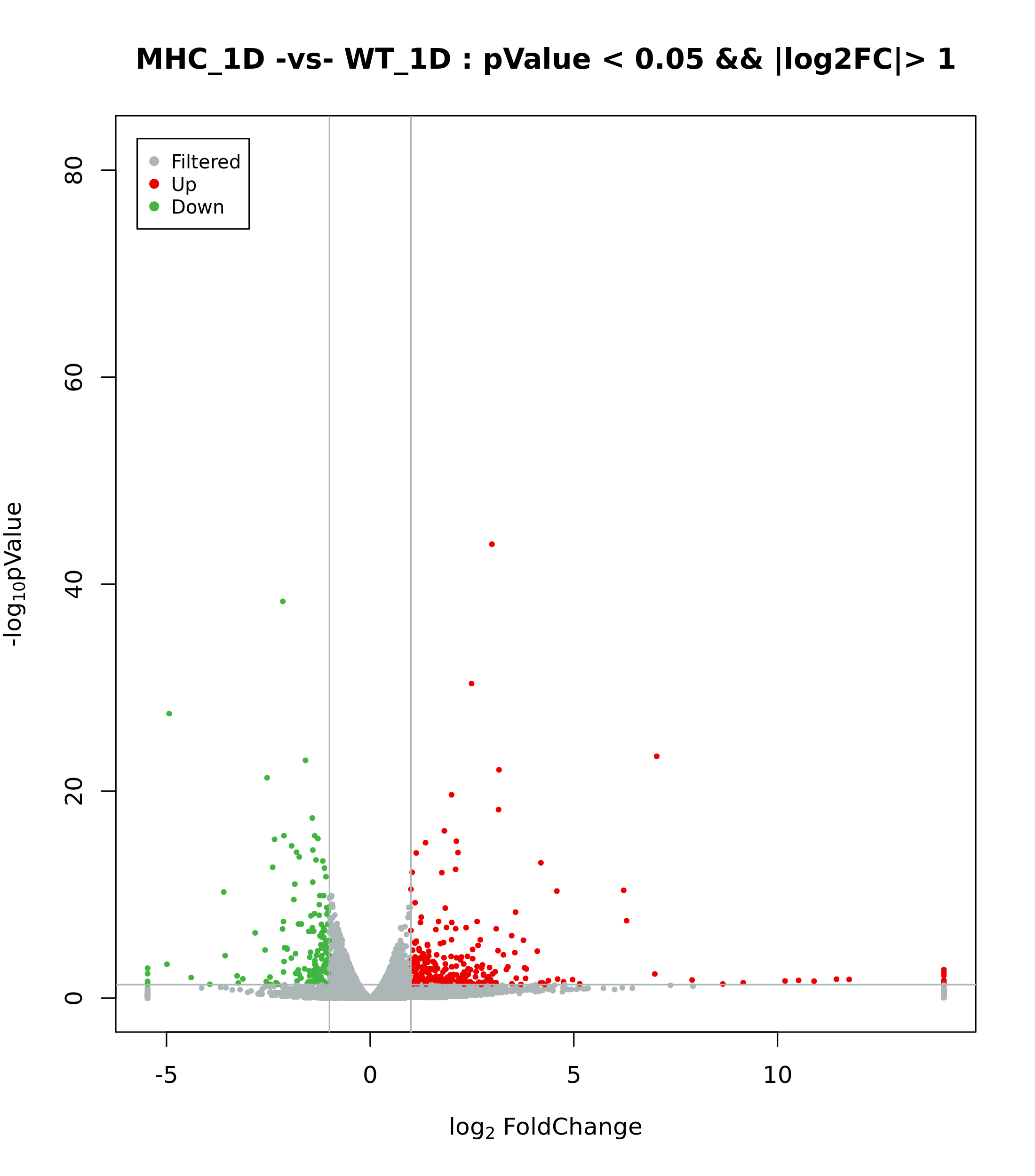

Supplement: Supplementary file 2 [file SupplementaryFile1.zip › Supplementary file 1/original RNAseq data/1.1.different_expressed_gene/MHC_1D-vs-WT_1D-volcano-pval-0.05-FC-2.gene.png]

# MHC\_3D-vs-MHC\_0D:pValue<0.05&& |log2FC|>1

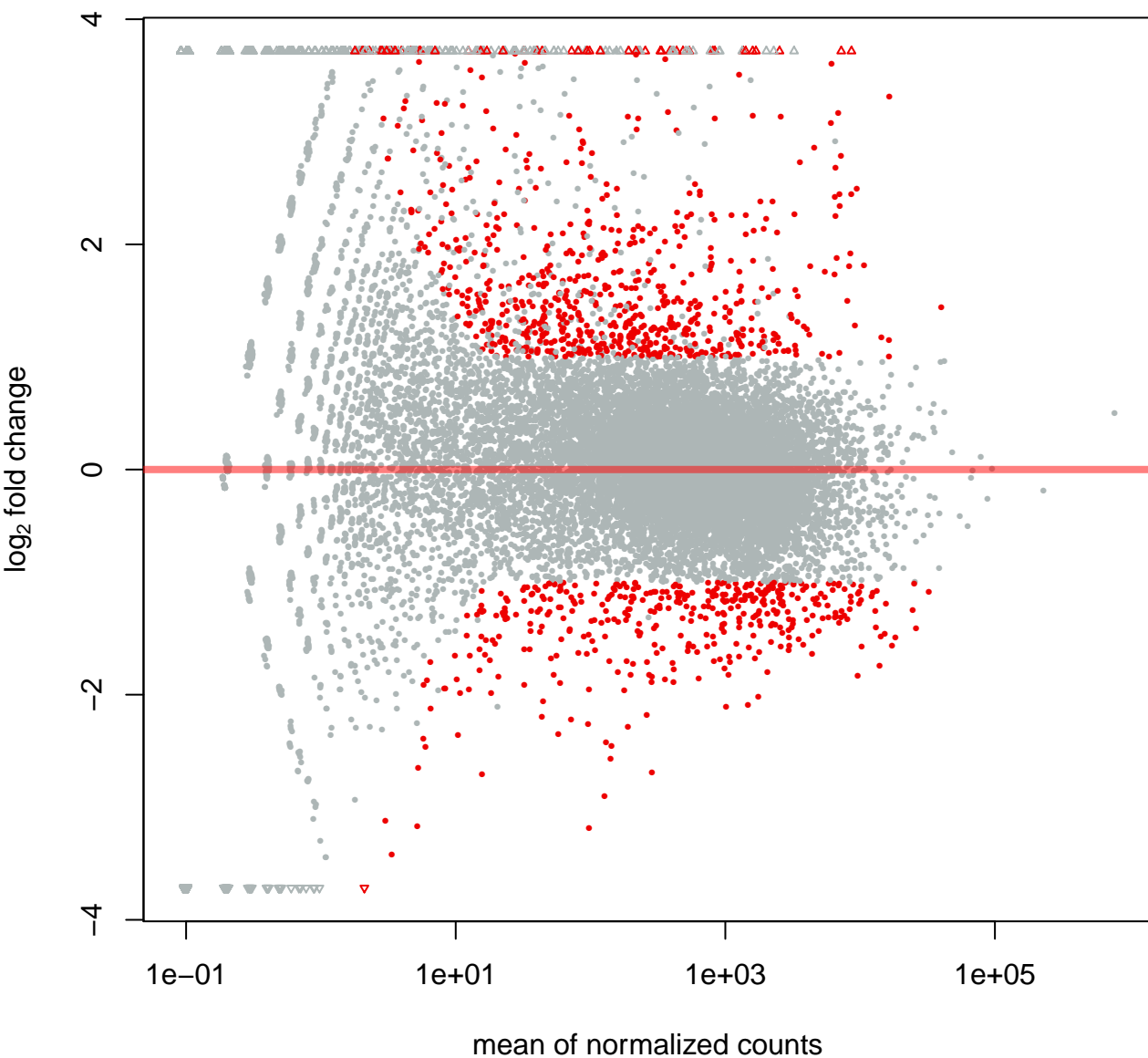

Supplement: Supplementary file 2 [file SupplementaryFile1.zip › Supplementary file 1/original RNAseq data/1.1.different_expressed_gene/MHC_3D-vs-MHC_0D-MA-pval-0.05-FC-2.gene.pdf]

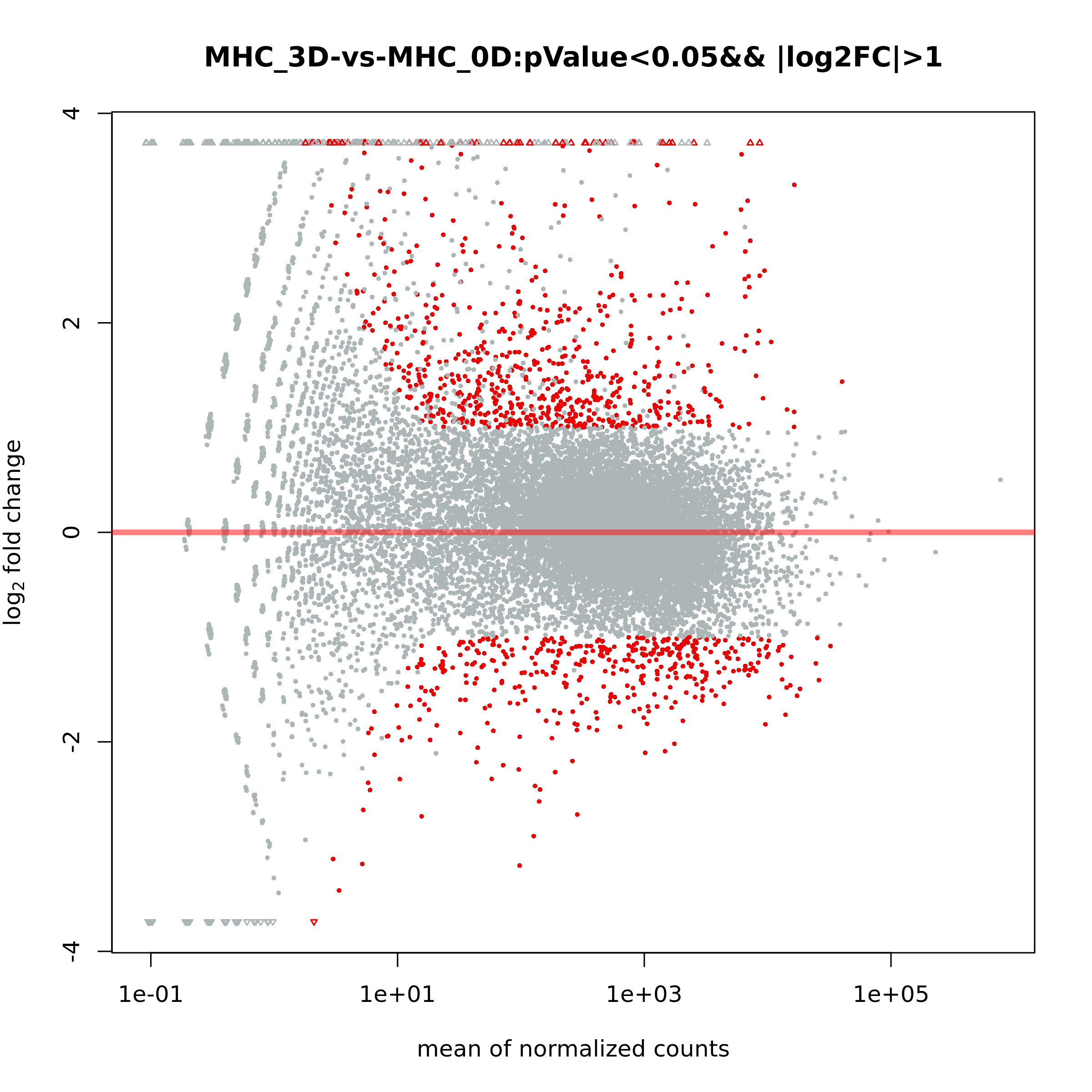

Supplement: Supplementary file 2 [file SupplementaryFile1.zip › Supplementary file 1/original RNAseq data/1.1.different_expressed_gene/MHC_3D-vs-MHC_0D-MA-pval-0.05-FC-2.gene.png]

MHC\_3D-vs-MHC\_0D:pValue<0.05&& |log2FC|>1

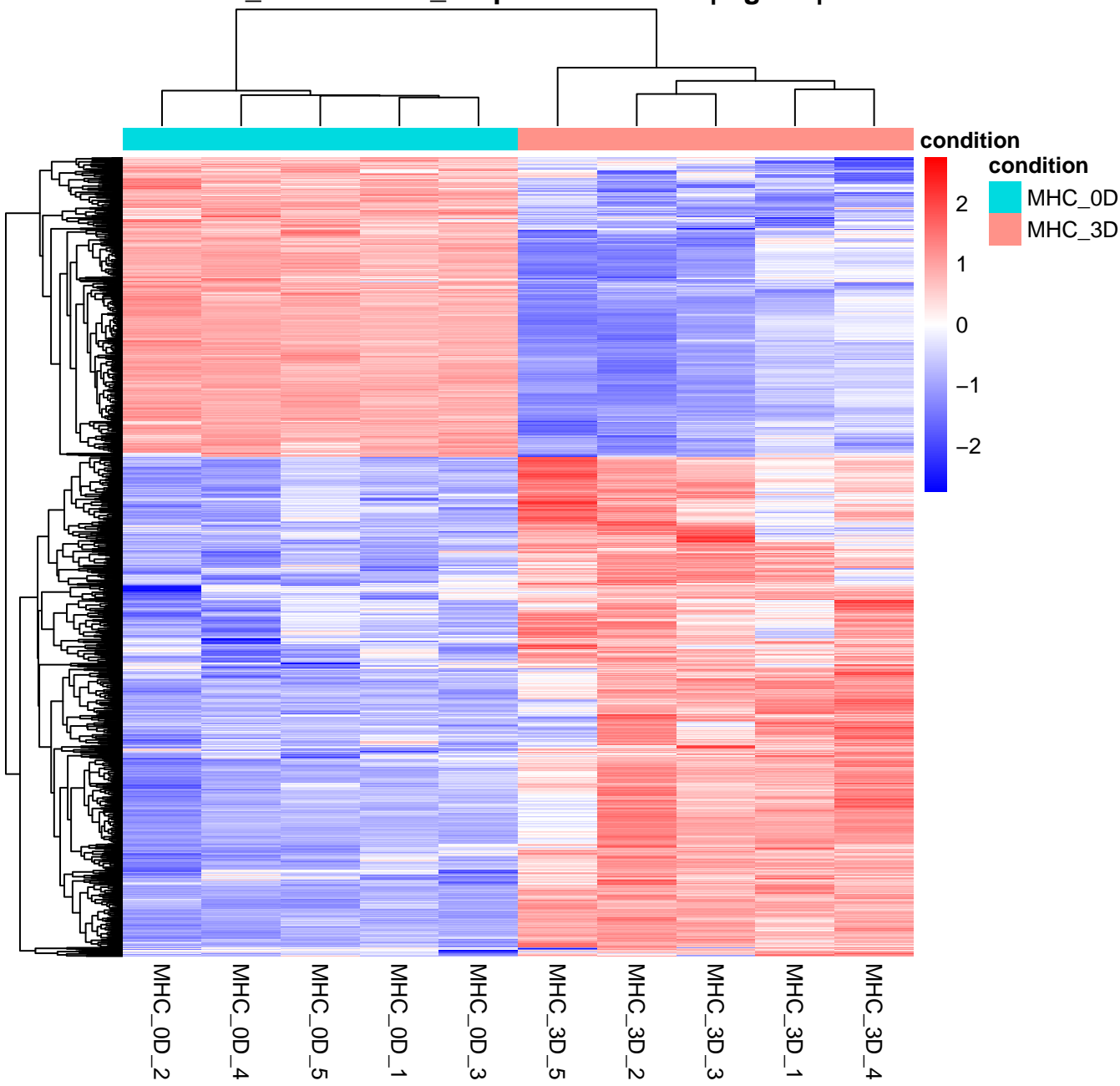

Supplement: Supplementary file 2 [file SupplementaryFile1.zip › Supplementary file 1/original RNAseq data/1.1.different_expressed_gene/MHC_3D-vs-MHC_0D-heatmap-pval-0.05-FC-2.gene.pdf]

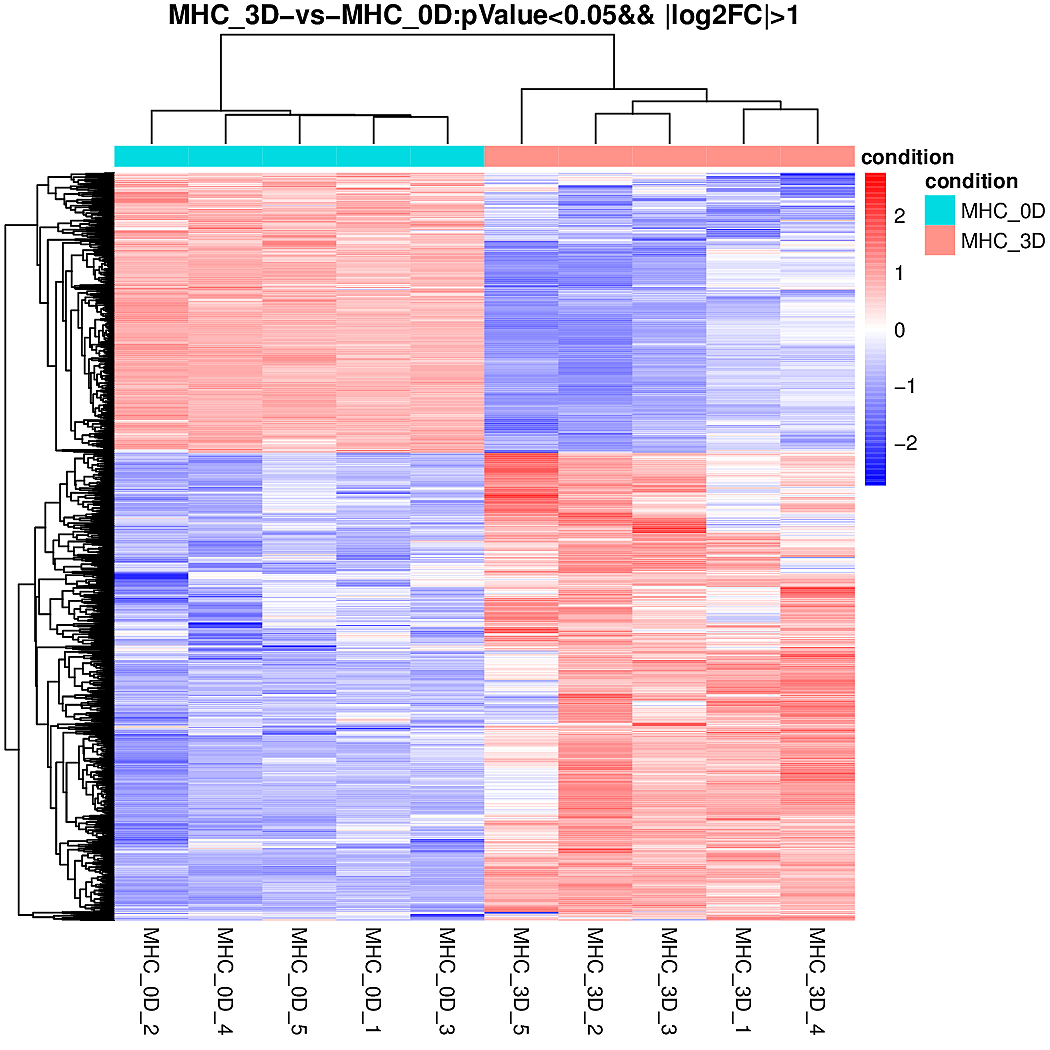

Supplement: Supplementary file 2 [file SupplementaryFile1.zip › Supplementary file 1/original RNAseq data/1.1.different_expressed_gene/MHC_3D-vs-MHC_0D-heatmap-pval-0.05-FC-2.gene.png]

# MHC\_3D -vs- MHC\_0D : pValue < 0.05 && |log2FC|> 1

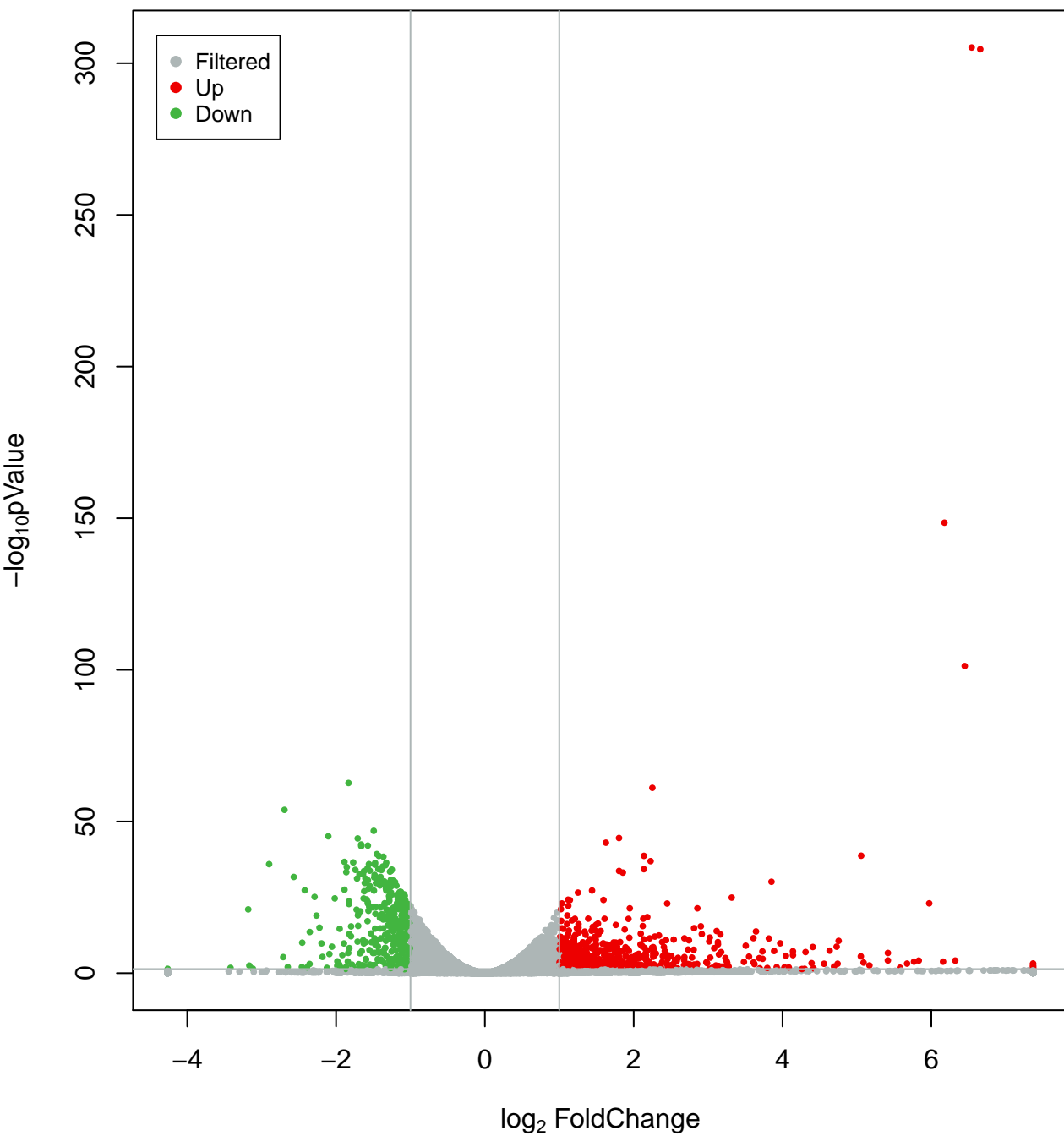

Supplement: Supplementary file 2 [file SupplementaryFile1.zip › Supplementary file 1/original RNAseq data/1.1.different_expressed_gene/MHC_3D-vs-MHC_0D-volcano-pval-0.05-FC-2.gene.pdf]

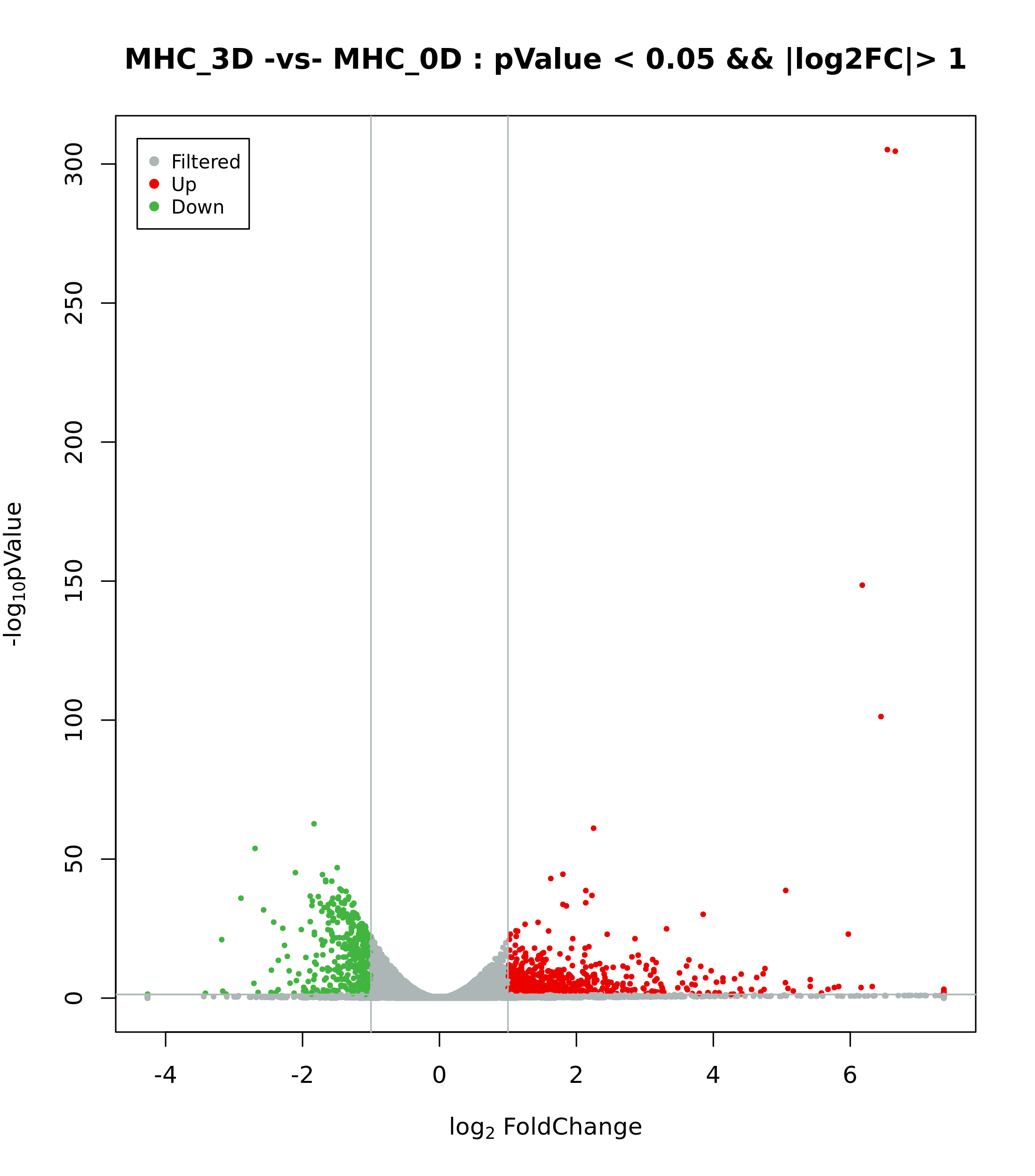

Supplement: Supplementary file 2 [file SupplementaryFile1.zip › Supplementary file 1/original RNAseq data/1.1.different_expressed_gene/MHC_3D-vs-MHC_0D-volcano-pval-0.05-FC-2.gene.png]

# MHC\_3D-vs-WT\_3D:pValue<0.05&& |log2FC|>1

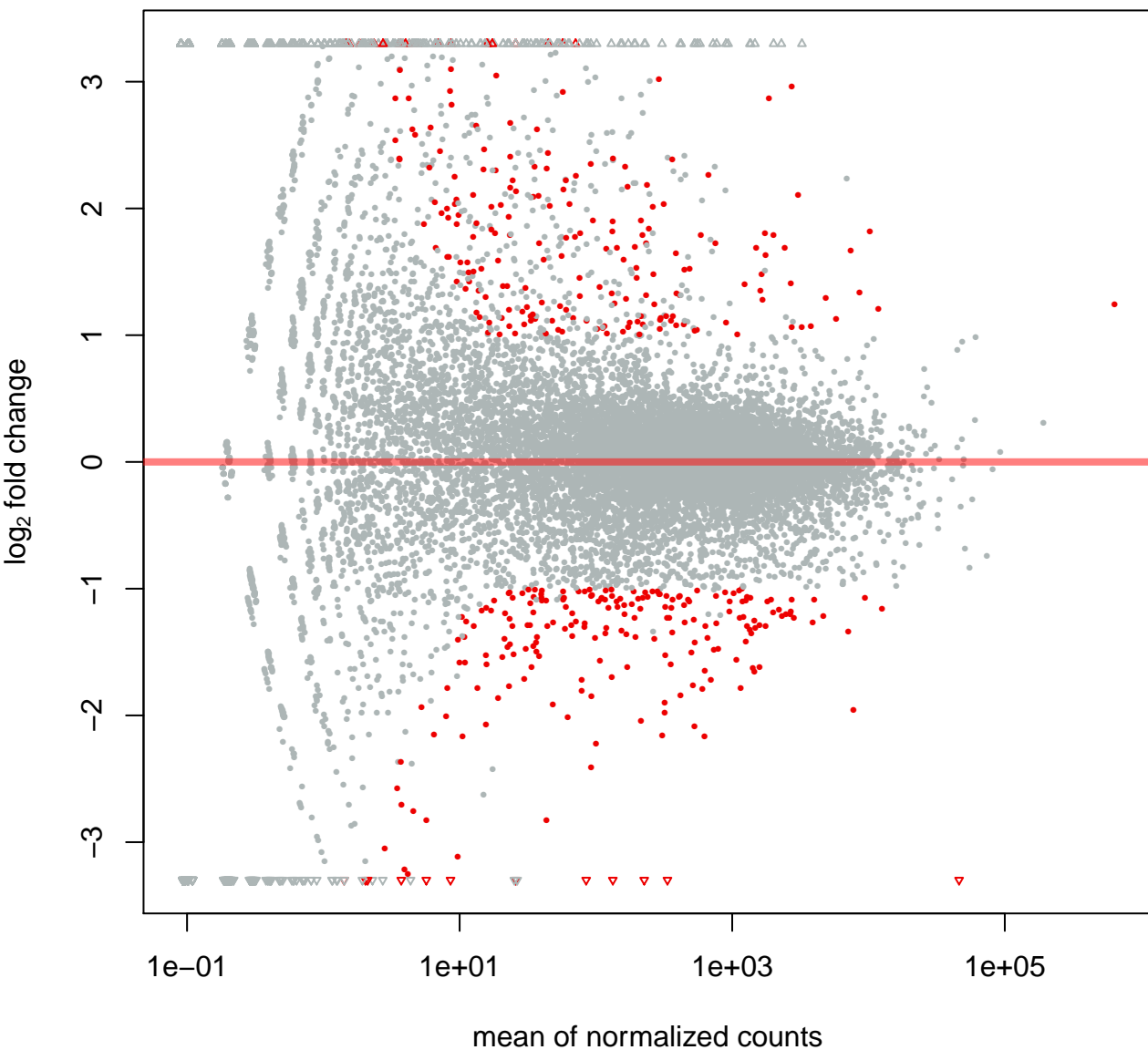

Supplement: Supplementary file 2 [file SupplementaryFile1.zip › Supplementary file 1/original RNAseq data/1.1.different_expressed_gene/MHC_3D-vs-WT_3D-MA-pval-0.05-FC-2.gene.pdf]

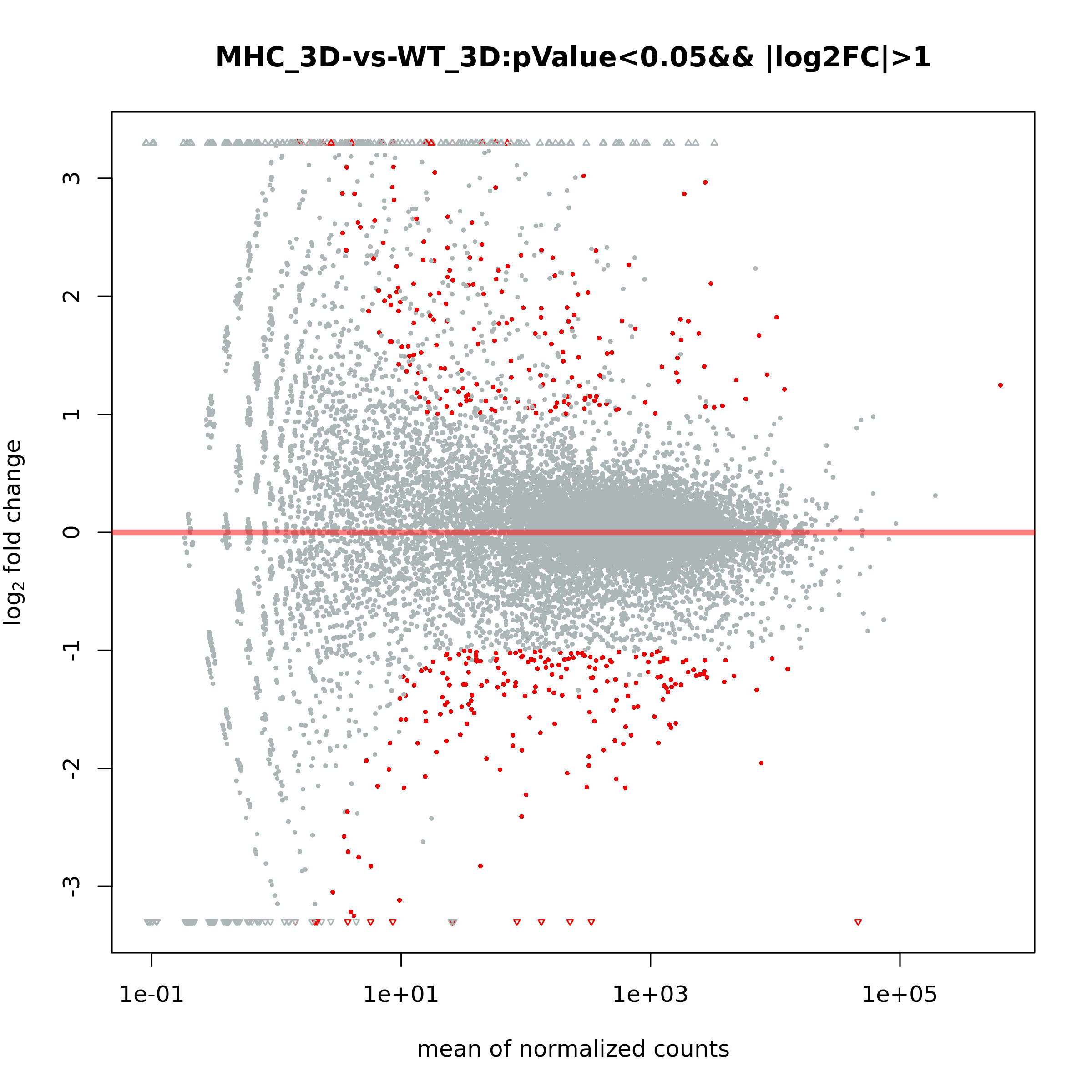

Supplement: Supplementary file 2 [file SupplementaryFile1.zip › Supplementary file 1/original RNAseq data/1.1.different_expressed_gene/MHC_3D-vs-WT_3D-MA-pval-0.05-FC-2.gene.png]

MHC\_3D-vs-WT\_3D:pValue<0.05&& |log2FC|>1

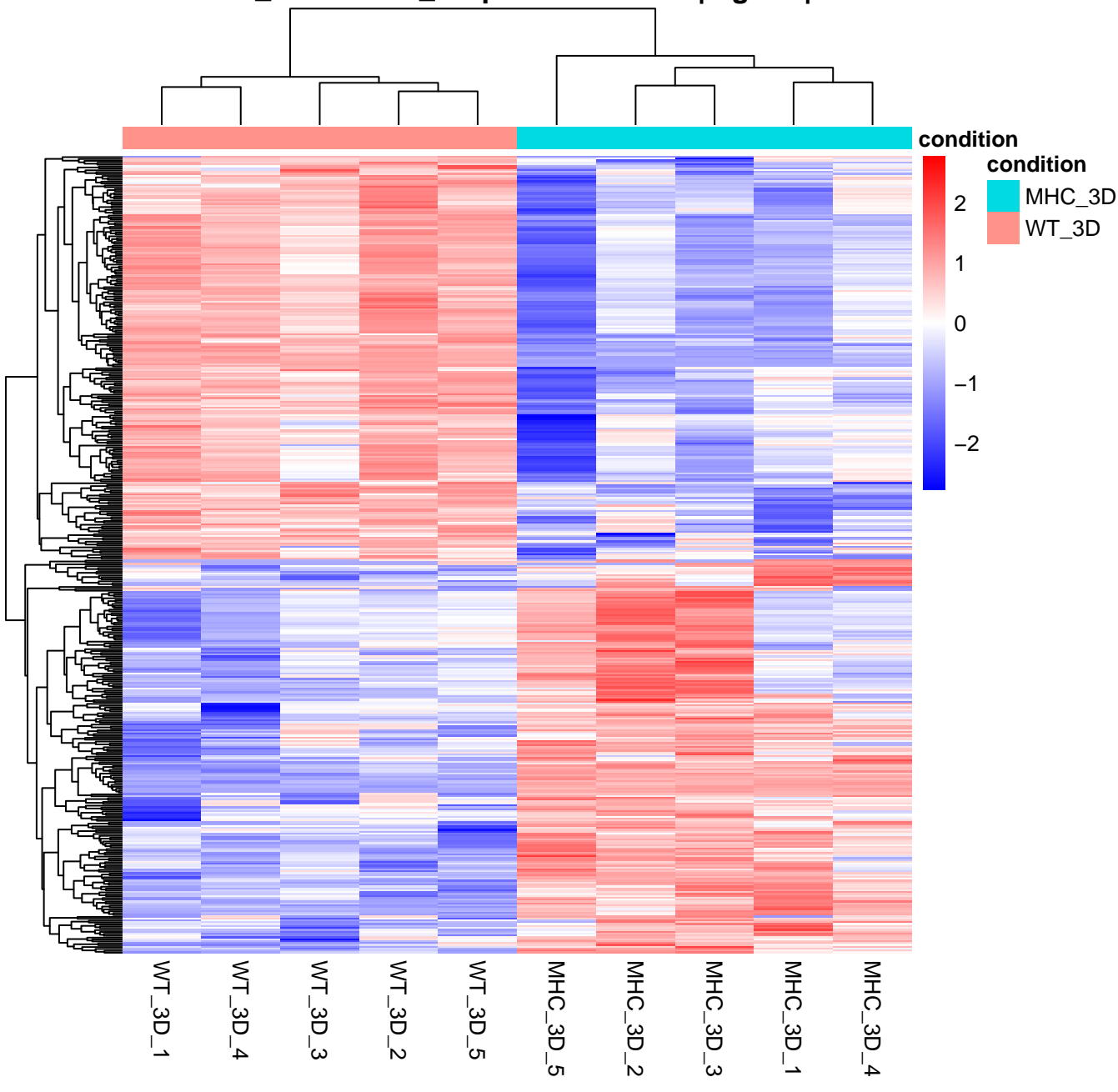

Supplement: Supplementary file 2 [file SupplementaryFile1.zip › Supplementary file 1/original RNAseq data/1.1.different_expressed_gene/MHC_3D-vs-WT_3D-heatmap-pval-0.05-FC-2.gene.pdf]

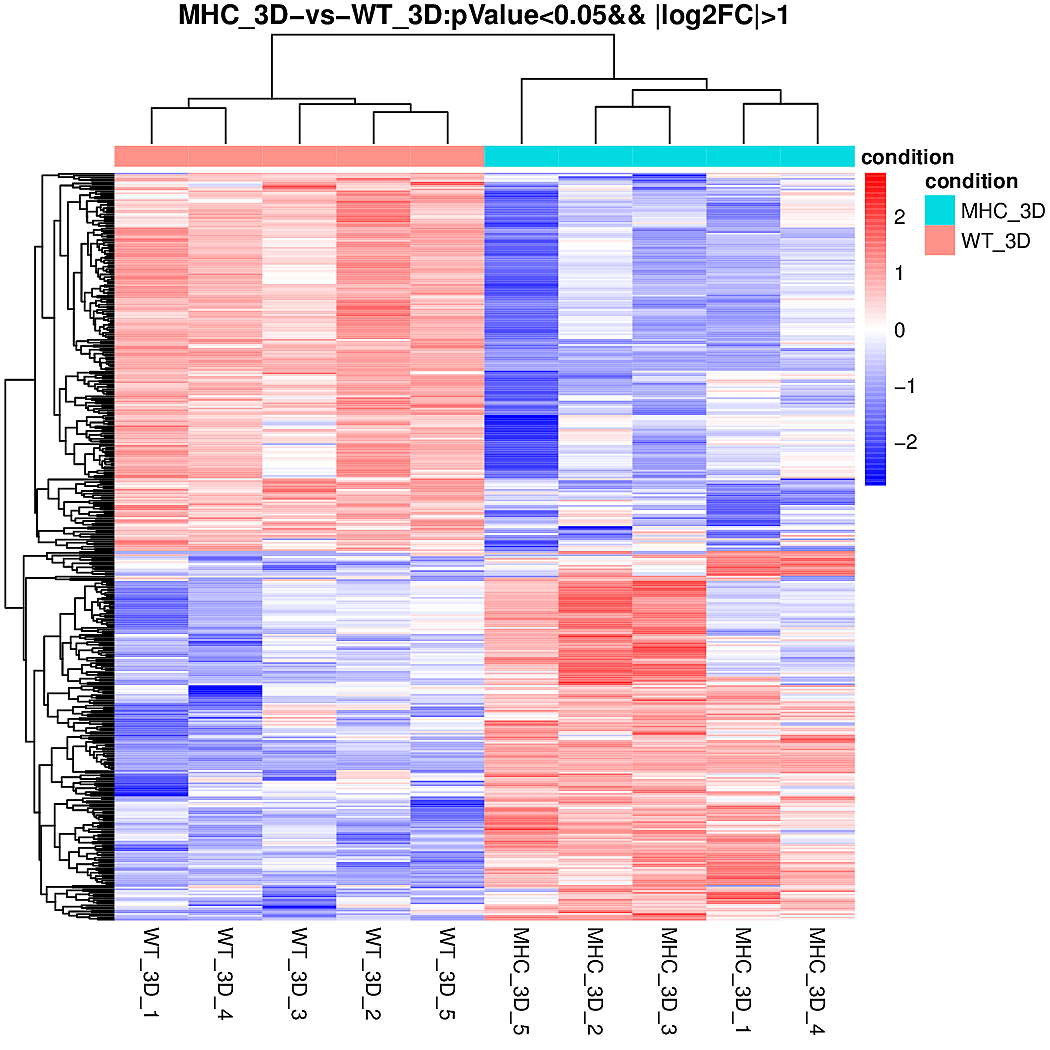

Supplement: Supplementary file 2 [file SupplementaryFile1.zip › Supplementary file 1/original RNAseq data/1.1.different_expressed_gene/MHC_3D-vs-WT_3D-heatmap-pval-0.05-FC-2.gene.png]

# MHC\_3D -vs- WT\_3D : pValue < 0.05 && |log2FC|> 1

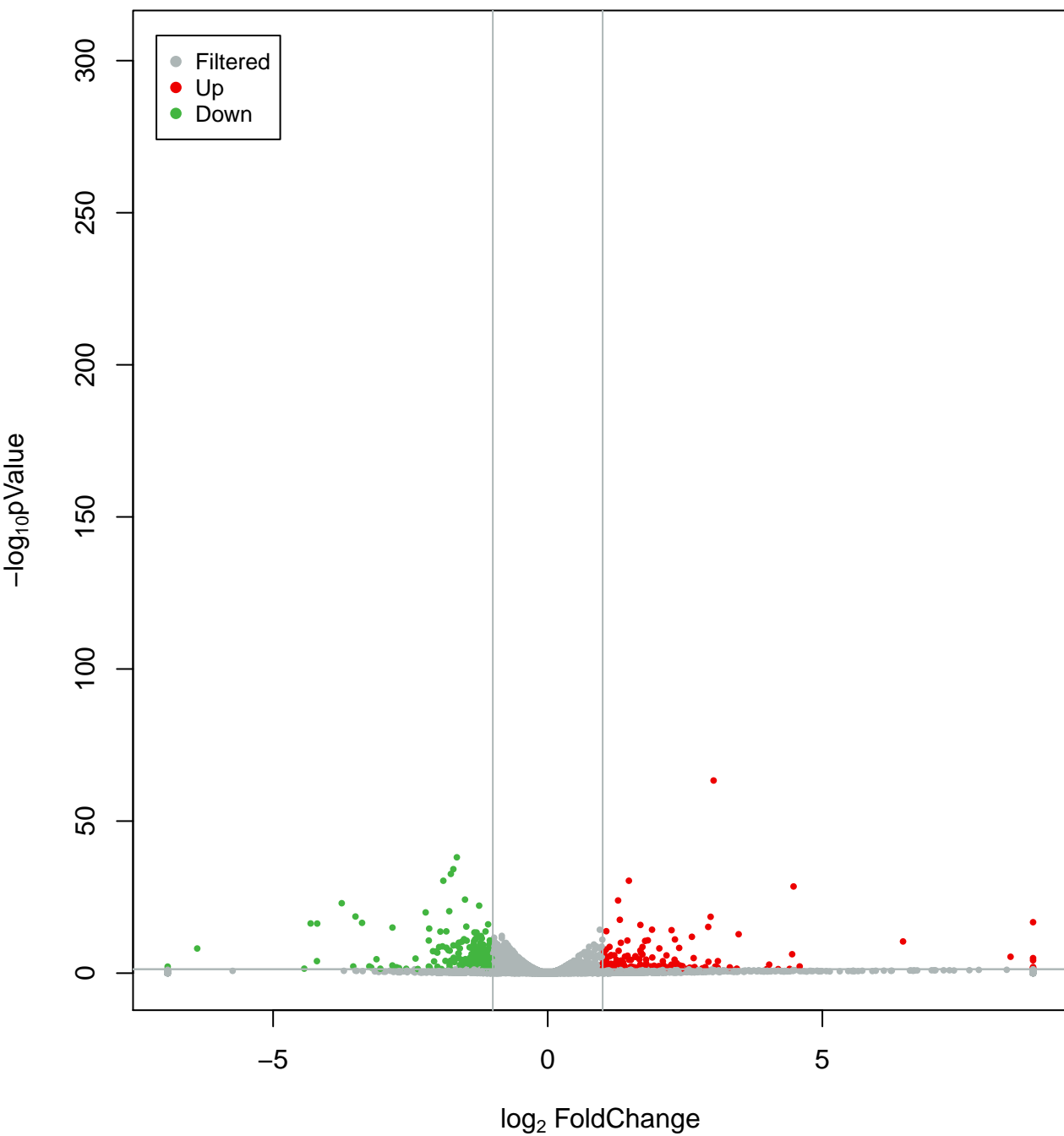

Supplement: Supplementary file 2 [file SupplementaryFile1.zip › Supplementary file 1/original RNAseq data/1.1.different_expressed_gene/MHC_3D-vs-WT_3D-volcano-pval-0.05-FC-2.gene.pdf]

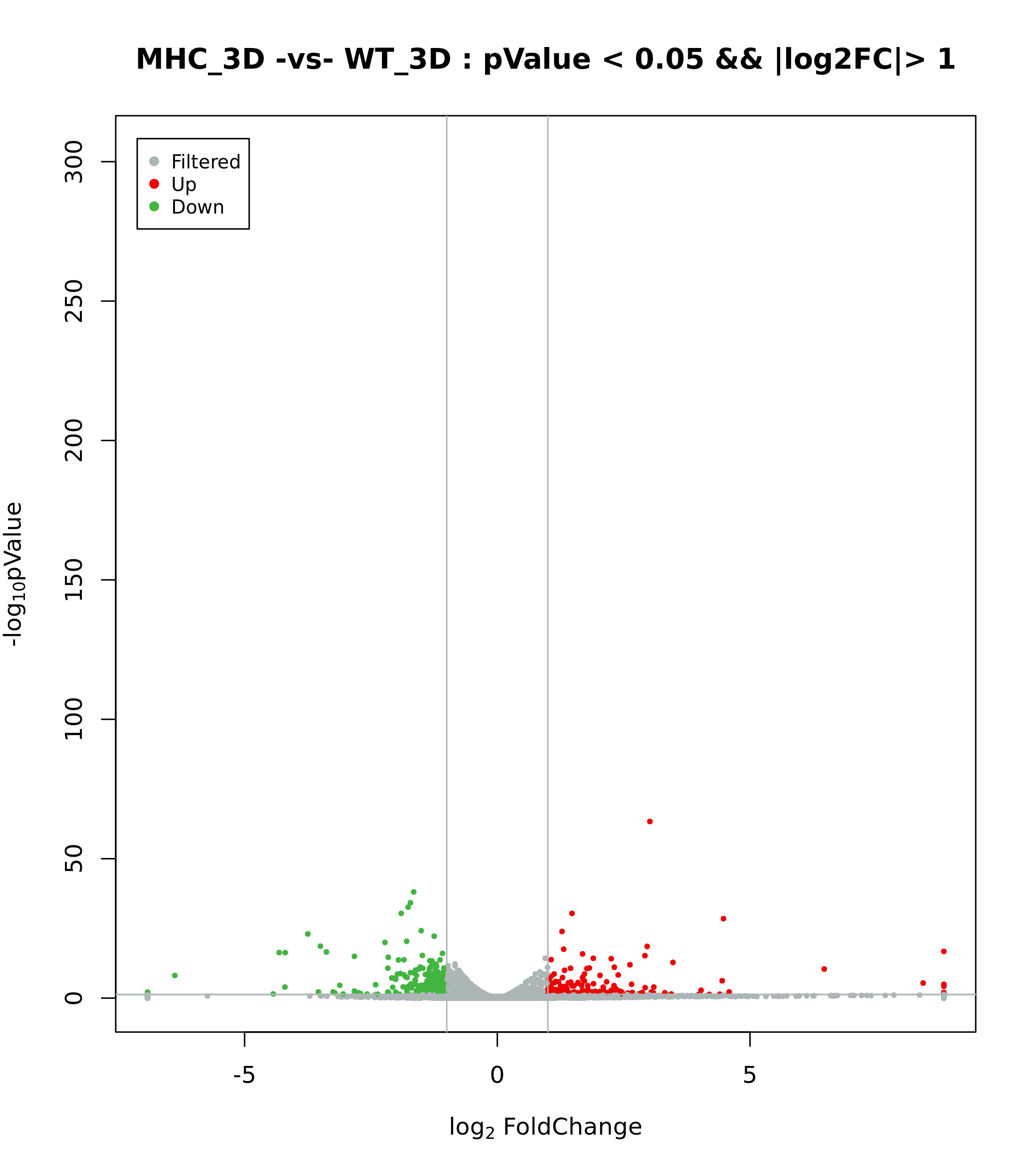

Supplement: Supplementary file 2 [file SupplementaryFile1.zip › Supplementary file 1/original RNAseq data/1.1.different_expressed_gene/MHC_3D-vs-WT_3D-volcano-pval-0.05-FC-2.gene.png]

# MHC\_6D-vs-MHC\_0D:pValue<0.05&& |log2FC|>1

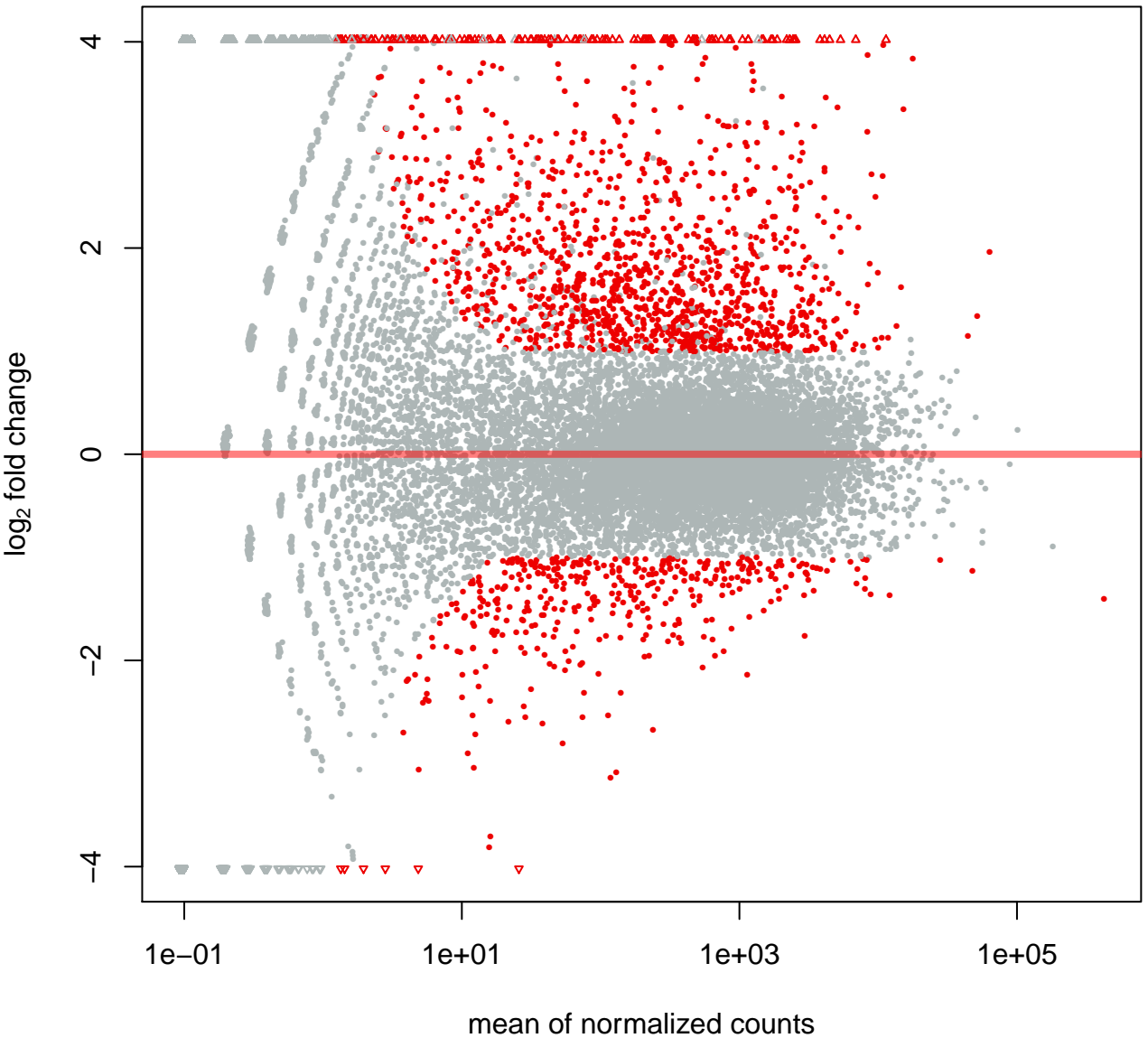

Supplement: Supplementary file 2 [file SupplementaryFile1.zip › Supplementary file 1/original RNAseq data/1.1.different_expressed_gene/MHC_6D-vs-MHC_0D-MA-pval-0.05-FC-2.gene.pdf]

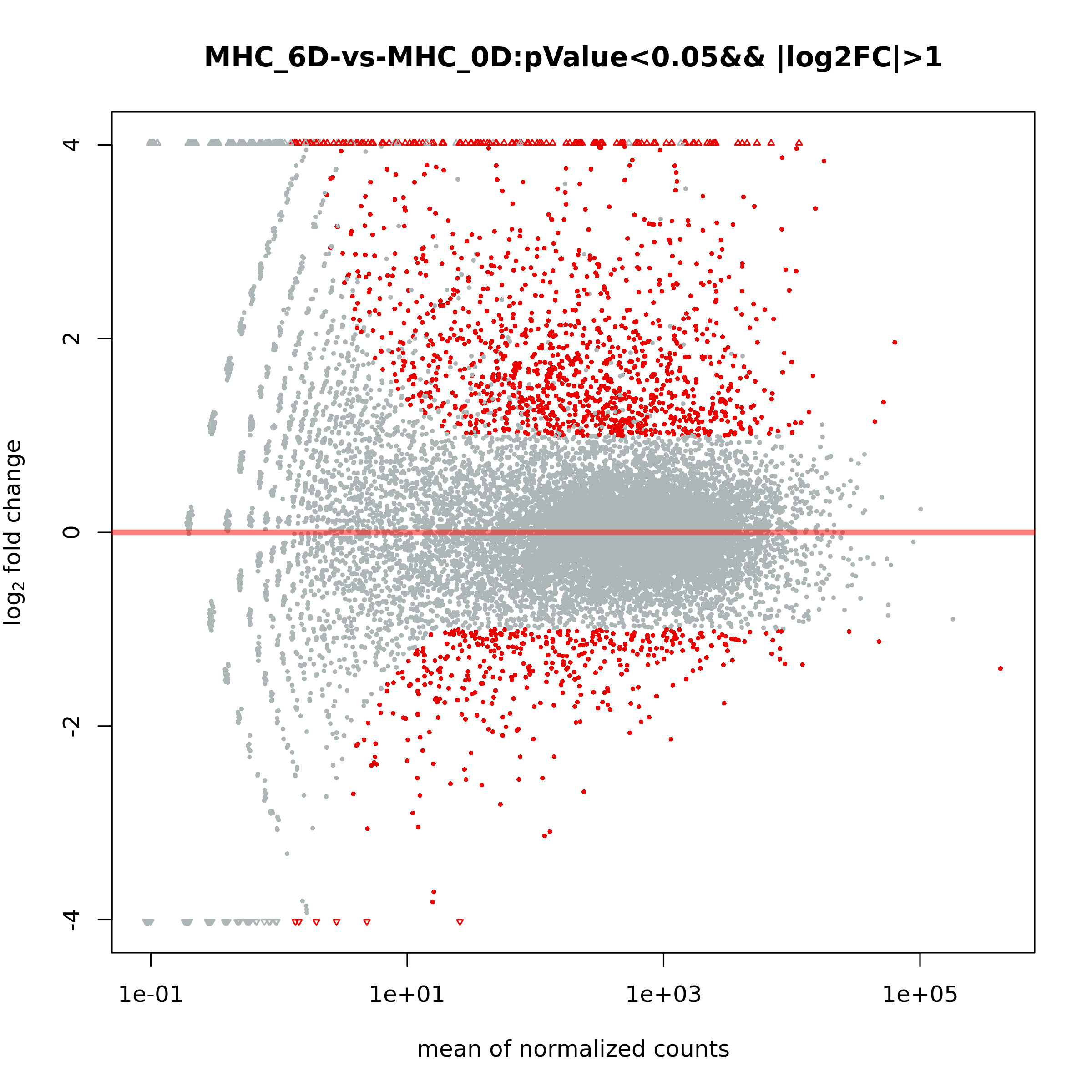

Supplement: Supplementary file 2 [file SupplementaryFile1.zip › Supplementary file 1/original RNAseq data/1.1.different_expressed_gene/MHC_6D-vs-MHC_0D-MA-pval-0.05-FC-2.gene.png]

MHC\_6D-vs-MHC\_0D:pValue<0.05&& |log2FC|>1

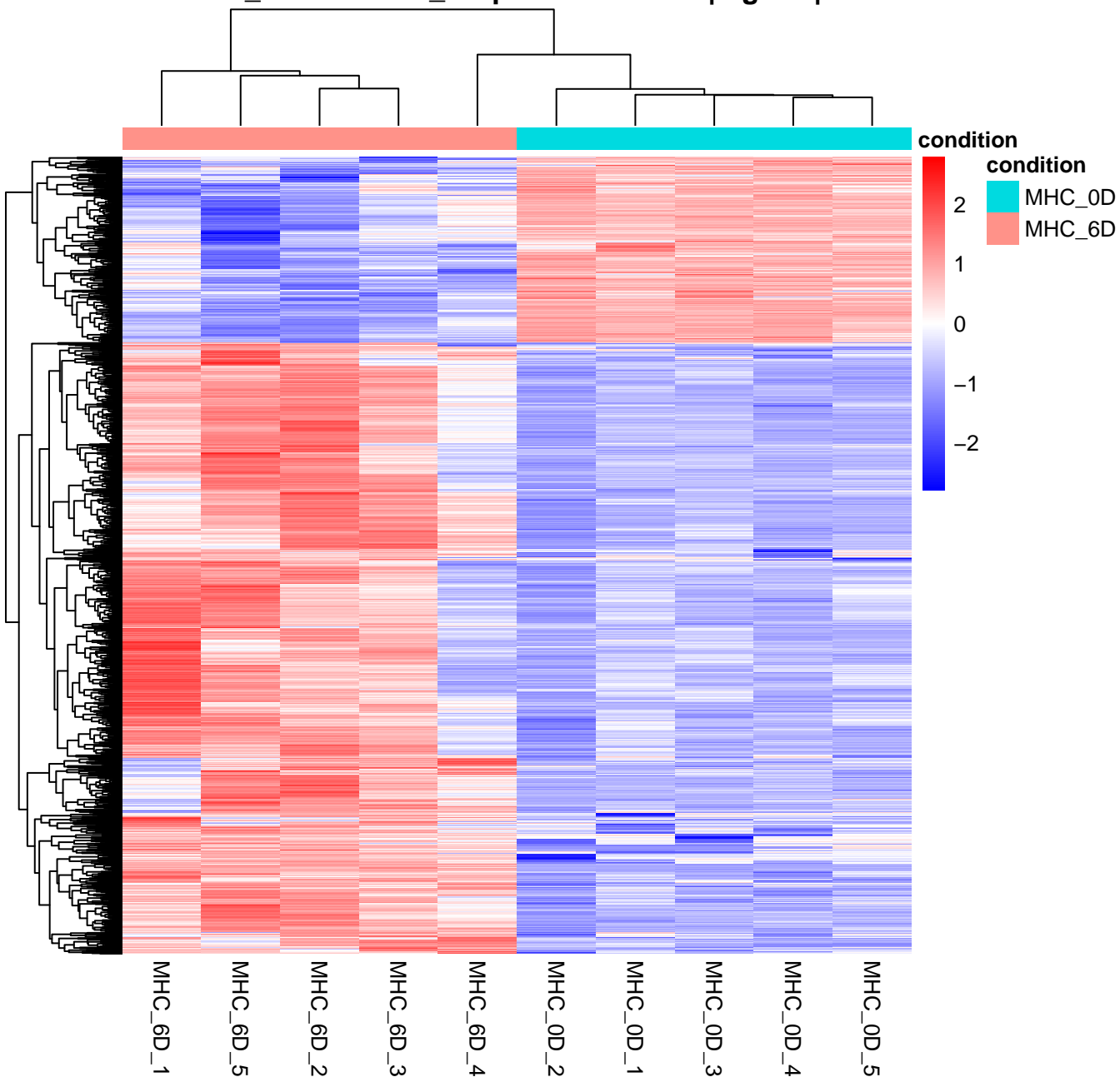

Supplement: Supplementary file 2 [file SupplementaryFile1.zip › Supplementary file 1/original RNAseq data/1.1.different_expressed_gene/MHC_6D-vs-MHC_0D-heatmap-pval-0.05-FC-2.gene.pdf]

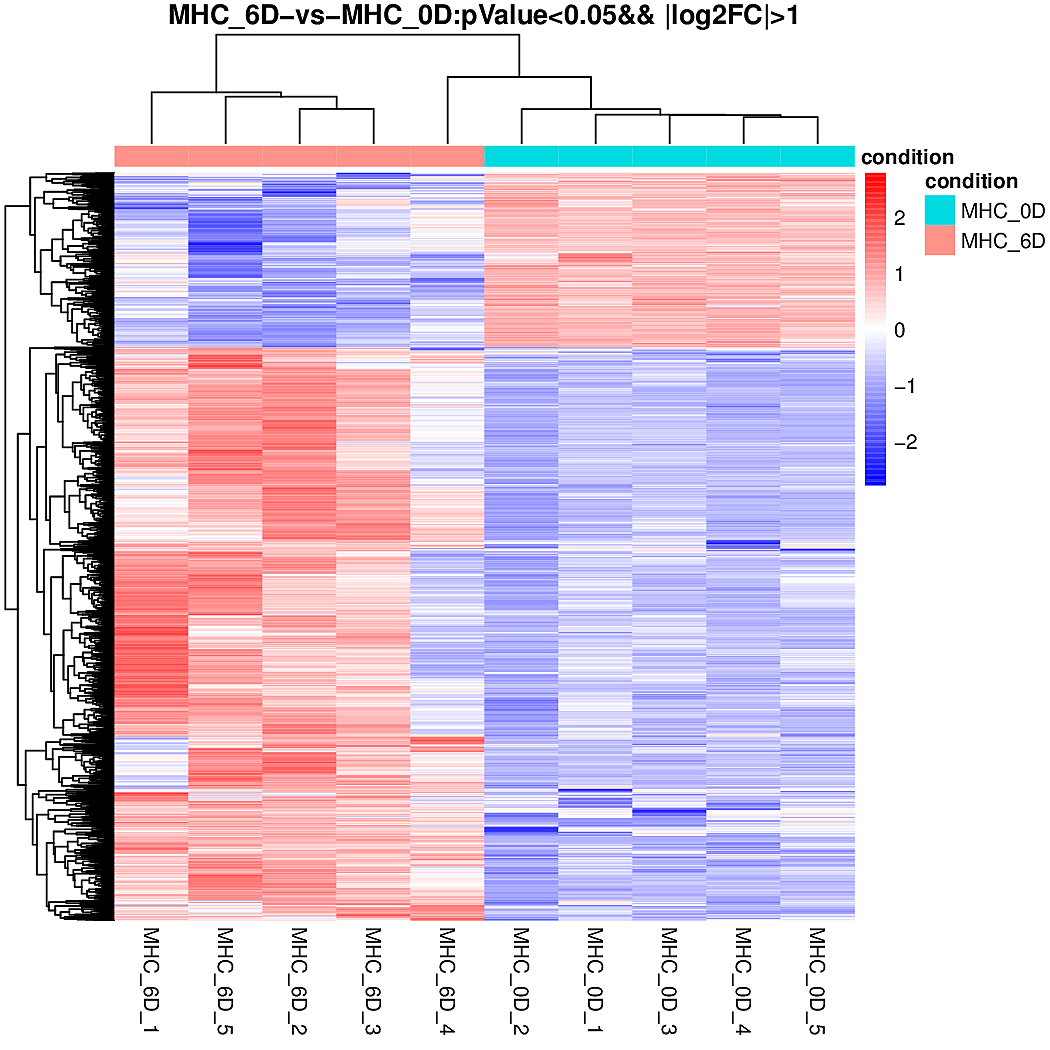

Supplement: Supplementary file 2 [file SupplementaryFile1.zip › Supplementary file 1/original RNAseq data/1.1.different_expressed_gene/MHC_6D-vs-MHC_0D-heatmap-pval-0.05-FC-2.gene.png]

# MHC\_6D -vs- MHC\_0D : pValue < 0.05 && |log2FC|> 1

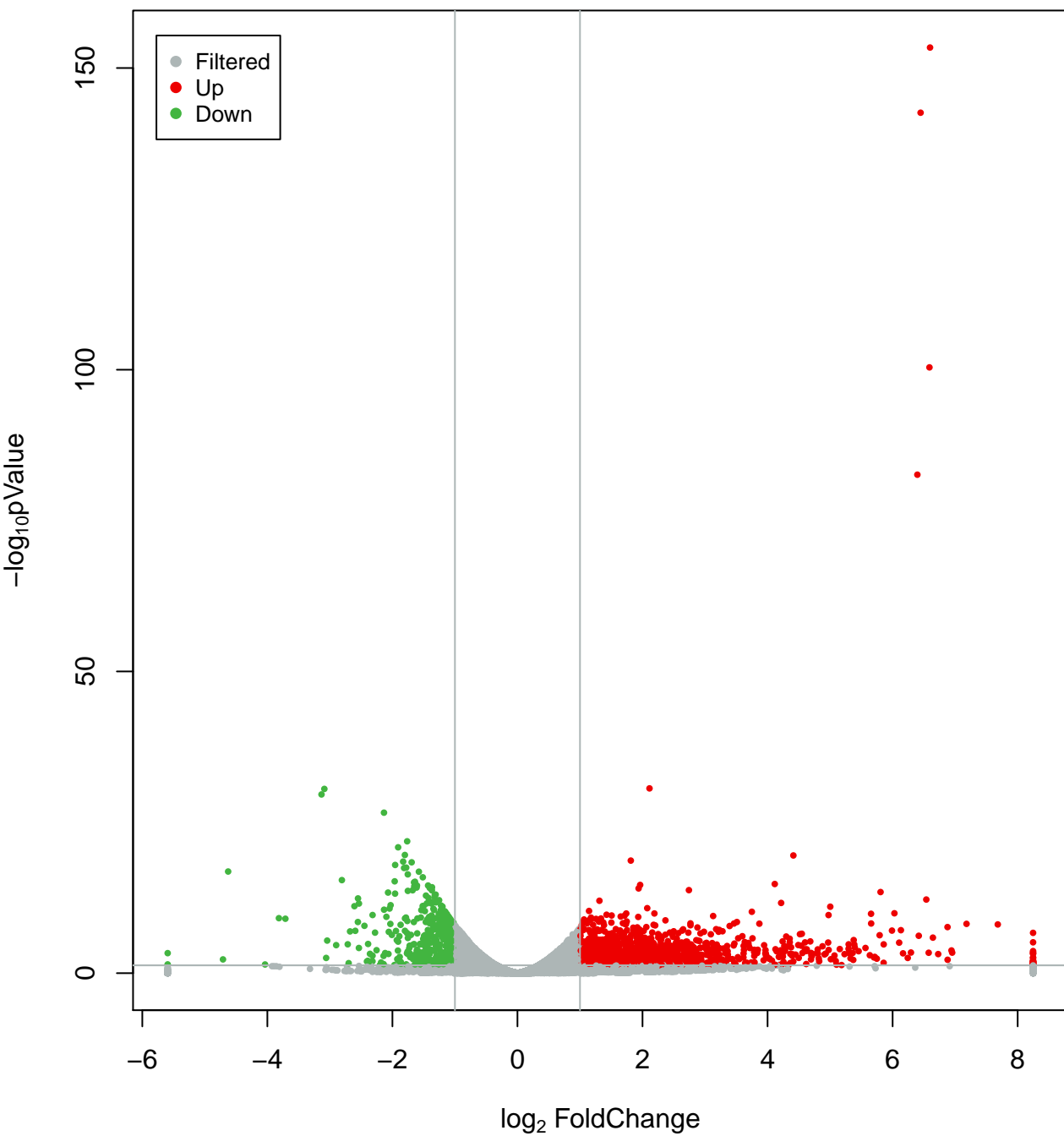

Supplement: Supplementary file 2 [file SupplementaryFile1.zip › Supplementary file 1/original RNAseq data/1.1.different_expressed_gene/MHC_6D-vs-MHC_0D-volcano-pval-0.05-FC-2.gene.pdf]

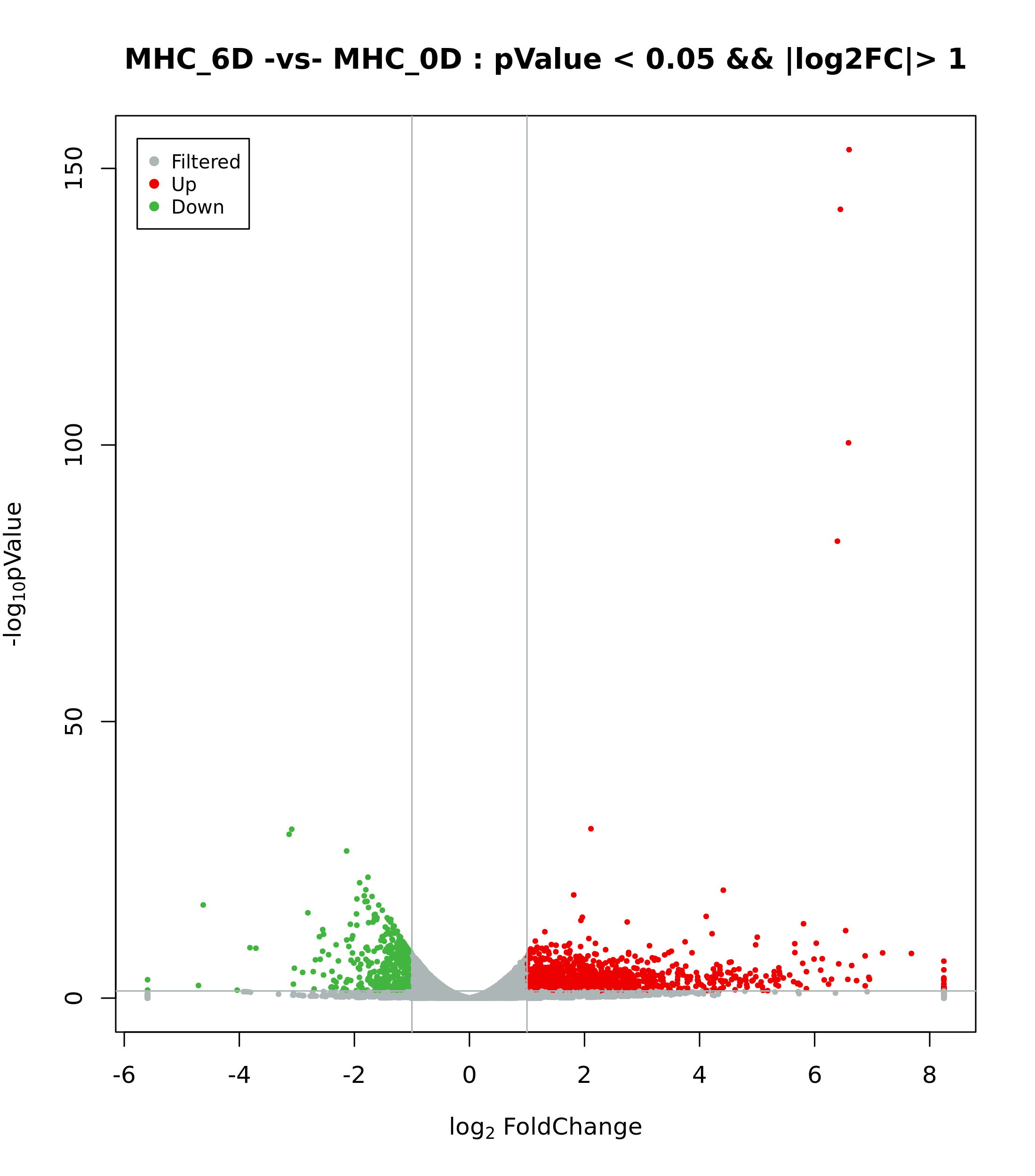

Supplement: Supplementary file 2 [file SupplementaryFile1.zip › Supplementary file 1/original RNAseq data/1.1.different_expressed_gene/MHC_6D-vs-MHC_0D-volcano-pval-0.05-FC-2.gene.png]

# MHC\_6D-vs-WT\_6D:pValue<0.05&& |log2FC|>1

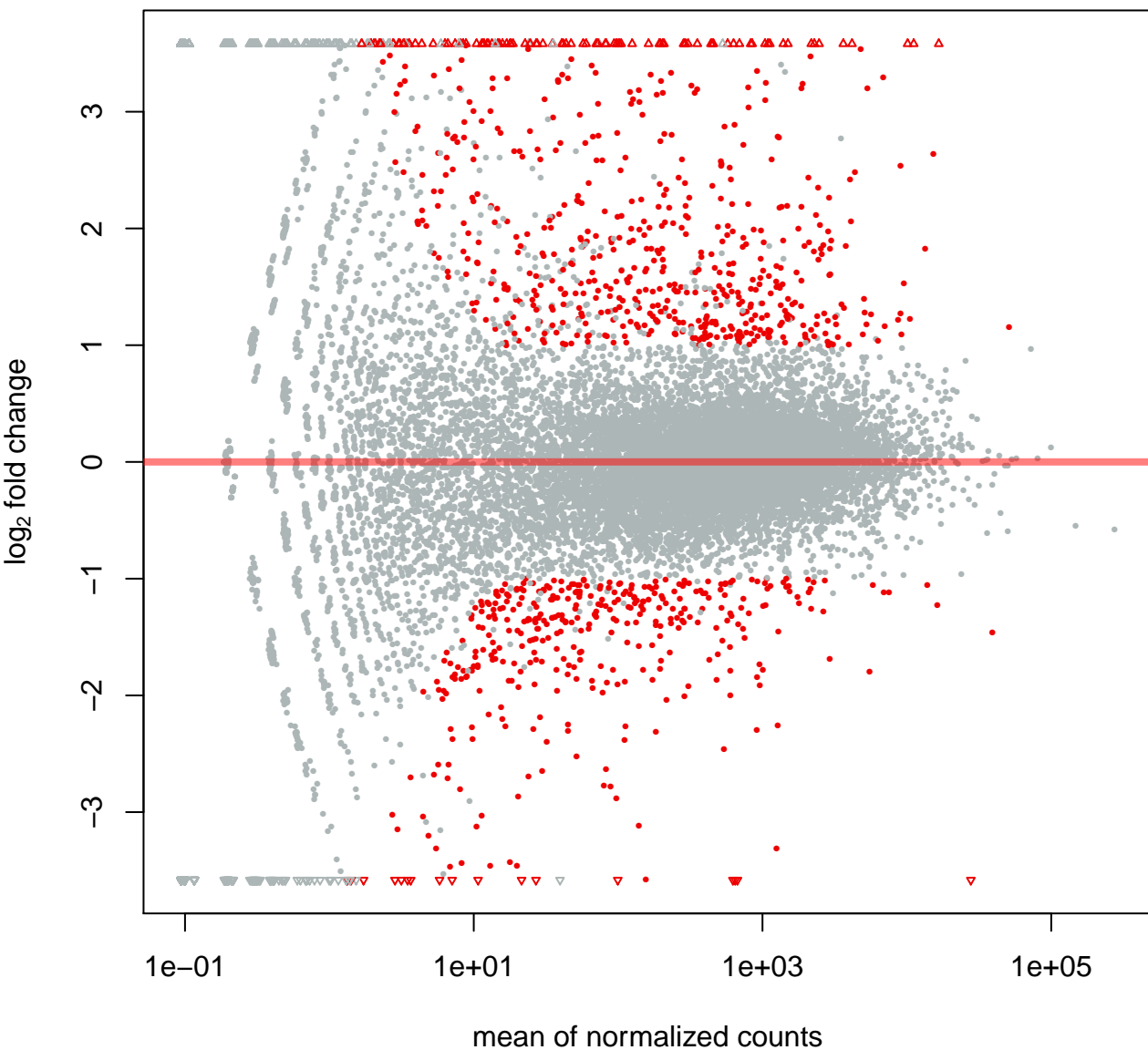

Supplement: Supplementary file 2 [file SupplementaryFile1.zip › Supplementary file 1/original RNAseq data/1.1.different_expressed_gene/MHC_6D-vs-WT_6D-MA-pval-0.05-FC-2.gene.pdf]

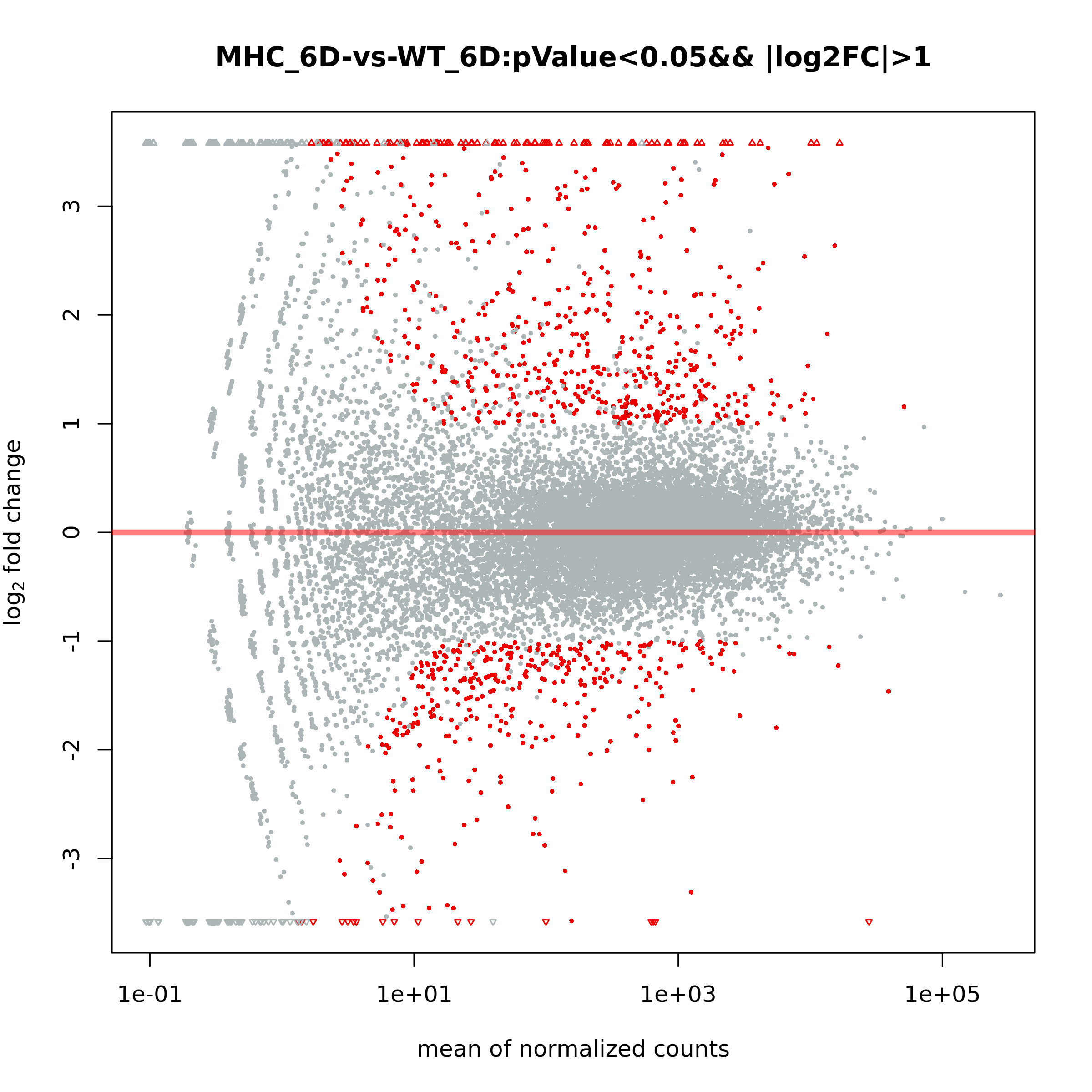

Supplement: Supplementary file 2 [file SupplementaryFile1.zip › Supplementary file 1/original RNAseq data/1.1.different_expressed_gene/MHC_6D-vs-WT_6D-MA-pval-0.05-FC-2.gene.png]

MHC\_6D-vs-WT\_6D:pValue<0.05&& |log2FC|>1

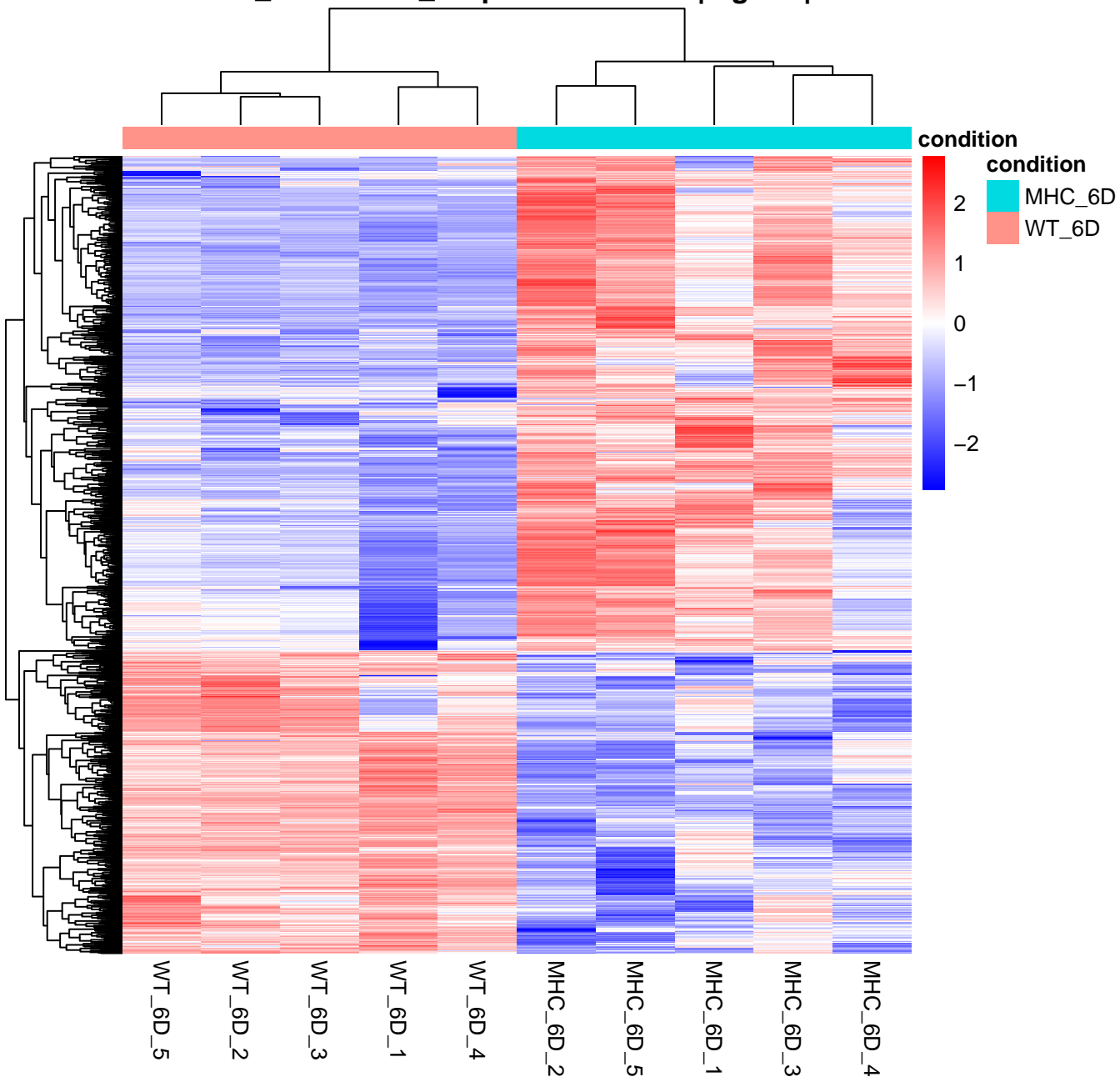

Supplement: Supplementary file 2 [file SupplementaryFile1.zip › Supplementary file 1/original RNAseq data/1.1.different_expressed_gene/MHC_6D-vs-WT_6D-heatmap-pval-0.05-FC-2.gene.pdf]

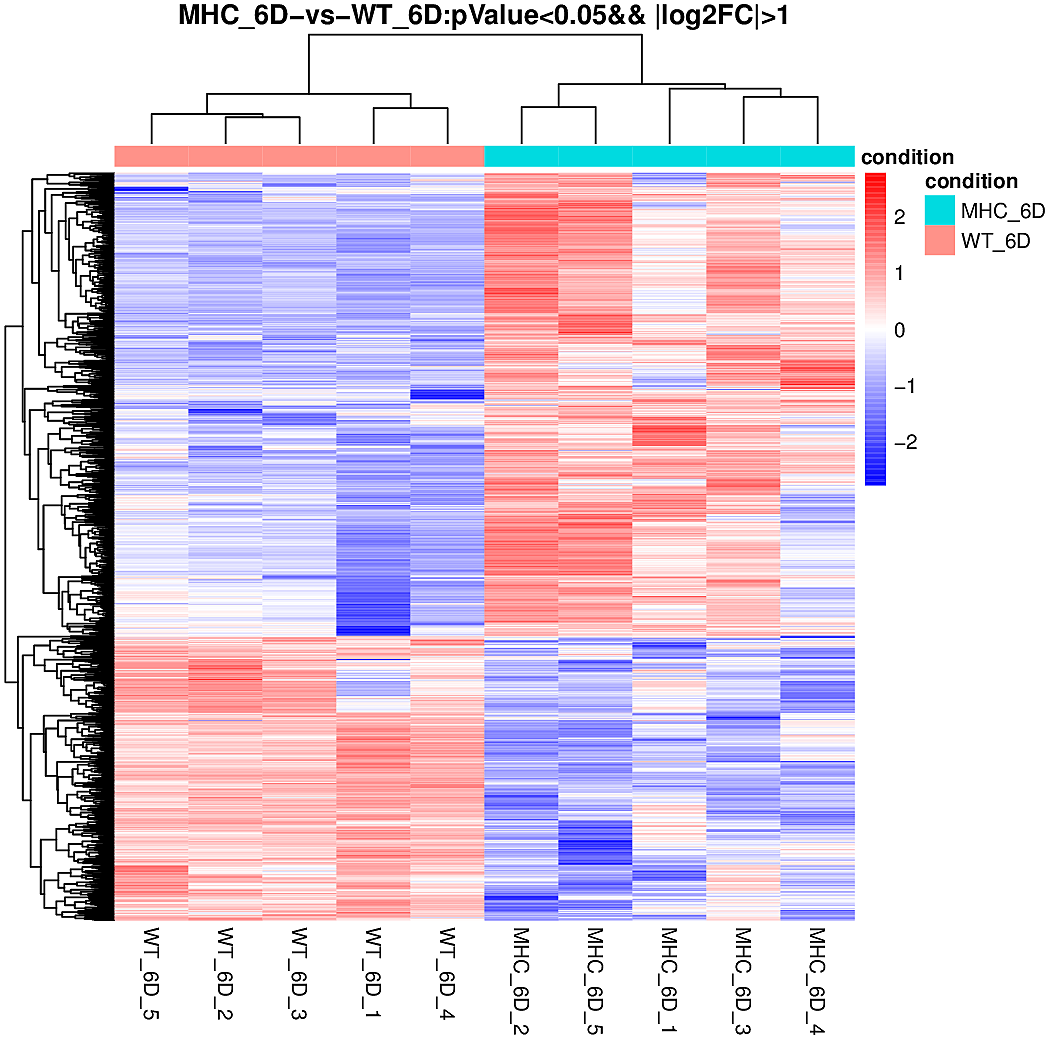

Supplement: Supplementary file 2 [file SupplementaryFile1.zip › Supplementary file 1/original RNAseq data/1.1.different_expressed_gene/MHC_6D-vs-WT_6D-heatmap-pval-0.05-FC-2.gene.png]

# MHC\_6D -vs- WT\_6D : pValue < 0.05 && |log2FC|> 1

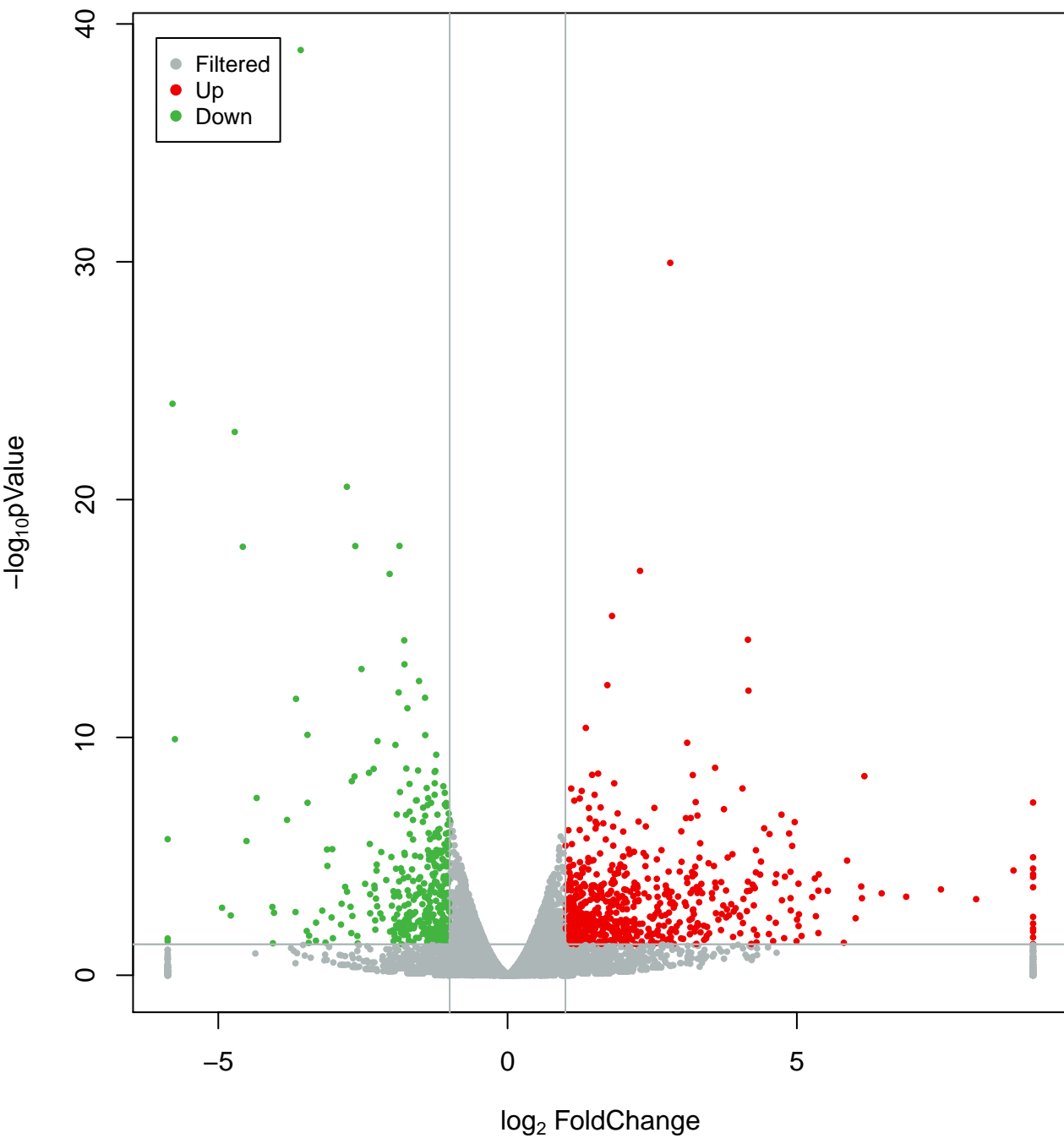

Supplement: Supplementary file 2 [file SupplementaryFile1.zip › Supplementary file 1/original RNAseq data/1.1.different_expressed_gene/MHC_6D-vs-WT_6D-volcano-pval-0.05-FC-2.gene.pdf]

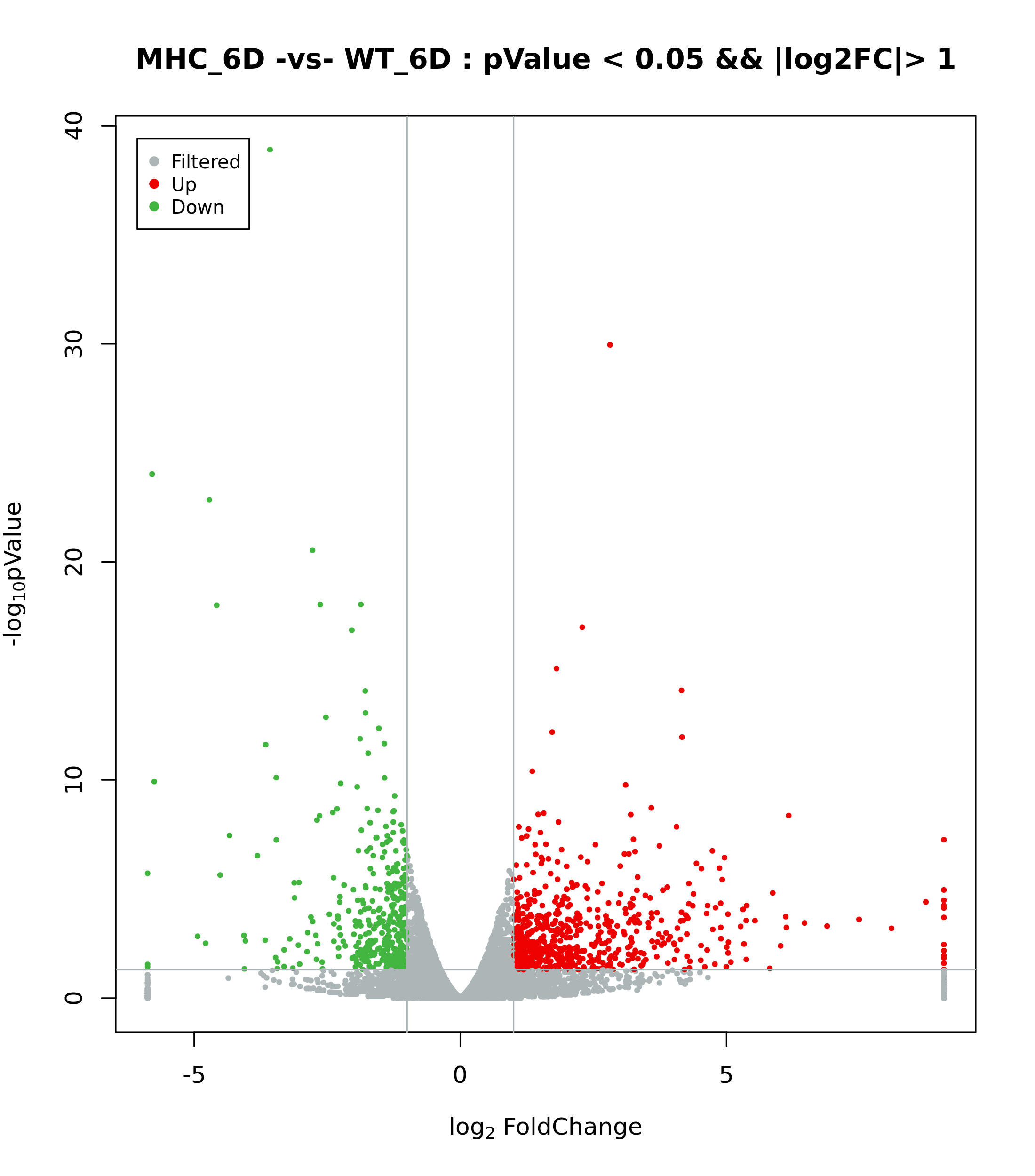

Supplement: Supplementary file 2 [file SupplementaryFile1.zip › Supplementary file 1/original RNAseq data/1.1.different_expressed_gene/MHC_6D-vs-WT_6D-volcano-pval-0.05-FC-2.gene.png]

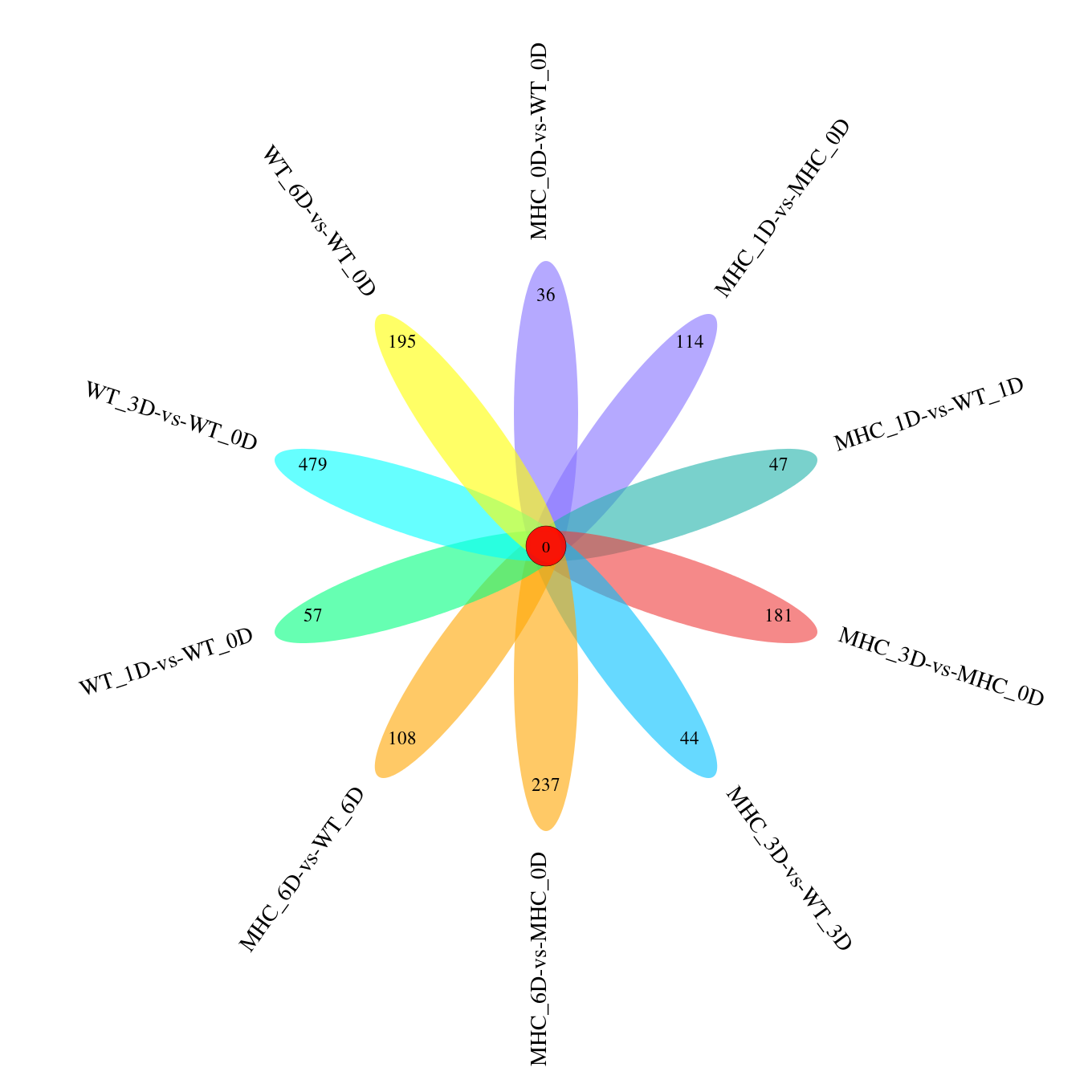

Supplement: Supplementary file 2 [file SupplementaryFile1.zip › Supplementary file 1/original RNAseq data/1.1.different_expressed_gene/VennGraph.png]

WT\_1D-vs-WT\_0D:pValue<0.05&& |log2FC|>1

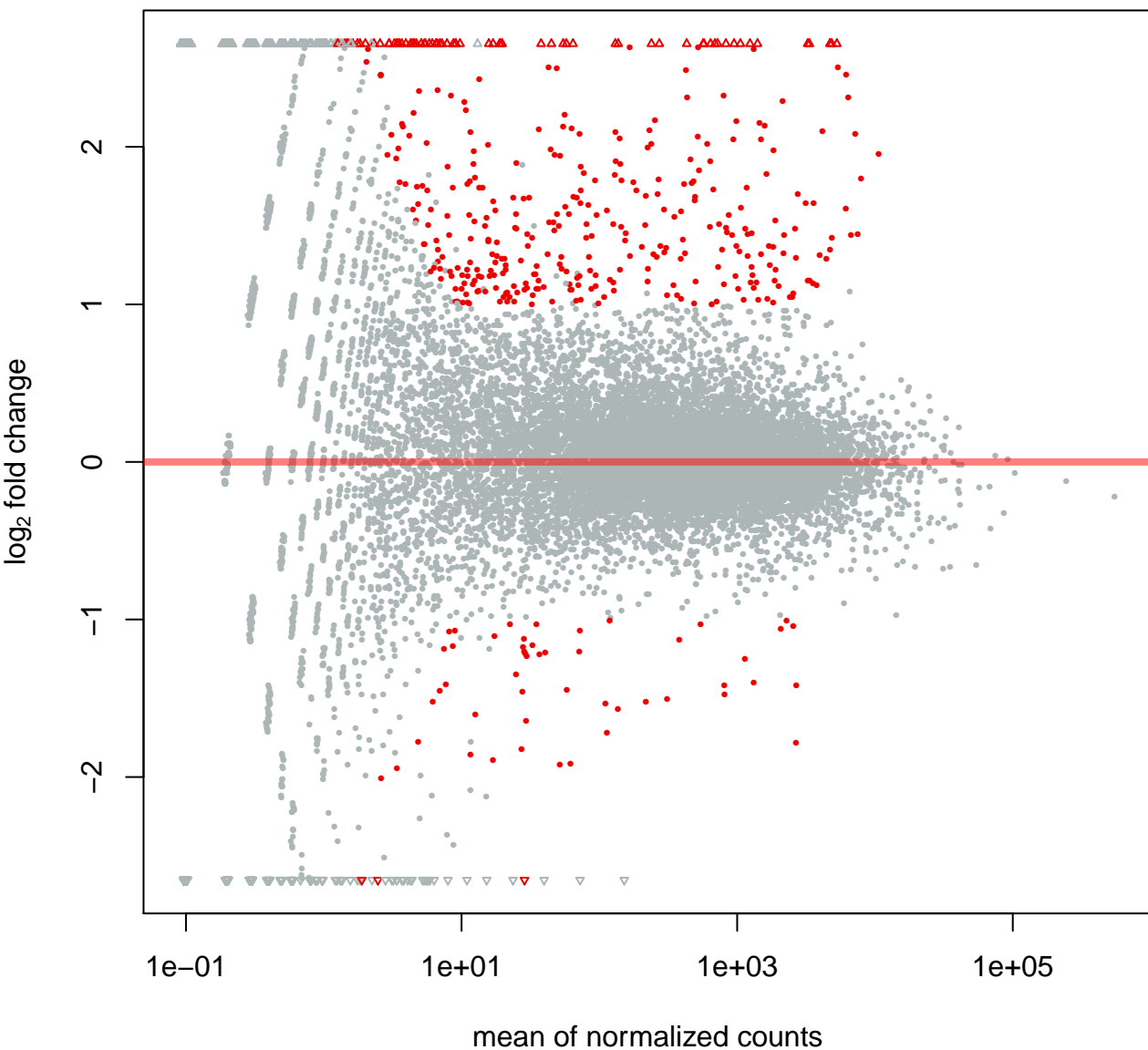

Supplement: Supplementary file 2 [file SupplementaryFile1.zip › Supplementary file 1/original RNAseq data/1.1.different_expressed_gene/WT_1D-vs-WT_0D-MA-pval-0.05-FC-2.gene.pdf]

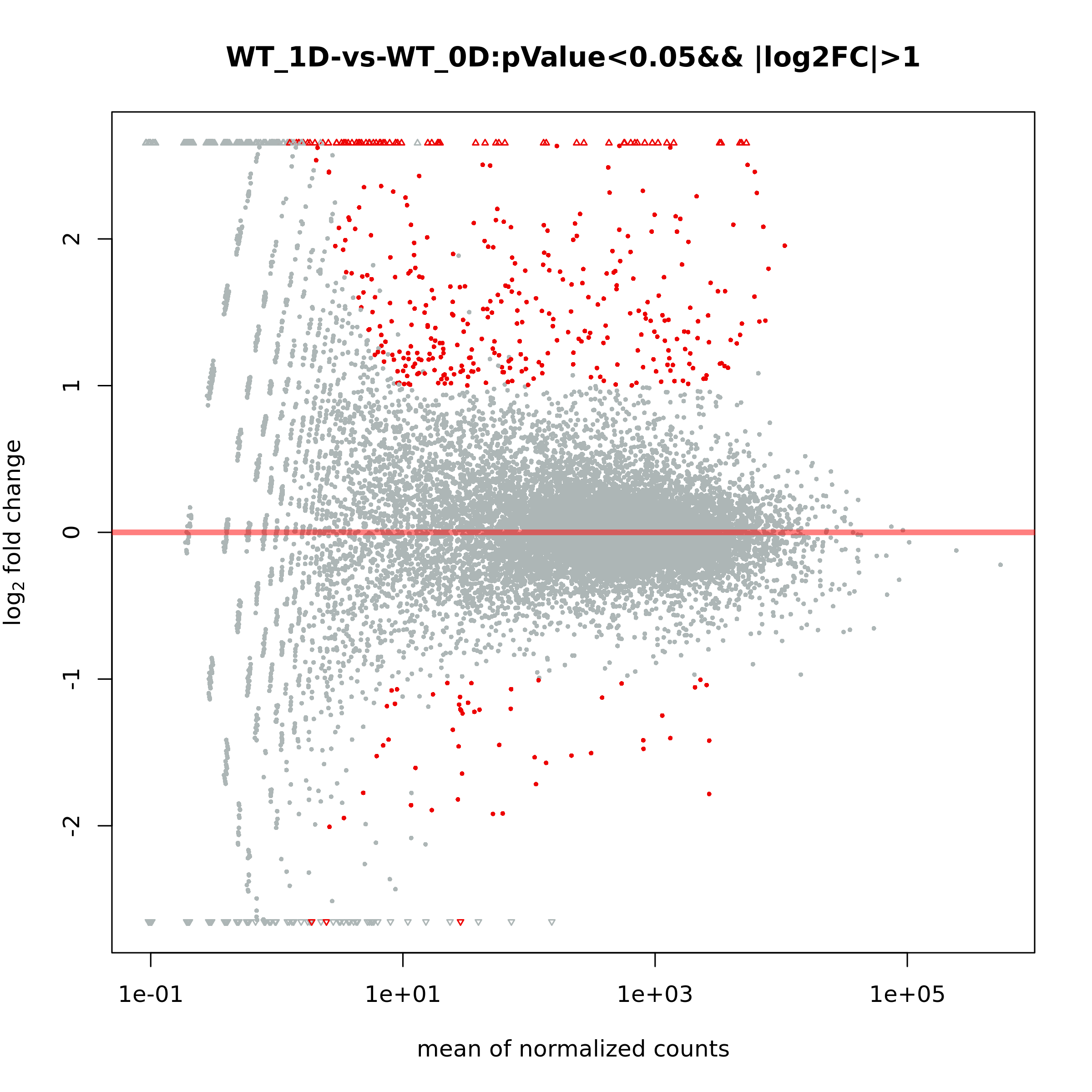

Supplement: Supplementary file 2 [file SupplementaryFile1.zip › Supplementary file 1/original RNAseq data/1.1.different_expressed_gene/WT_1D-vs-WT_0D-MA-pval-0.05-FC-2.gene.png]

WT\_1D-vs-WT\_0D:pValue<0.05&& |log2FC|>1

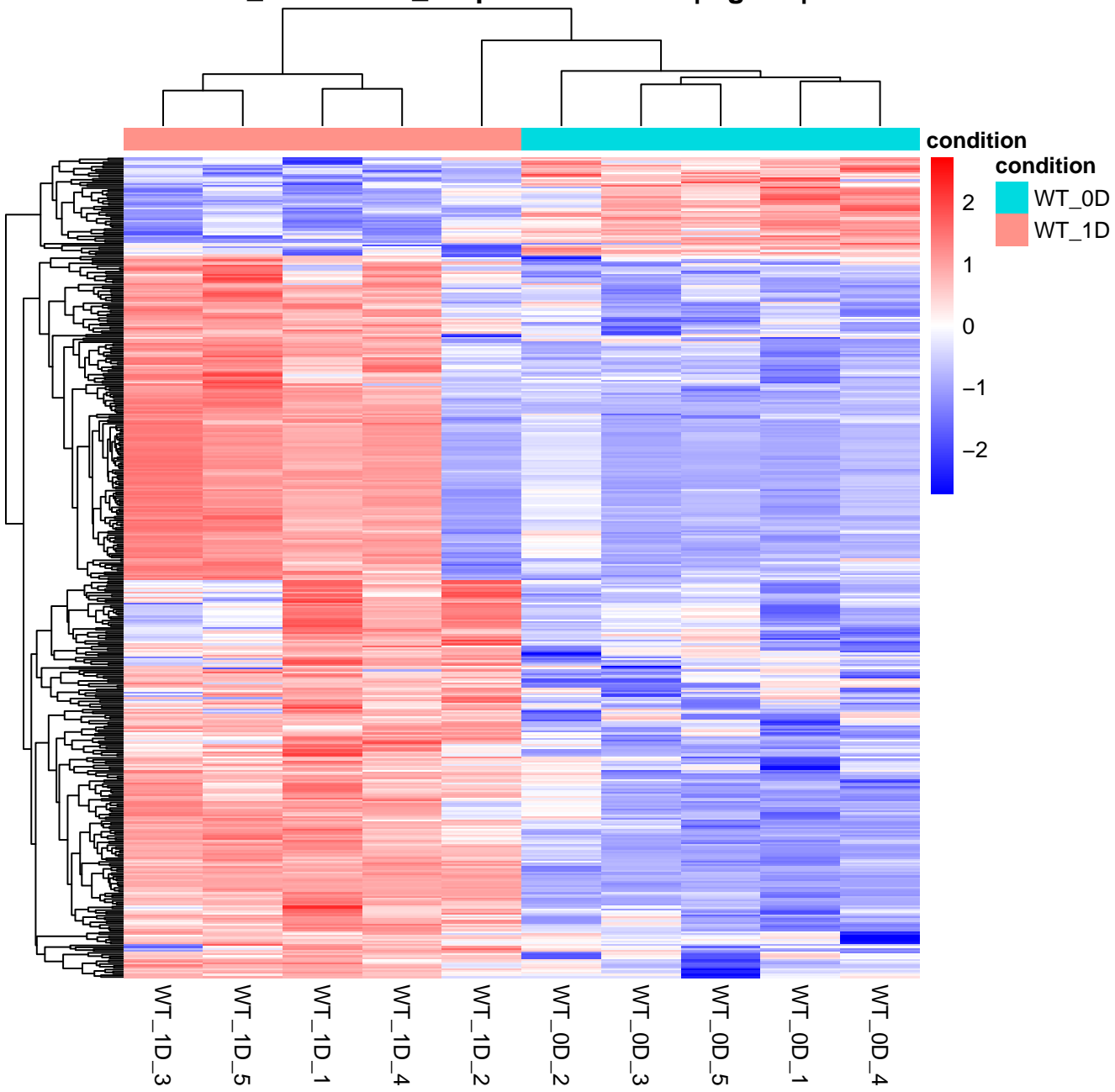

Supplement: Supplementary file 2 [file SupplementaryFile1.zip › Supplementary file 1/original RNAseq data/1.1.different_expressed_gene/WT_1D-vs-WT_0D-heatmap-pval-0.05-FC-2.gene.pdf]

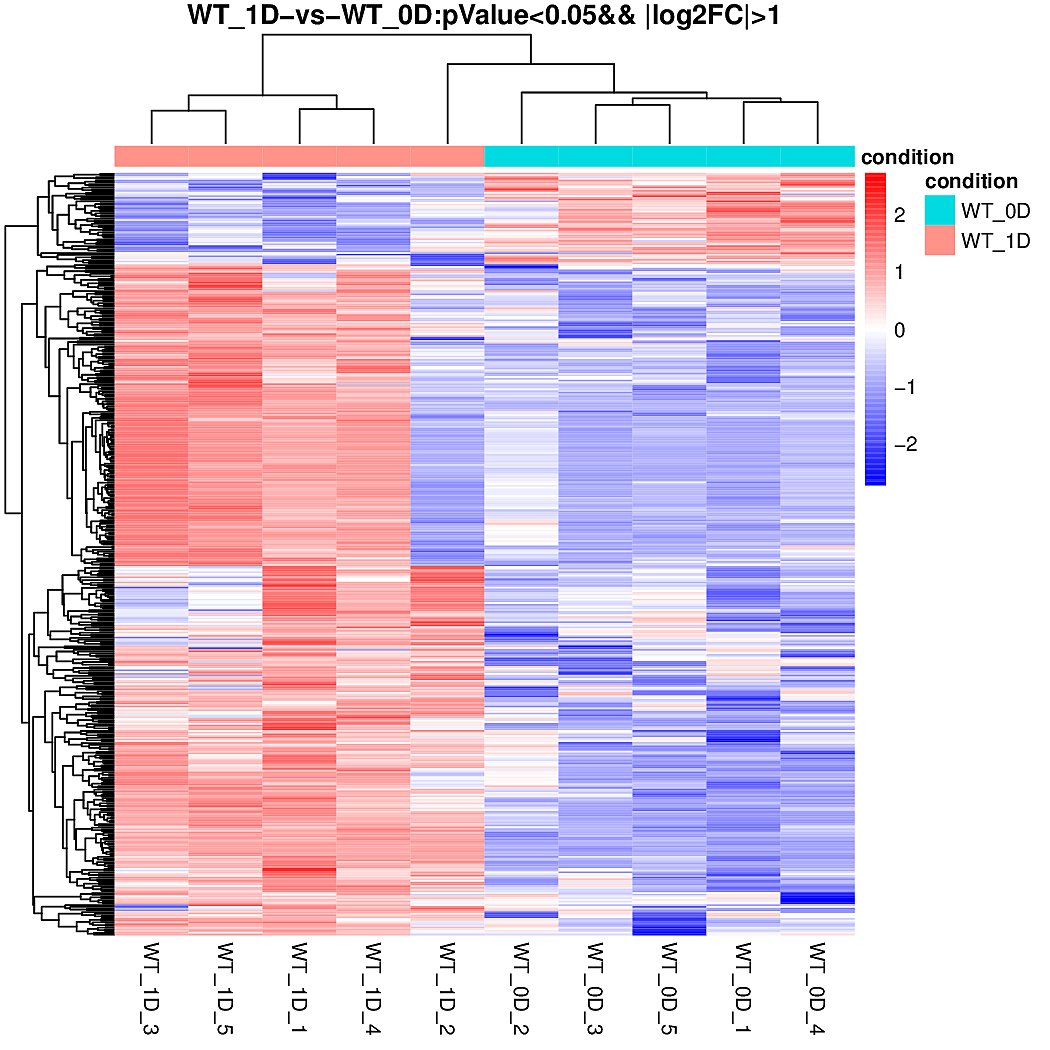

Supplement: Supplementary file 2 [file SupplementaryFile1.zip › Supplementary file 1/original RNAseq data/1.1.different_expressed_gene/WT_1D-vs-WT_0D-heatmap-pval-0.05-FC-2.gene.png]

# WT\_1D -vs- WT\_0D : pValue < 0.05 && |log2FC| > 1

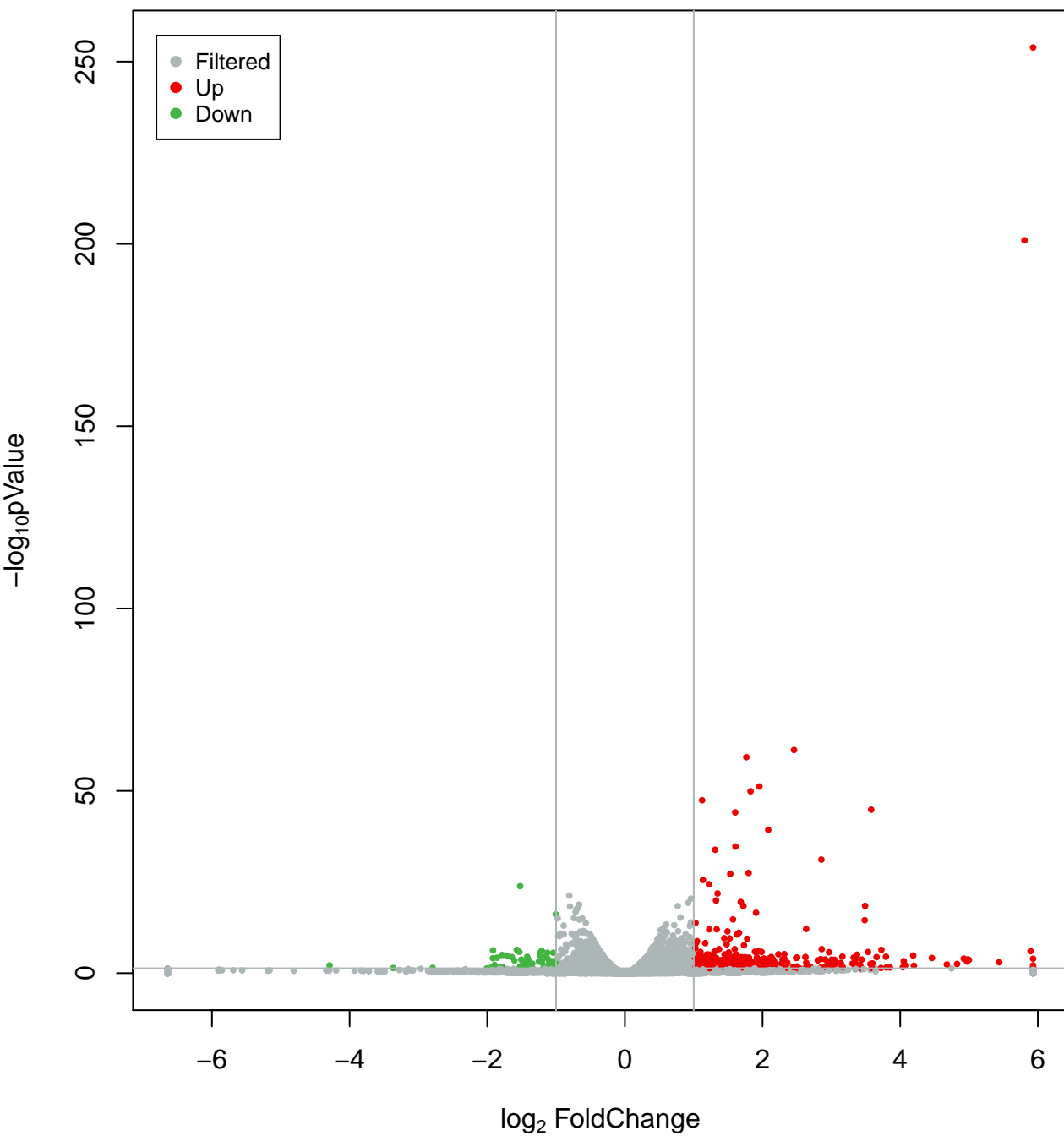

Supplement: Supplementary file 2 [file SupplementaryFile1.zip › Supplementary file 1/original RNAseq data/1.1.different_expressed_gene/WT_1D-vs-WT_0D-volcano-pval-0.05-FC-2.gene.pdf]

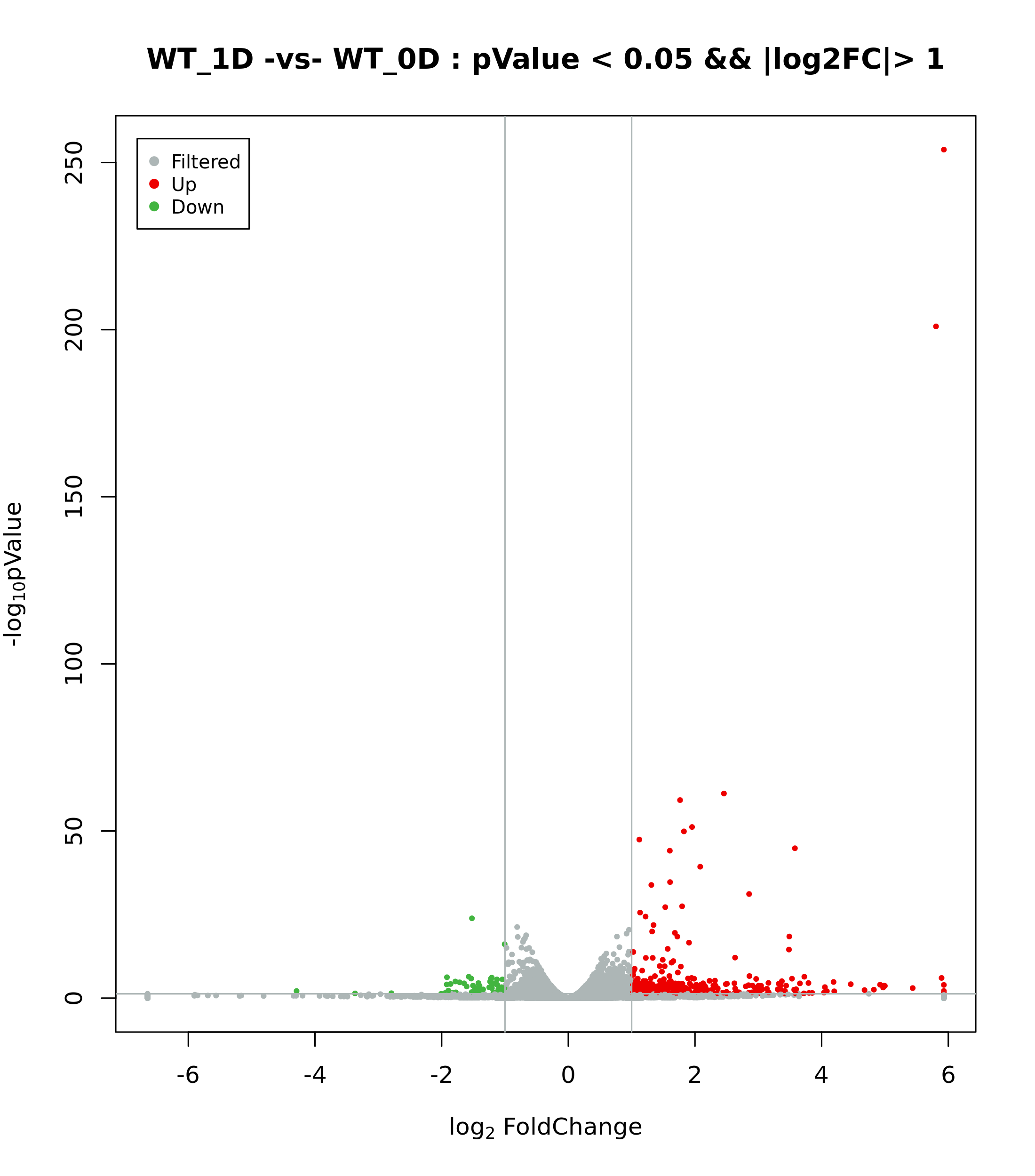

Supplement: Supplementary file 2 [file SupplementaryFile1.zip › Supplementary file 1/original RNAseq data/1.1.different_expressed_gene/WT_1D-vs-WT_0D-volcano-pval-0.05-FC-2.gene.png]

WT\_3D-vs-WT\_0D:pValue<0.05&& |log2FC|>1

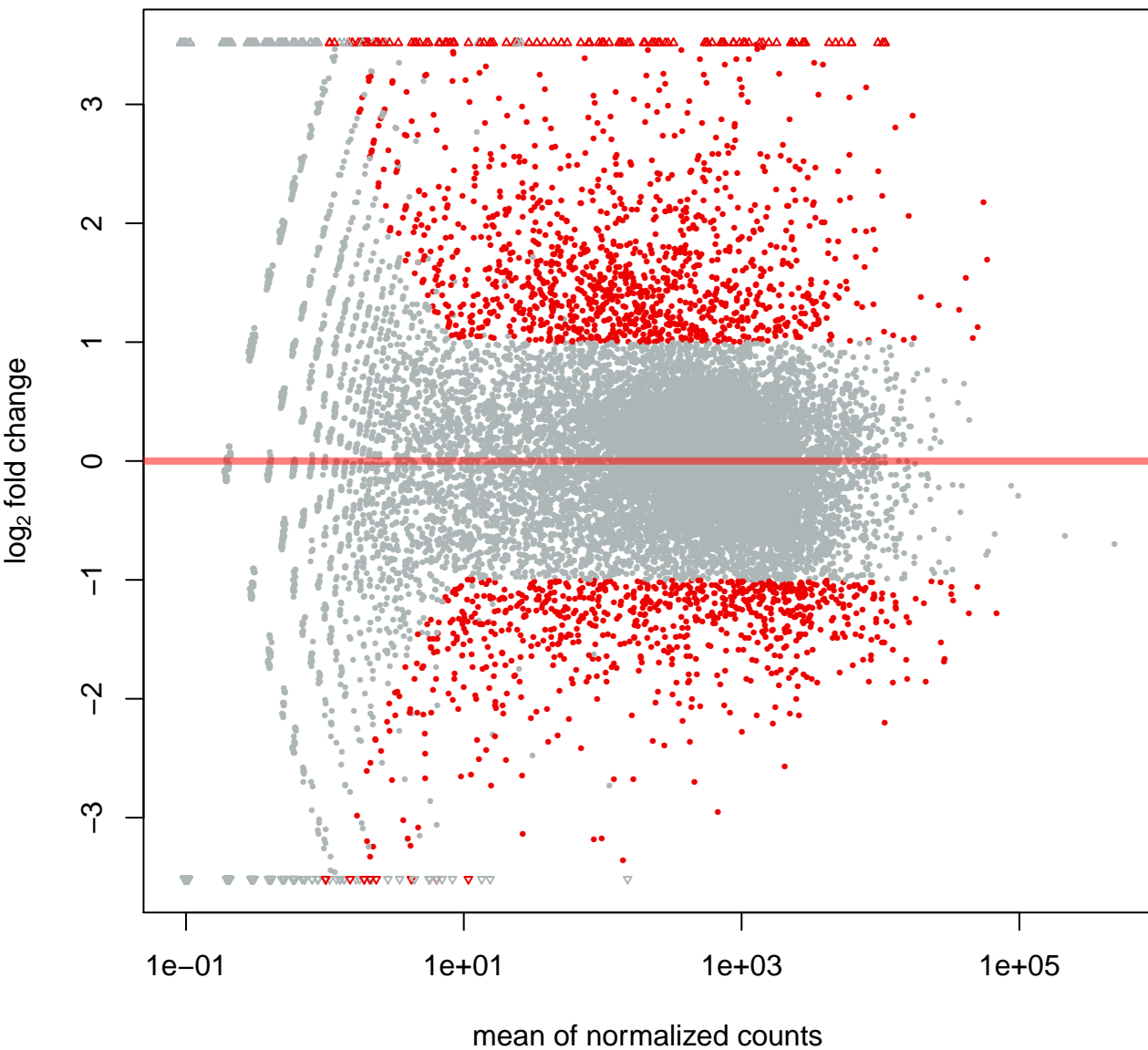

Supplement: Supplementary file 2 [file SupplementaryFile1.zip › Supplementary file 1/original RNAseq data/1.1.different_expressed_gene/WT_3D-vs-WT_0D-MA-pval-0.05-FC-2.gene.pdf]

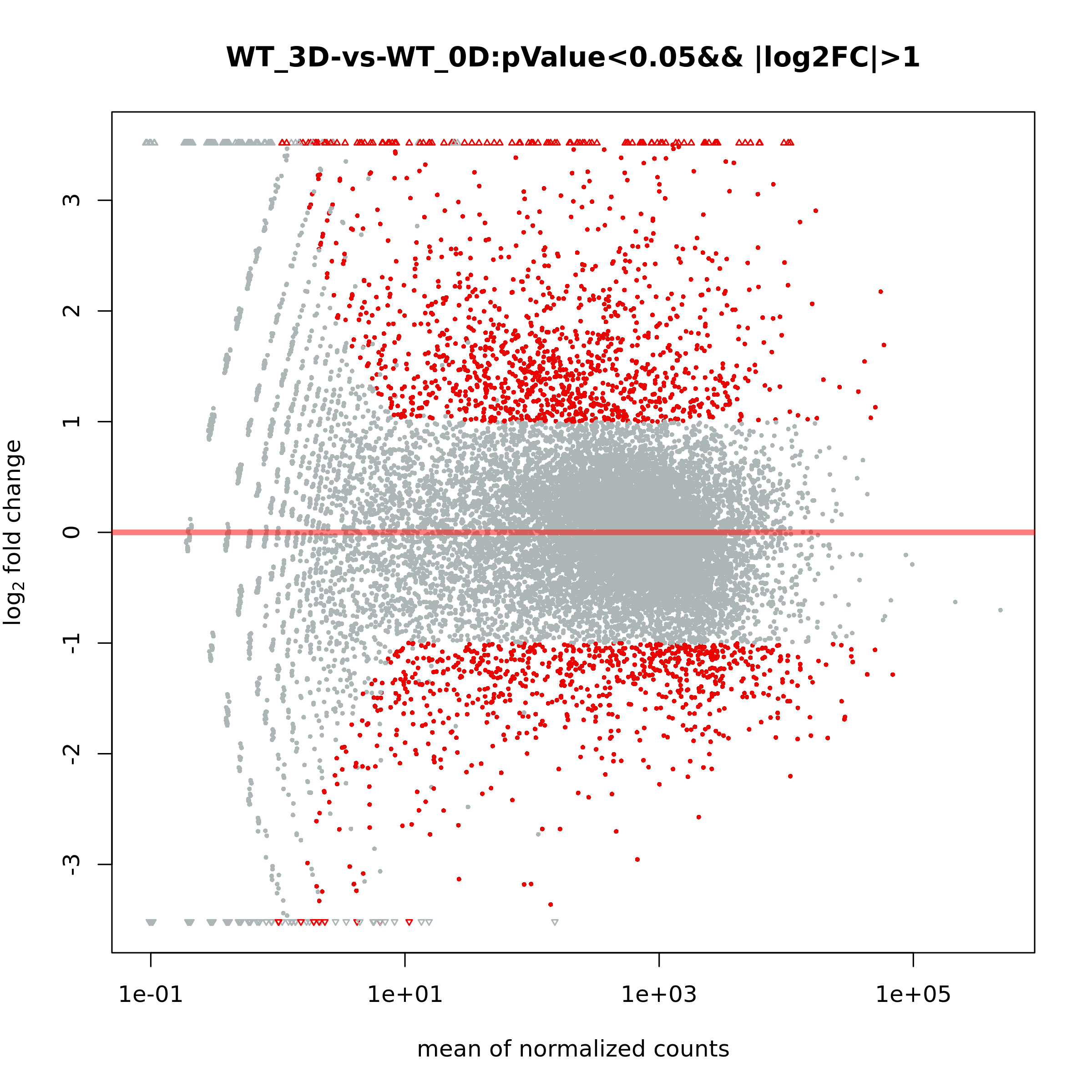

Supplement: Supplementary file 2 [file SupplementaryFile1.zip › Supplementary file 1/original RNAseq data/1.1.different_expressed_gene/WT_3D-vs-WT_0D-MA-pval-0.05-FC-2.gene.png]

WT\_3D-vs-WT\_0D:pValue<0.05&& |log2FC|>1

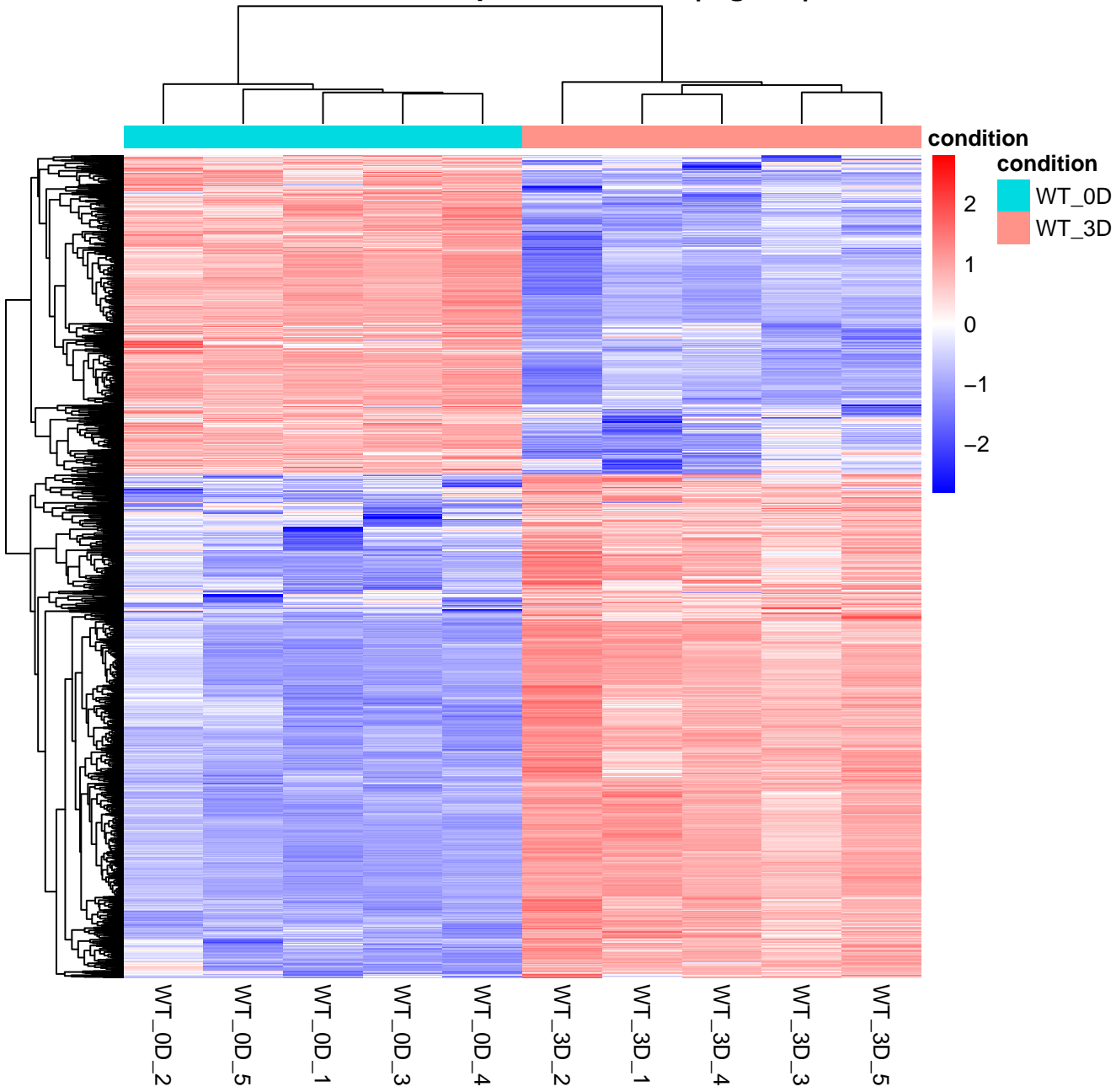

Supplement: Supplementary file 2 [file SupplementaryFile1.zip › Supplementary file 1/original RNAseq data/1.1.different_expressed_gene/WT_3D-vs-WT_0D-heatmap-pval-0.05-FC-2.gene.pdf]

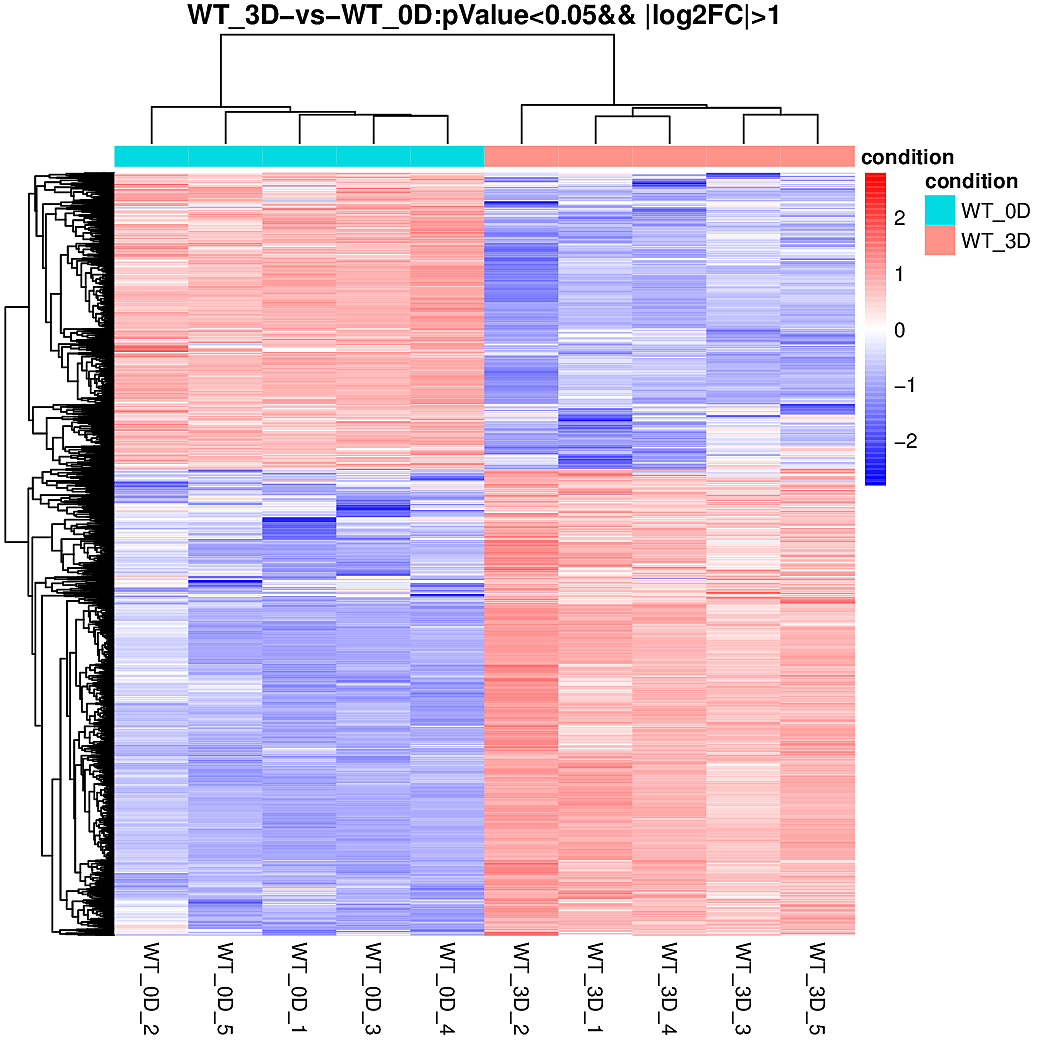

Supplement: Supplementary file 2 [file SupplementaryFile1.zip › Supplementary file 1/original RNAseq data/1.1.different_expressed_gene/WT_3D-vs-WT_0D-heatmap-pval-0.05-FC-2.gene.png]

# WT\_3D -vs- WT\_0D : pValue < 0.05 && |log2FC|> 1

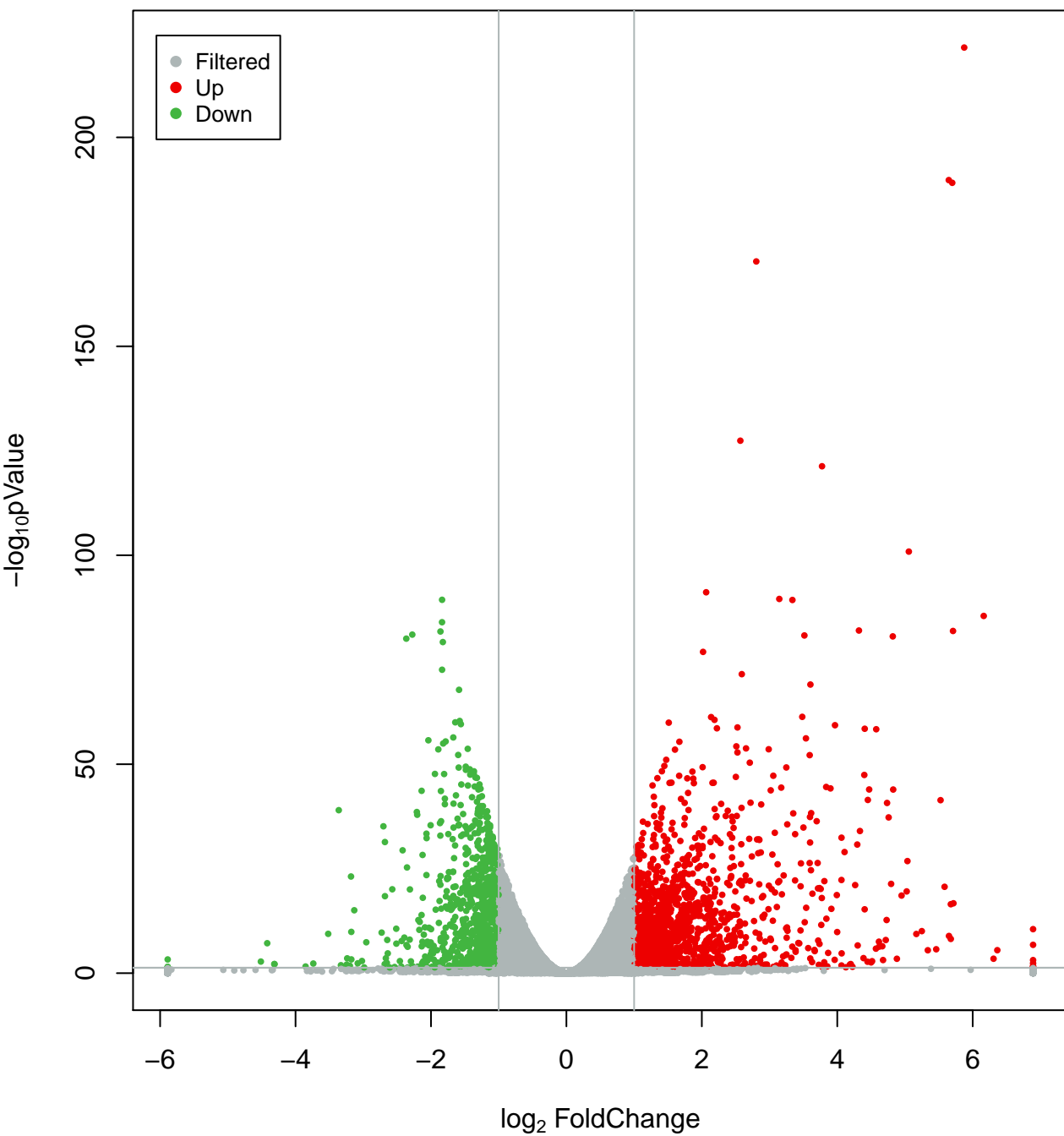

Supplement: Supplementary file 2 [file SupplementaryFile1.zip › Supplementary file 1/original RNAseq data/1.1.different_expressed_gene/WT_3D-vs-WT_0D-volcano-pval-0.05-FC-2.gene.pdf]

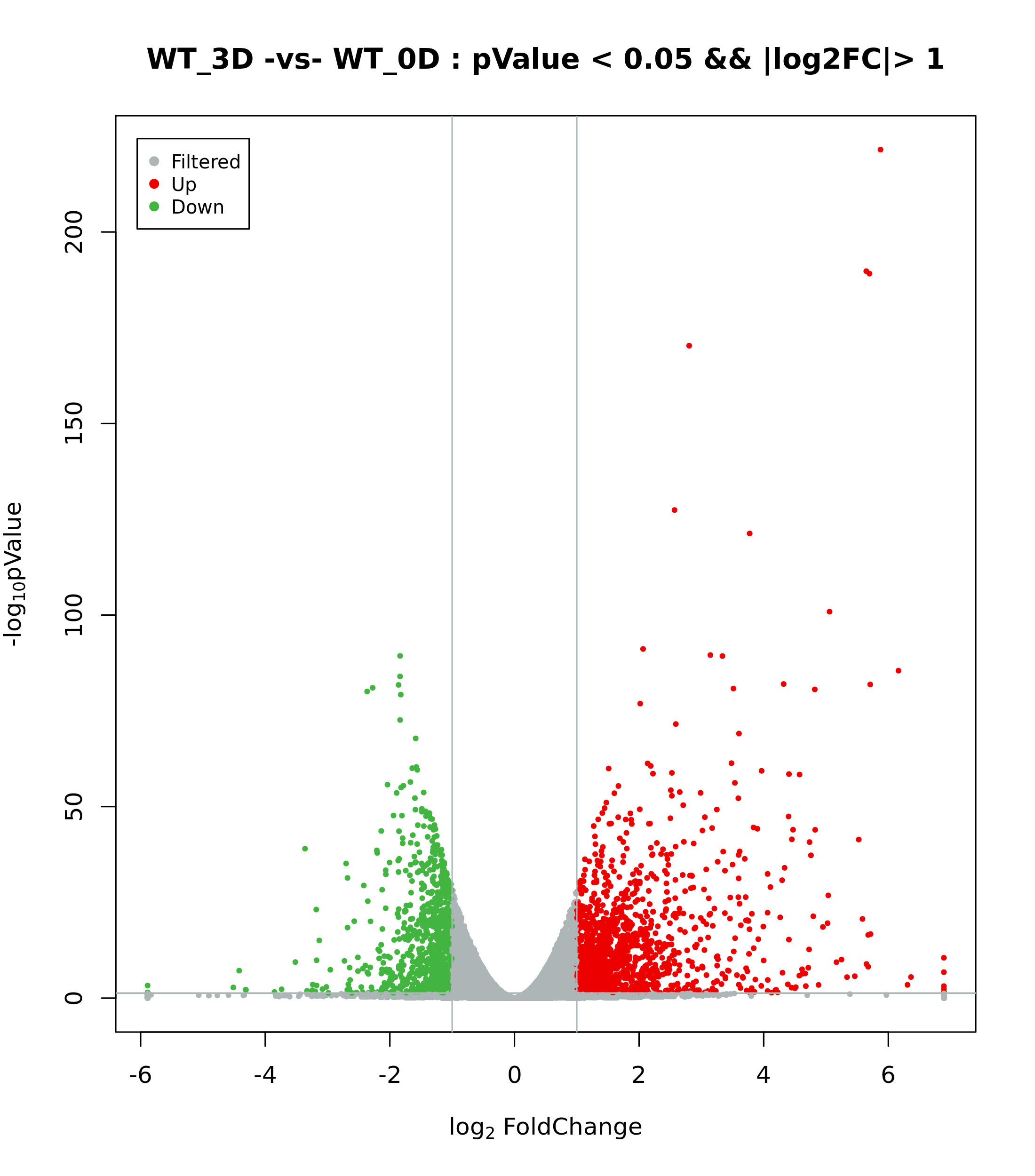

Supplement: Supplementary file 2 [file SupplementaryFile1.zip › Supplementary file 1/original RNAseq data/1.1.different_expressed_gene/WT_3D-vs-WT_0D-volcano-pval-0.05-FC-2.gene.png]

WT\_6D-vs-WT\_0D:pValue<0.05&& |log2FC|>1

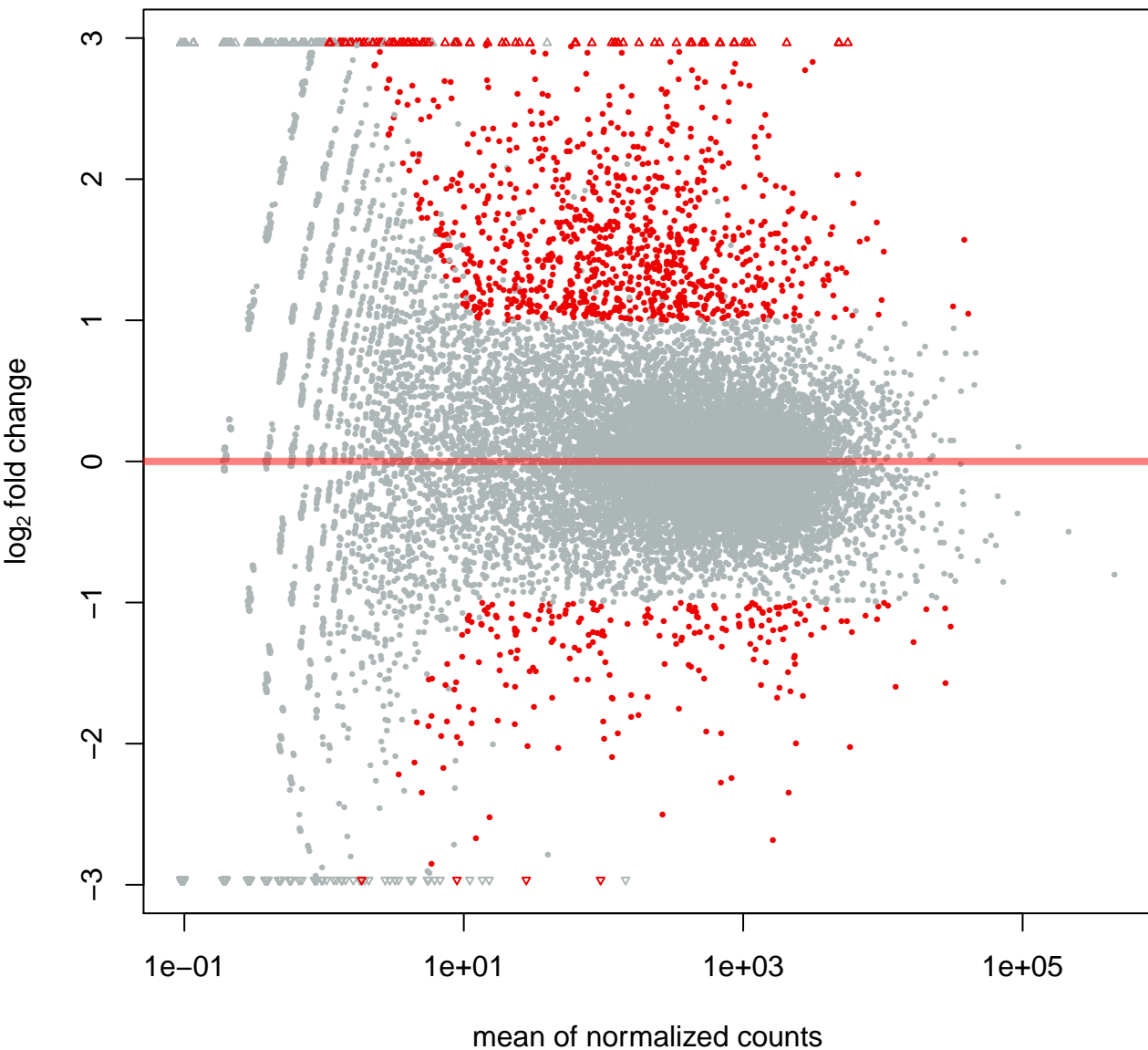

Supplement: Supplementary file 2 [file SupplementaryFile1.zip › Supplementary file 1/original RNAseq data/1.1.different_expressed_gene/WT_6D-vs-WT_0D-MA-pval-0.05-FC-2.gene.pdf]

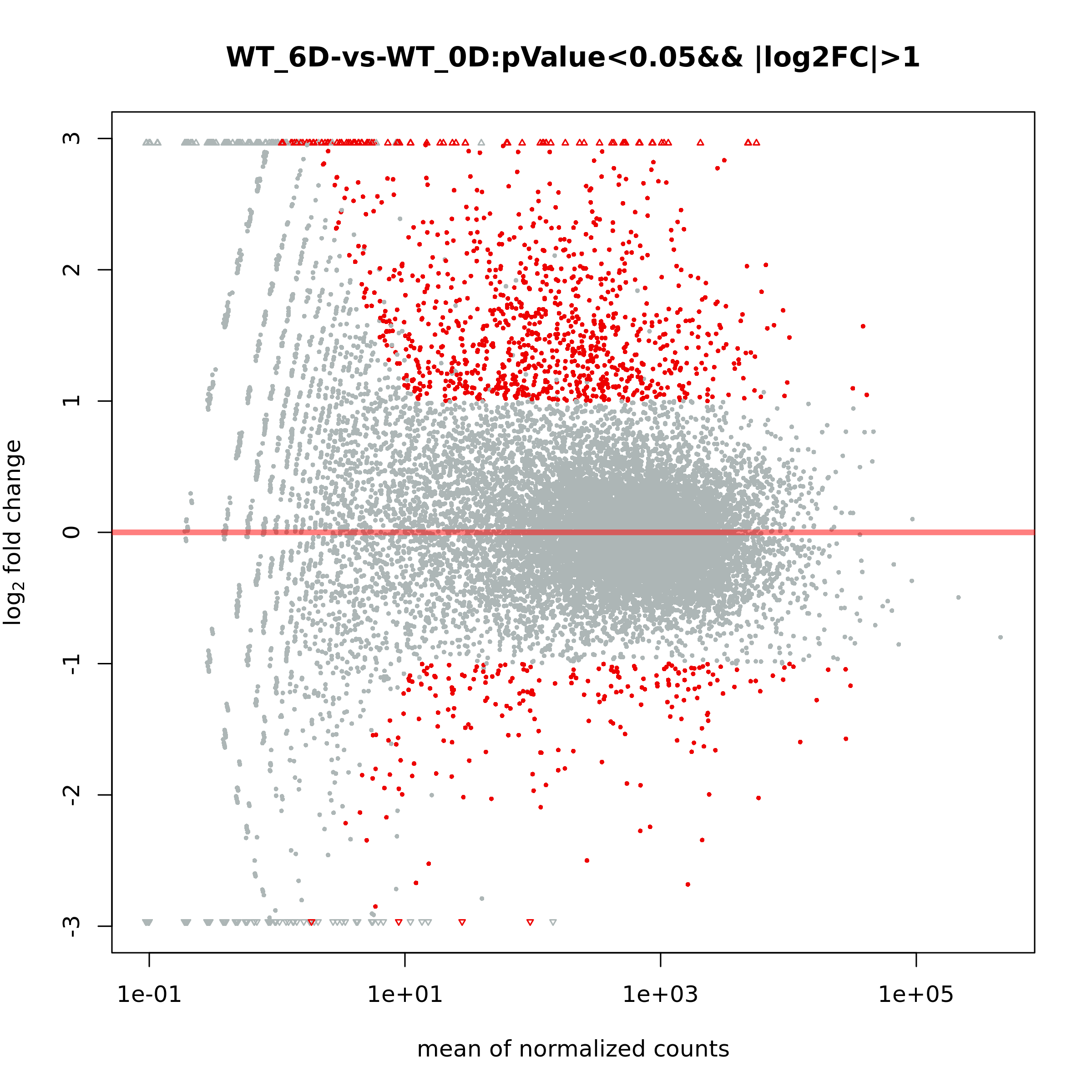

Supplement: Supplementary file 2 [file SupplementaryFile1.zip › Supplementary file 1/original RNAseq data/1.1.different_expressed_gene/WT_6D-vs-WT_0D-MA-pval-0.05-FC-2.gene.png]

WT\_6D-vs-WT\_0D:pValue<0.05&& |log2FC|>1

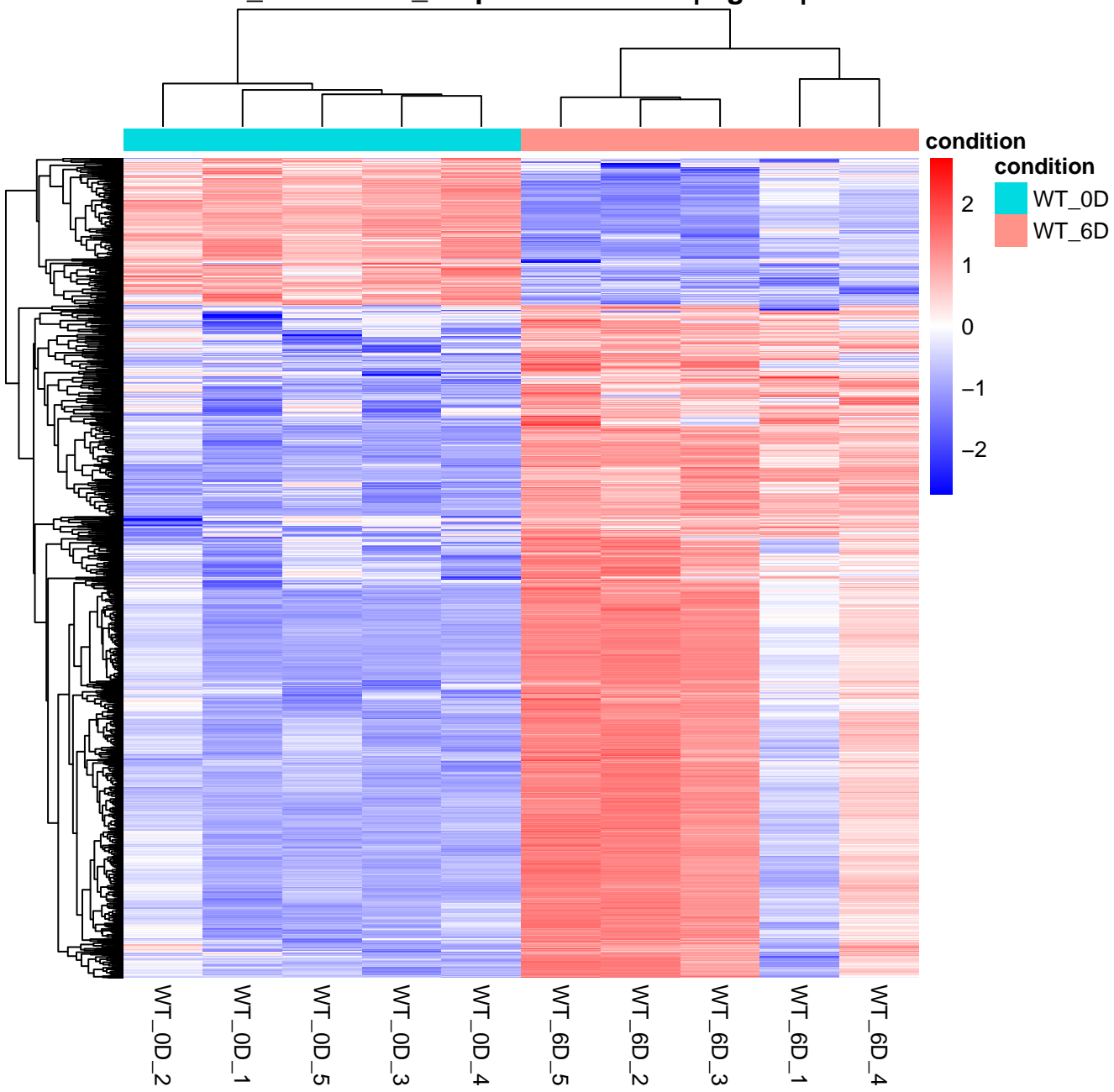

Supplement: Supplementary file 2 [file SupplementaryFile1.zip › Supplementary file 1/original RNAseq data/1.1.different_expressed_gene/WT_6D-vs-WT_0D-heatmap-pval-0.05-FC-2.gene.pdf]

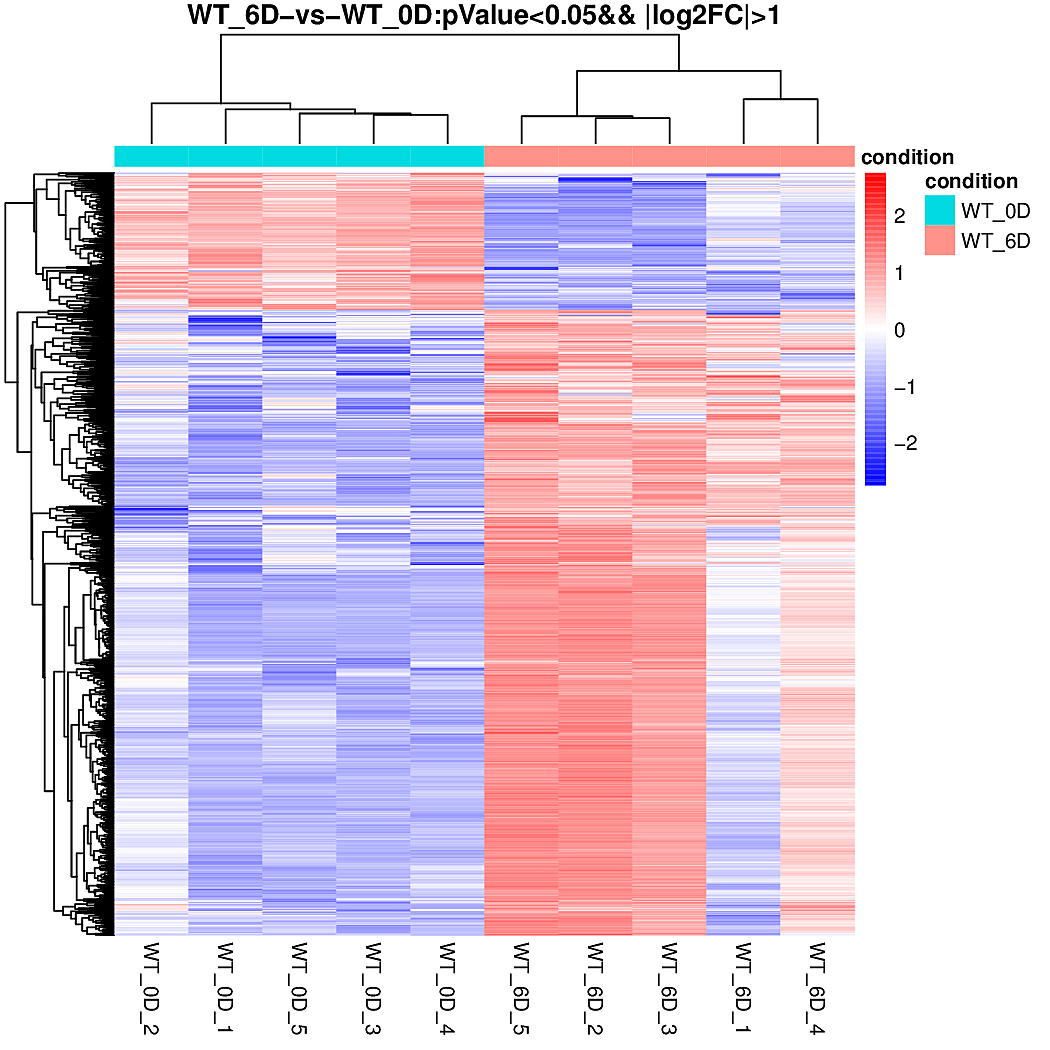

Supplement: Supplementary file 2 [file SupplementaryFile1.zip › Supplementary file 1/original RNAseq data/1.1.different_expressed_gene/WT_6D-vs-WT_0D-heatmap-pval-0.05-FC-2.gene.png]

# WT\_6D -vs- WT\_0D : pValue < 0.05 && |log2FC|> 1

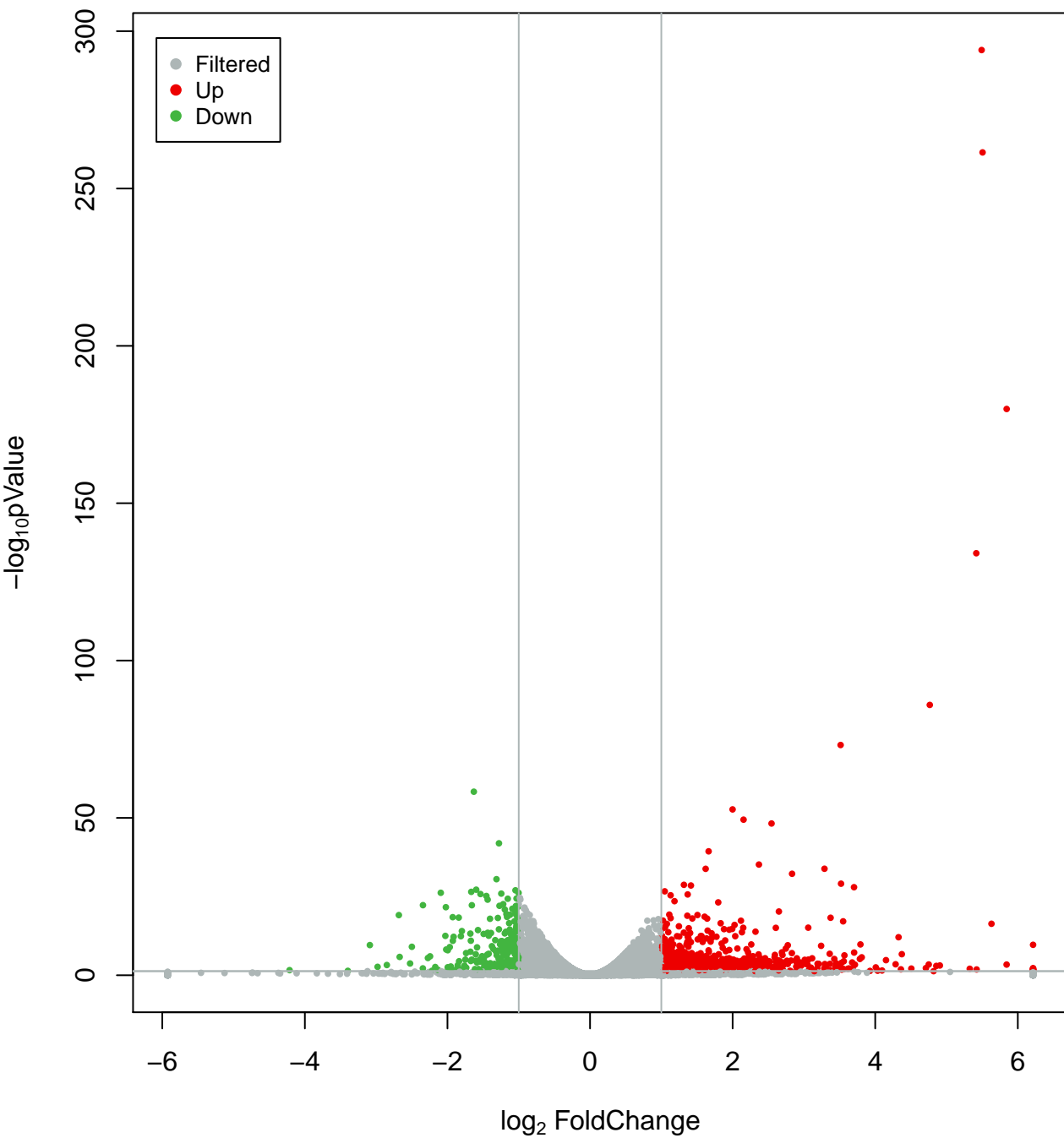

Supplement: Supplementary file 2 [file SupplementaryFile1.zip › Supplementary file 1/original RNAseq data/1.1.different_expressed_gene/WT_6D-vs-WT_0D-volcano-pval-0.05-FC-2.gene.pdf]

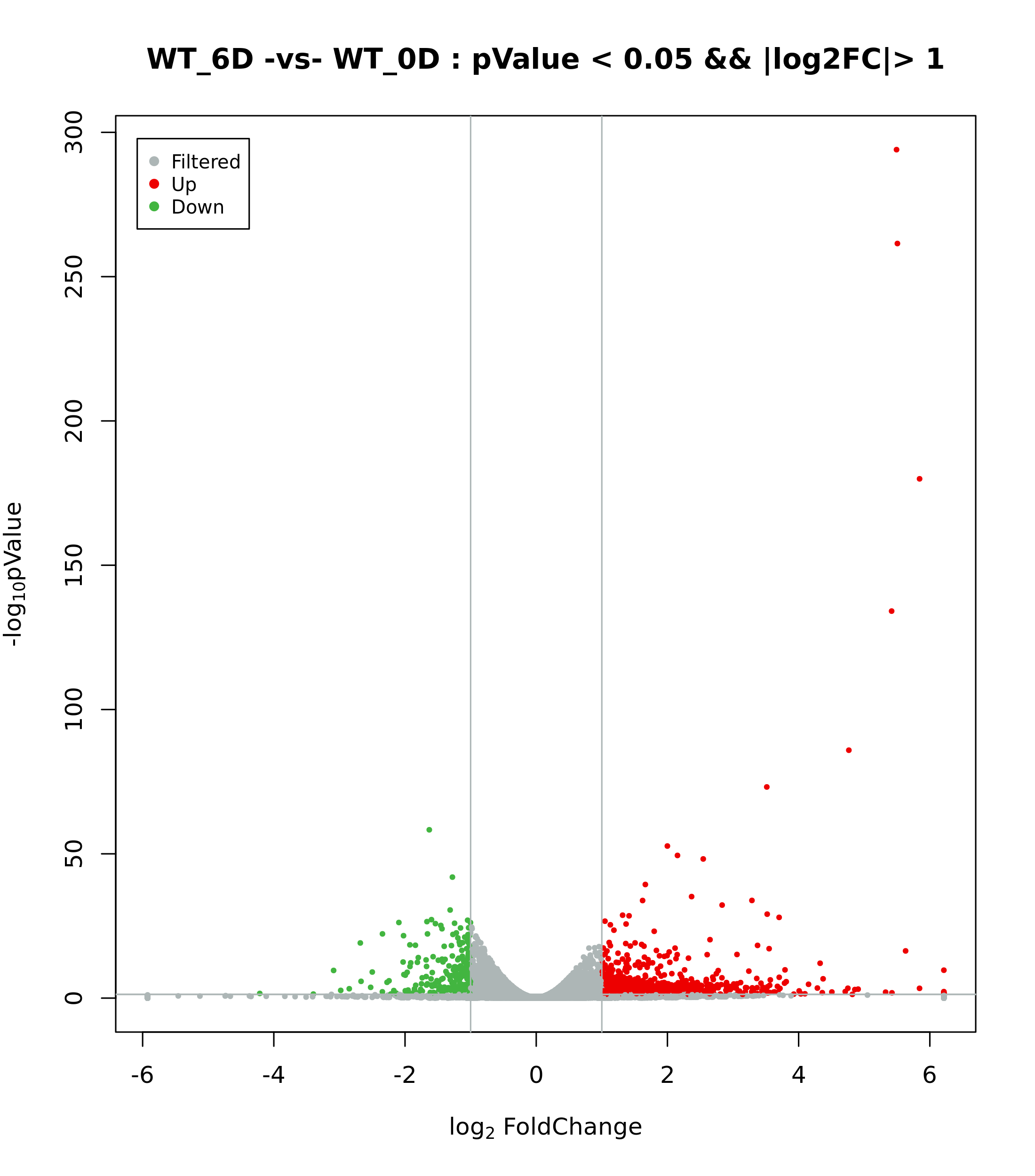

Supplement: Supplementary file 2 [file SupplementaryFile1.zip › Supplementary file 1/original RNAseq data/1.1.different_expressed_gene/WT_6D-vs-WT_0D-volcano-pval-0.05-FC-2.gene.png]

Statistic of Differently Expressed gene

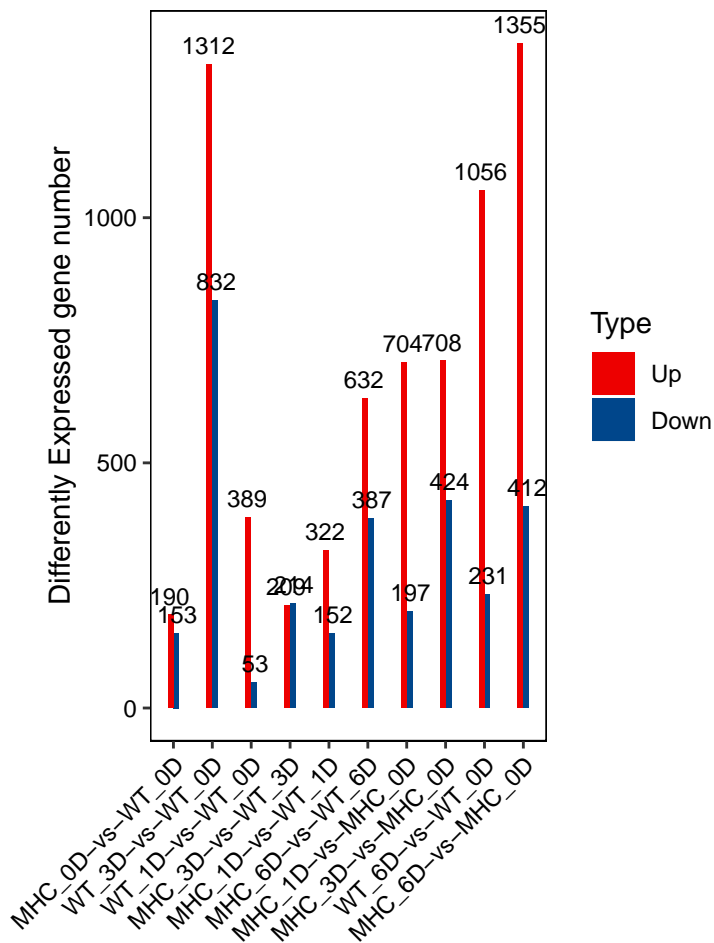

Supplement: Supplementary file 2 [file SupplementaryFile1.zip › Supplementary file 1/original RNAseq data/1.1.different_expressed_gene/gene_diff_stat_barplot.pdf]

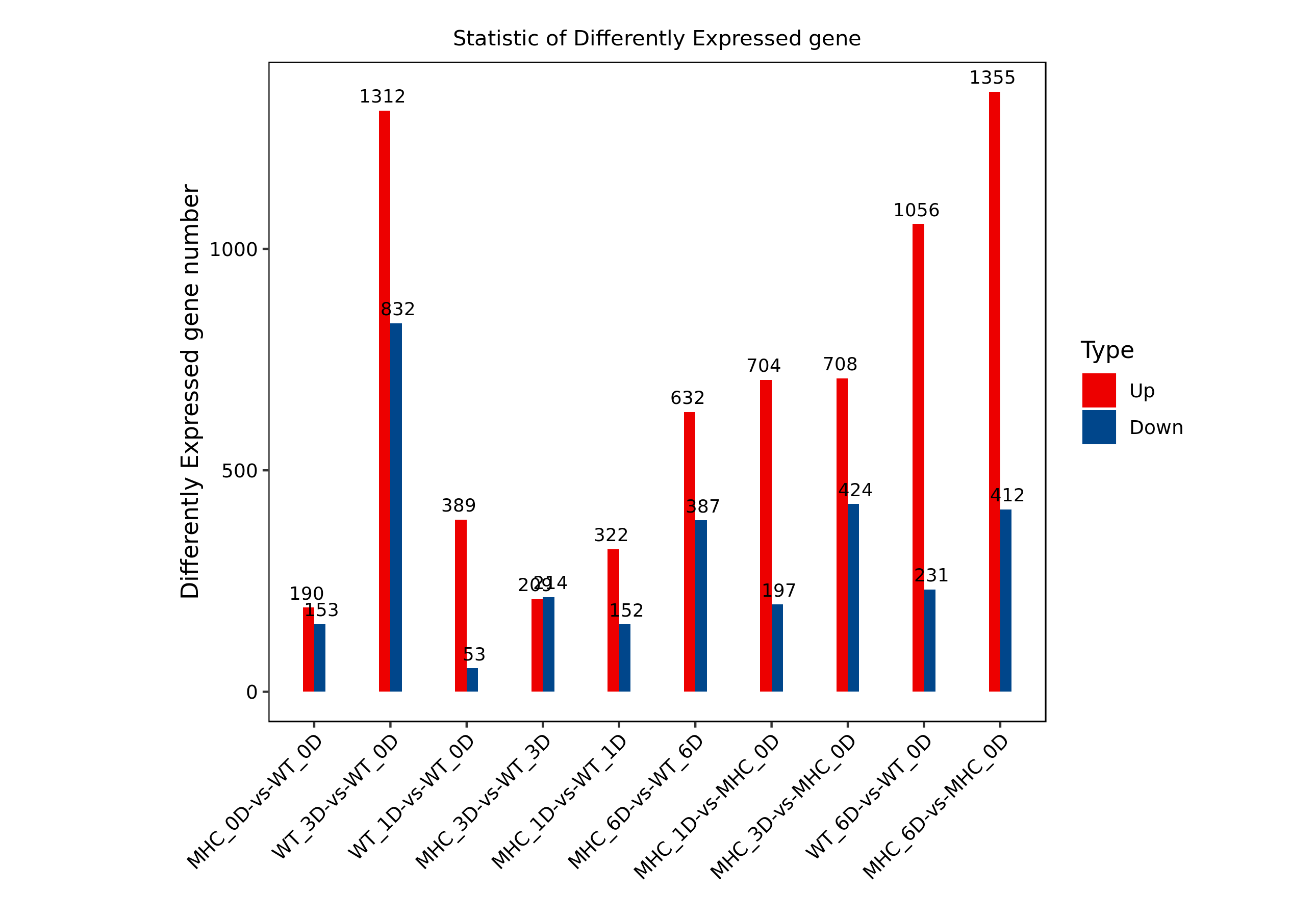

Supplement: Supplementary file 2 [file SupplementaryFile1.zip › Supplementary file 1/original RNAseq data/1.1.different_expressed_gene/gene_diff_stat_barplot.png]
